# Differential transcriptomic profiling of filamentous fungus during solid-state and submerged fermentation and identification of an essential regulatory gene *PoxMBF1* that directly regulated cellulase and xylanase gene expression

**DOI:** 10.1186/s13068-019-1445-4

**Published:** 2019-04-30

**Authors:** Shuai Zhao, Qi Liu, Jiu-Xiang Wang, Xu-Zhong Liao, Hao Guo, Cheng-Xi Li, Feng-Fei Zhang, Lu-Sheng Liao, Xue-Mei Luo, Jia-Xun Feng

**Affiliations:** State Key Laboratory for Conservation and Utilization of Subtropical Agro-bioresources, Guangxi Research Center for Microbial and Enzyme Engineering Technology, College of Life Science and Technology, Guangxi University, 100 Daxue Road, Nanning, 530004 Guangxi People’s Republic of China

**Keywords:** Transcriptional regulation, Cellulase, Xylanase, Solid-state fermentation, Submerged fermentation, *Penicillium oxalicum*

## Abstract

**Background:**

Solid-state fermentation (SSF) mimics the natural decay environment of soil fungi and can be employed to investigate the production of plant biomass-degrading enzymes. However, knowledge on the transcriptional regulation of fungal genes during SSF remains limited. Herein, transcriptional profiling was performed on the filamentous fungus *Penicillium oxalicum* strain HP7-1 cultivated in medium containing wheat bran plus rice straw (WR) under SSF (WR_SSF) and submerged fermentation (WR_SmF; control) conditions. Novel key transcription factors (TFs) regulating fungal cellulase and xylanase gene expression during SSF were identified via comparative transcriptomic and genetic analyses.

**Results:**

Expression of major cellulase genes was higher under WR_SSF condition than that under WR_SmF, but the expression of genes involved in the citric acid cycle was repressed under WR_SSF condition. Fifty-six candidate regulatory genes for cellulase production were screened out from transcriptomic profiling of *P*. *oxalicum* HP7-1 for knockout experiments in the parental strain ∆*PoxKu70*, resulting in 43 deletion mutants including 18 constructed in the previous studies. Enzyme activity assays revealed 14 novel regulatory genes involved in cellulase production in *P*. *oxalicum* during SSF. Remarkably, deletion of the essential regulatory gene *PoxMBF1*, encoding Multiprotein Bridging Factor 1, resulted in doubled cellulase and xylanase production at 2 days after induction during both SSF and SmF. *PoxMBF1* dynamically and differentially regulated transcription of a subset of cellulase and xylanase genes during SSF and SmF, and conferred stress resistance. Importantly, PoxMBF1 bound specifically to the putative promoters of major cellulase and xylanase genes in vitro.

**Conclusions:**

We revealed differential transcriptional regulation of *P*. *oxalicum* during SSF and SmF, and identified PoxMBF1, a novel TF that directly regulates cellulase and xylanase gene expression during SSF and SmF. These findings expand our understanding of regulatory mechanisms of cellulase and xylanase gene expression during fungal fermentation.

**Electronic supplementary material:**

The online version of this article (10.1186/s13068-019-1445-4) contains supplementary material, which is available to authorized users.

## Background

As decomposers, soil fungi play essential roles in global carbon cycling due to their ability to produce enzymes that degrade plant biomass into monosaccharides such as glucose and xylose. Solid-state fermentation (SSF) mimics the natural decay environment of soil fungi, and can be used to investigate production of plant biomass-degrading enzymes, the global carbon cycle, and commercial production of high value-added bioproducts including ancient Chinese liquor, soy sauce, vinegar, penicillin and other antibiotics, pigments, and environmentally friendly sources of alternative energy [1, 2]. Compared with submerged fermentation (SmF), SSF has various advantages including direct use of agricultural and industrial residues as carbon sources, resulting in lower cost.

The filamentous fungus *Penicillium oxalicum* secretes integrated cellulolytic and xylolytic enzymes, and can be used as an alternative to the industrial fungal strain *Trichoderma reesei* owing to its high β-glucosidase (EC 3.2.1.21) activity [3]. Production of fungal cellulases and xylanases not only depends on stimulation of exogenous carbon sources under certain cultivation conditions; it is also strictly controlled by regulatory networks comprising numerous transcriptional factors (TFs), signal sensors, and receptors. For example, culturing of *P*. *oxalicum* strain EU2106 in medium containing wheat bran (WB) under SSF reportedly achieved higher cellulase production than in medium containing corncob, rice husk, rice straw, or sugarcane bagasse [4]. Furthermore, induction of *P*. *oxalicum* strain HP7-1 using WB plus Avicel under SmF proved more favourable for cellulase and xylanase production than induction using WB alone [3].

Experiments showed higher expression of the glucoamylase gene *glaB* in *Aspergillus oryzae* cultivated on wheat-based solid medium than wheat-based liquid medium [5]. Moreover, transcriptional levels of laccase genes from *Pleurotus ostreatus* are upregulated under SmF conditions containing wheat straw, but downregulated under SSF [6]. However, systematic analysis of genome-wide gene expression in filamentous fungi under different cultivation conditions, including SSF and SmF, is scare.

Most recent studies on TFs regulating the expression of genes encoding cellulolytic and xylolytic enzymes have mainly focused on filamentous fungi, including *Trichoderma*, *Aspergillus*, *Penicillium*, and *Neurospora crassa*, under SmF condition, such as transcriptional activators CLR-2/ClrB [3, 7, 8], PoxCxrA [9], and the carbon catabolite repressor CreA/CRE1/CRE-1 [10, 11]. Nevertheless, knowledge of the regulatory roles of TFs in cellulase and xylanase gene expression in filamentous fungi during SSF remains limited.

In the present study, the transcriptomes of *P*. *oxalicum* during SSF and SmF in medium containing wheat bran plus rice straw (WR) were comparatively analysed. Candidate genes potentially regulating cellulase and xylanase production in *P. oxalicum* during SSF were screened out and knocked out in the parental strain ∆*PoxKu70*. The essential gene *PoxMBF1*, encoding Multiprotein Bridging Factor 1, was found to control cellulase and xylanase gene expression in *P*. *oxalicum* during SSF and SmF via genetic and biochemical analyses.

## Results

### Comparative transcriptomic analysis of *P. oxalicum* strain HP7-1 during SSF and SmF

The *P*. *oxalicum* wild-type strain HP7-1 was, respectively, cultivated in solid and liquid media containing WR as the carbon source for 24 h, and total RNA was extracted and subjected to RNA sequencing (RNA-Seq) on an Illumina HiSeq 2000 system. Approximately 24 million clean reads were generated for each sample, and the length of each read was 100 bp. More than 90% of clean reads were mapped onto the genome of *P*. *oxalicum* strain HP7-1 (Additional file 1: Table S1).

**Fig. 1:**
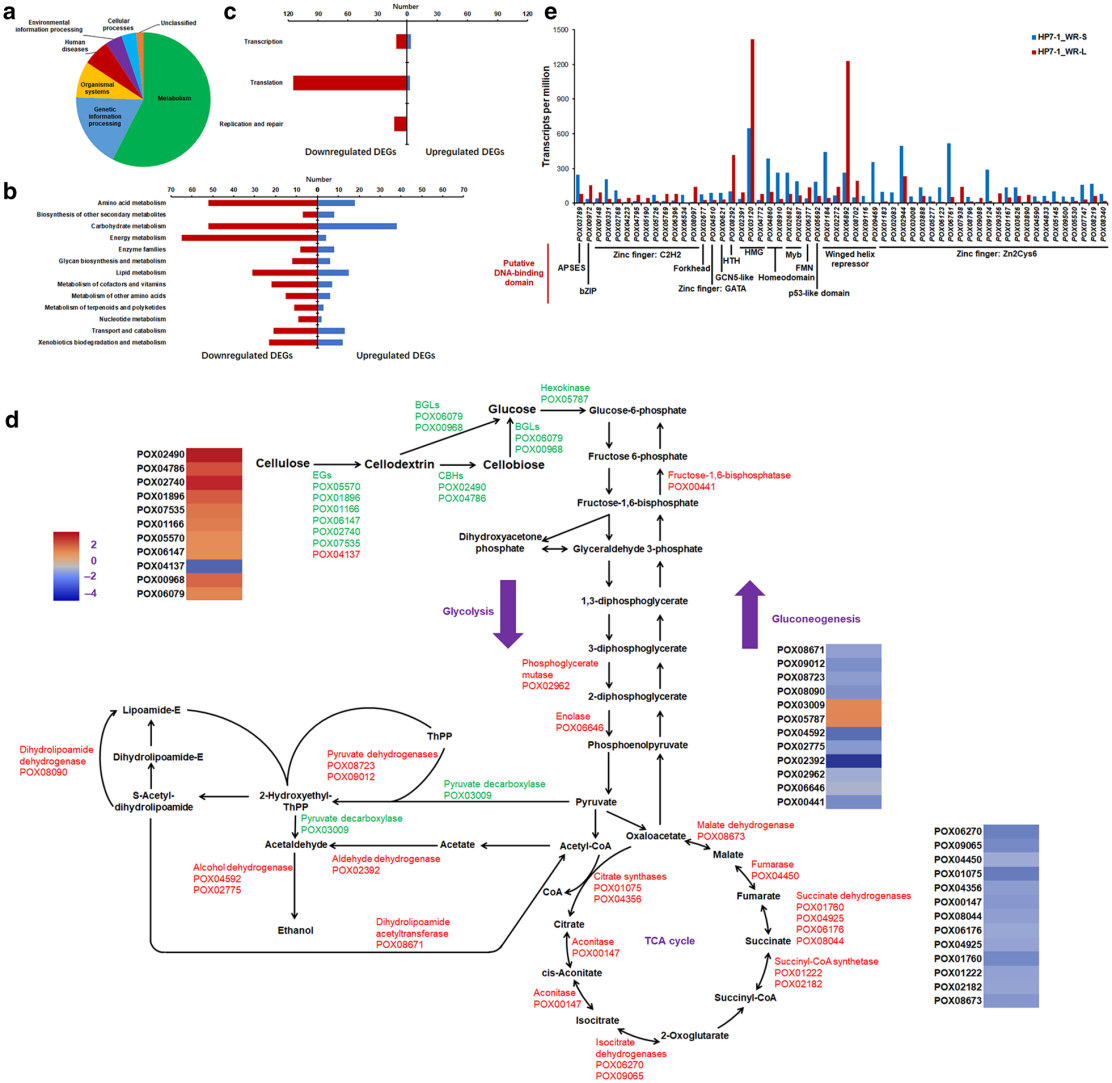
Comparative analysis of transcriptomes from *P. oxalicum* strain HP7-1 cultivated in media containing WR under SSF and SmF. **a** KEGG annotation of proteins encoded by DEGs in HP7-1_WR-S compared with HP7-1_WR-L. The screening criteria for DEGs were |log2-fold change| ≥ 0.8 and probability ≥ 1.0. **b** Number of upregulated and downregulated DEGs involved in metabolism in HP7-1_WR-S. **c** Number of upregulated and downregulated DEGs involved in genetic information processing in HP7-1_WR-S. **d** Transcriptional levels of DEGs involved in glycolysis/gluconeogenesis and the TCA cycle in HP7-1_WR-S. **e** Transcriptional levels of DEGs encoding putative TFs in HP7-1_WR-S. HP7-1_WR-S, *P. oxalicum* strain HP7-1 cultivated on solid medium containing WR under SSF; HP7-1_WR-L, *P. oxalicum* strain HP7-1 cultivated in liquid medium containing WR under SmF. WR, wheat bran plus rice straw; SSF, solid-state fermentation; SmF, submerged fermentation

Using |log2-fold change| ≥ 1 and probability ≥ 0.8 as thresholds, 1724 differentially expressed genes (DEGs) were identified by comparing *P*. *oxalicum* strain HP7-1 grown in solid medium containing WR (HP7-1_WR-S) and liquid medium containing WR (HP7-1_WR-L), comprising 772 upregulated (1.0 ≤ log2-fold change ≤ 12.9) and 952 downregulated (− 2.3 ≤ log2-fold change ≤ − 1.0) genes (Additional file 2: Table S2). Kyoto Encyclopedia of Genes and Genomes (KEGG) annotation analysis revealed that three quarters of the detected DEGs were involved in metabolism and genetic information processing (Fig. 1a). Among them, downregulated DEGs were more prevalent in almost all branches of metabolism and genetic information processing than upregulated DEGs, especially DEGs involved in translocation (Fig. 1b, c).

Among DEGs involved in glycolysis and gluconeogenesis, 12 were detected in the transcriptome of HP7-1_WR-S, including 10 downregulated with a log2-fold change of 1.0–5.0 and two upregulated with a log2-fold change of 1.2, compared with those in HP7-1_WR-L. These DEGs included hexokinase gene *POX05787*, fructose-1,6-bisphosphatase gene *POX00441*, phosphoglycerate mutase gene *POX02962*, enolase gene *POX06646*, pyruvate dehydrogenase genes *POX08723* and *POX09012*, pyruvate decarboxylase gene *POX03009*, aldehyde dehydrogenase gene *POX02392*, dihydrolipoamide dehydrogenase gene *POX08090*, alcohol dehydrogenase genes *POX04592* and *POX02775,* and dihydrolipoamide acetyltransferase gene *POX08671*. Moreover, 13 genes associated with the citric acid or tricarboxylic acid (TCA) cycle were identified, including citrate synthase genes *POX01075* and *POX04356*, aconitase gene *POX00147*, isocitrate dehydrogenase genes *POX06270* and *POX09065*, succinyl-CoA synthetase genes *POX01222* and *POX02182*, four succinate dehydrogenase genes (*POX01760*, *POX04925*, *POX06176,* and *POX08044*), fumarase gene *POX04450,* and malate dehydrogenase gene *POX08673,* all exhibiting a 2.3–4.5-fold decrease in transcript levels in HP7-1_WR-S compared with HP7-1_WR-L (Fig. 1d).

In general, glucose used for glycolysis in fungal cells is derived from exogenous carbon sources such as cellulose or starch. Exogenous carbon sources are degraded into glucose by carbohydrate-active enzymes (CAZymes), especially cellulases and amylases. Among the 1724 DEGs identified in HP7-1_WR-S, 158 encode putative CAZymes, including 106 upregulated and 52 downregulated genes with 1.1 ≤ log2-fold change ≤ 8.8 and − 7.2 ≤ log2-fold change ≤ − 1.1, respectively. Remarkably, 11 key cellulase genes, including two *cbh* genes *POX04786*/*Cel7A*-*1* and *POX02490*/*Cel6B*, seven, *eg* genes (*POX01166*/*Cel5B*, *POX01896*/*Cel5C*, *POX02740*, *POX04137*, *POX05570*/*Cel45A*, *POX06147*/*Cel5A,* and *POX07535*/*Cel12A*), and two β-glucosidase genes *POX00968* and *POX06079,* were identified among the 158 putative CAZyme genes. Except for *POX04137,* transcriptional levels of these cellulase genes were 2.1–11.4-fold higher in HP7-1_WR-S than in HP7-1_WR-L (Fig. 1d).

Comparative analysis of the transcriptomes of HP7-1_WR-S and HP7-1_WR-L also revealed significant differences in the transcription of 56 genes encoding putative TFs. Based on the DNA-binding domain predicted using Bioinformatics, these TFs could be classified into 12 types: zinc finger domain (Zn2Cys6, C2H2, GATA; 64%), winged helix repressor (11%), APSES domain (*A*SM-1, *P*hd1, *S*tuA, *E*FG1, and *S*ok2), bZIP, Forkhead, GCN5-like, high mobility group, helix–turn–helix (HTH), homeodomain, p53-like domain, FMN-binding split barrel, and Myb. In HP7-1_WR-S, transcripts of 36 of the 56 TF-encoding DEGs were upregulated between 1.1- and 315.1-fold compared with HP7-1_WR-L (Fig. 1e). It should be noted that 16 DEGs (*POX00331*/*FlbC*, *POX00972*/*ClrC*, *POX01184*, *POX01167*/*CxrA*, *POX02768*/*PacC*, *POX03789*/*StuA*, *POX03888*/*PrtT*, *POX03890*/*AmyR*, *POX04772*/*HmbB*, *POX04795*/*PDE_07134*, *POX04860*, *POX05692*/*Vib1*, *POX05726*, *POX08097*/*PDE_01706*, *POX08910,* and *POX09356*) are reportedly involved in cellulase production in filamentous fungi [9, 11–15].

### Novel regulatory genes potentially controlling cellulase production in *P. oxalicum* during SSF

**Fig. 2:**
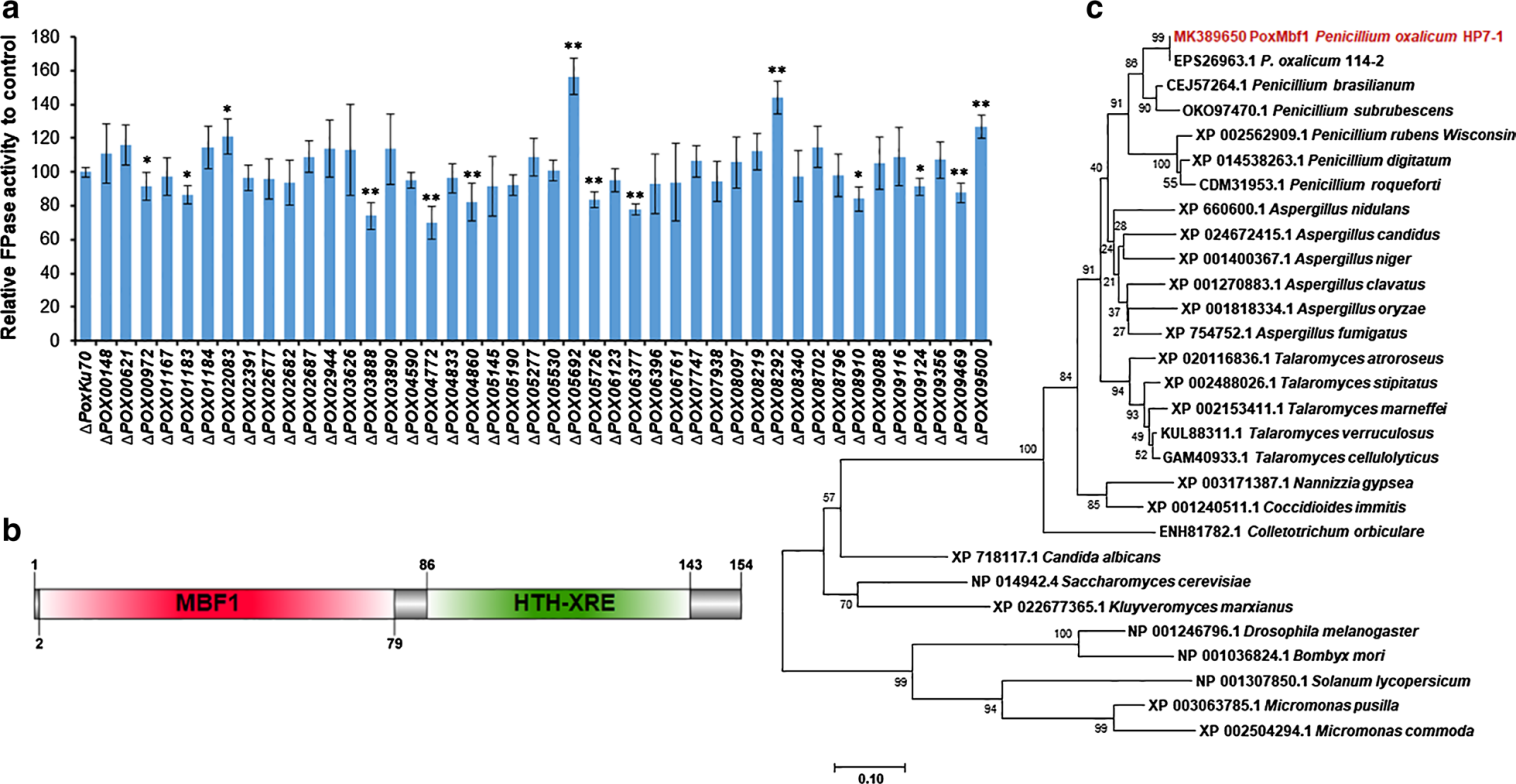
Screening and identification of novel regulatory genes involved in cellulase production in *P. oxalicum* during SSF. **a** FPase activities of deletion mutants of *P. oxalicum* strain ∆*PoxKu70* obtained through deletion of candidate regulatory genes during SSF using WR as the carbon source for 5 days after inoculation. *p ≤ 0.05 and **p ≤ 0.01 indicate significant differences between deletion mutants and parental strain ∆*PoxKu70* (Student’s *t* tests). **b** Analysis of conserved domains in the POX08292 protein. **c** Phylogenetic tree of POX08292 and its putative homologs. The tree was constructed based on the neighbour-joining method and Poisson model. Bootstrap values, derived from 1000 replicates, are shown at nodes. *WR* wheat bran plus rice straw, *SSF* solid-state fermentation

To identify the key TFs regulating cellulase gene expression in *P. oxalicum* during SSF, attempts were made to knock out 56 candidate TF-encoding genes in the ∆*PoxKu70* parental strain using homologous recombination (Additional file 3: Figure S1a). The resulting deletion mutants were confirmed by PCR using specific primer pairs (Additional file 4: Table S3), and 43 were successfully generated (Additional file 3: Figure S1b; Additional file 5: Table S4), including 18 mutants constructed in the previous studies [9, 16]. Enzyme activity assays revealed significant alterations in filter paper cellulase (FPase) production in 14 mutants (∆*POX00972*, ∆*POX01183*, ∆*POX02083*, ∆*POX03888*, ∆*POX04772*, ∆*POX04860*, ∆*POX05692*, ∆*POX05726*, ∆*POX06377*, ∆*POX08292*, ∆*POX08910*, ∆*POX09124*, ∆*POX09469,* and ∆*POX09500*) during SSF using WR as the carbon source for 5 days compared with ∆*PoxKu70* (*p* ≤ 0.05, Student’s *t* test; Fig. 2a). To the best of our knowledge, the present study is the first to confirm the involvement of these genes in cellulase production in *P*. *oxalicum* during SSF (Table 1). Intriguingly, to date, five of the 14 genes (*POX02083*, *POX08292*, *POX09124*, *POX09469,* and *POX09500*) have not been reported previously to be associated with cellulase production in *P*. *oxalicum* during SSF or SmF. In the present study, ∆*POX08292* exhibited the highest FPase production (44.3% increase) among the five deletion mutants (∆*POX02083*, ∆*POX08292*, ∆*POX09124*, ∆*POX09469,* and ∆*POX09500*) compared with ∆*PoxKu70* (Fig. 2a). Hence, *POX08292* was selected for further investigation of its regulatory roles. To exclude the possibility of multiple copies of the *POX08292* deletion cassette being integrated into the genome of ∆*PoxKu70*, Southern hybridisation was performed using a specific probe (Additional file 4: Table S3). The appearance of the expected bands on the gel (Additional file 3: Figure S1c) suggests that only one copy of the *POX08292* knockout cassette was inserted into the genome of ∆*PoxKu70*.

**Table 1:** Novel regulatory genes potentially regulating cellulase production in *P*. *oxalicum* during SSF

Gene ID	GenBank accession number	InterPro annotation^a^	Domain description	Known homologous TFs under SmF^b^	Identity (%)
*POX00972*	MK389644	IPR004827	Basic leucine zipper (bZIP)	ClrC	100
*POX01183*	MK389645	IPR001138	Zinc finger: Zn2Cys6 type	NA	NA
*POX02083*	MK389646	IPR001138	Zinc finger: Zn2Cys6 type	NA	NA
*POX03888*	MK389647	IPR001138	Zinc finger: Zn2Cys6 type	PrtT	100
*POX04772*	KY860736	IPR009071	High mobility group box	HmbB	41
*POX04860*	KY922971	IPR009057	Homeodomain-like	PDE_07199	99
*POX05692*	MK389648	IPR008967	p53-like	Vib1	NA
*POX05726*	KY860737	IPR007087	Zinc finger: C2H2 type	NA	NA
*POX06377*	MK389649	IPR007396	PAI2-type	PAIB	27
*POX08292*	MK389650	IPR001387	Helix–turn–helix type 3	Mbf1p	51
*POX08910*	KY860739	IPR009057 IPR007526	Homeodomain-like; SWIRM domain	NA	NA
*POX09124*	MK389651	IPR001138	Zinc finger: Zn2Cys6 type	NA	NA
*POX09469*	MK389652	IPR011991	Winged helix repressor DNA-binding domain	NA	NA
*POX09500*	MK389653	IPR001138 IPR007219	Zinc finger, Zn2Cys6 type; Fungal_Trans	NA	NA

^a^IPR, InterPro database (http://www.ebi.ac.uk/interpro/scan.html)

^b^TF, transcription factor: ClrC and PrtT from *P*. *oxalicum* 114-2 (EPS34061.1 and EPS29021.1); Vib1 from *Neurospora crassa* OR74A (XP_011394570.1); PAIB from *Saccharomyces cerevisiae* 131 (ONH80768.1); HmbB from *A. nidulans* FGSC A4 (XP_658871); Mbf1p from *S. cerevisiae* S288C (NP_014942.4)

### Sequence analysis of POX08292

Simple Modular Architecture Research Tool (SMART) analysis revealed that the POX08292 protein, comprising 154 amino acids, contains an MBF1 domain (PF08523, *E* value = 5.9e−29) and an HTH XRE-family domain (SM000530, *E* value = 1.7e−08; http://smart.embl-heidelberg.de/; Fig. 2b). Protein alignment using NCBI BLASTP indicated that POX08292 shares 100%, 82%, and 51% identity with PDE_01903 in *P*. *oxalicum* 114-2 (GenBank accession number EPS26963.1), AN2996.2 in *A. nidulans* FGSC A4 (XP_660600.1), and Mbf1p in *S. cerevisiae* S288C (NP_014942.4), respectively. Furthermore, POX08292 was found to be evolutionarily close to orthologs in *Aspergillus* and *Talaromyces* (Fig. 2c). To facilitate subsequent study, POX08292 was re-designed as PoxMBF1 (where Pox represents *P. oxalicum*).

### Regulation of cellulase and xylanase production in *P*. *oxalicum* by PoxMBF1 during SSF and SmF following induction

**Fig. 3:**
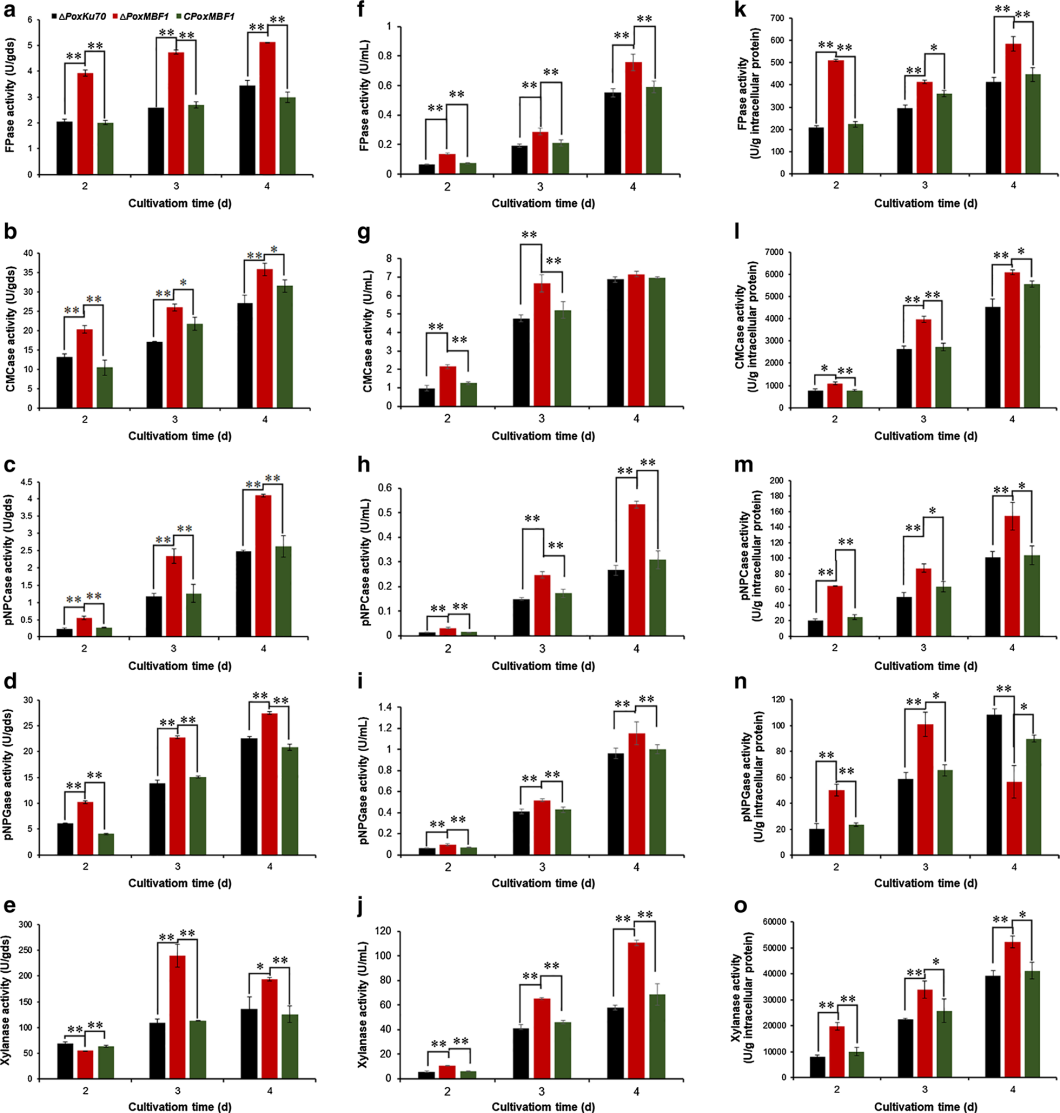
Cellulase and xylanase production in *P. oxalicum* deletion mutant ∆*PoxMBF1*, parental strain ∆*PoxKu70,* and complementary strain C*PoxMBF1* cultivated in solid medium containing WR (**a**–**e**) and liquid medium containing WR (**f**–**j**) or Avicel (**k**–**o**) after a shift from glucose. Enzymatic activity was determined at 2–4 days after transfer from glucose. *p ≤ 0.05 and **p ≤ 0.01 indicate significant differences between deletion mutants and parental strain ∆*PoxKu70* or complementary strain C*PoxMBF1* (Student’s *t* tests)

To further investigate the regulatory roles of *PoxMBF1* in detail, *P*. *oxalicum* strain ∆*PoxMBF1* (∆*POX08292*) and the parental strain ∆*PoxKu70* were cultivated under different fermentation conditions (SSF and SmF) for 2–4 days after transfer from glucose. Enzyme activity tests revealed a 21.6–131.4% increase in cellulase activity corresponding to FPase, carboxymethylcellulase (CMCase), *p*-nitrophenyl-β-cellobiosidase (pNPCase), and *p*-nitrophenyl-β-glucopyranosidase (pNPGase), as well as xylanase production, by ∆*PoxMBF1* during SSF using WR as the carbon source (except for xylanase production at 2 days) compared with ∆*PoxKu70* (Fig. 3a–e).

Cultivation of *P*. *oxalicum* strains ∆*PoxMBF1* and ∆*PoxKu70* in liquid medium containing WR under SmF for 2–4 days after a shift from glucose resulted in a 20.0–122.6% increase in cellulase and xylanase production by ∆*PoxMBF1* compared with ∆*PoxKu70*, except for CMCase production at 4 days (Fig. 3f–j). Similar results were observed when cultured in liquid medium containing Avicel, and *PoxMBF1* deletion resulted in a ~ 34.0–217.3% increase in cellulase production by *P*. *oxalicum*, except for pNPGase production at 4 days (Fig. 3k–o).

Furthermore, to confirm that the increase in cellulase and xylanase production by ∆*PoxMBF1* was the result of *PoxMBF1* deletion, complementary strain C*PoxMBF1* was constructed and confirmed by PCR with specific primers (Additional file 4: Table S3; Additional file 6: Figure S2). The FPase, CMCase, pNPCase, pNPGase, and xylanase cellulase yields from complementary strain C*PoxMBF1* were not significantly different from those of ∆*PoxKu70* during SSF or SmF (Fig. 3).

### Effects of PoxMBF1 on growth and extracellular stress adaptation in *P. oxalicum*

To investigate the effects of *PoxMBF1* on the growth of *P*. *oxalicum*, the ∆*PoxMBF1* deletion mutant and ∆*PoxKu70* parental strain were inoculated into liquid medium containing either glucose or Avicel as the carbon source. Neither ∆*PoxMBF1* nor ∆*PoxKu70* displayed significant differences in growth (Additional file 7: Figure S3).

**Fig. 4:**
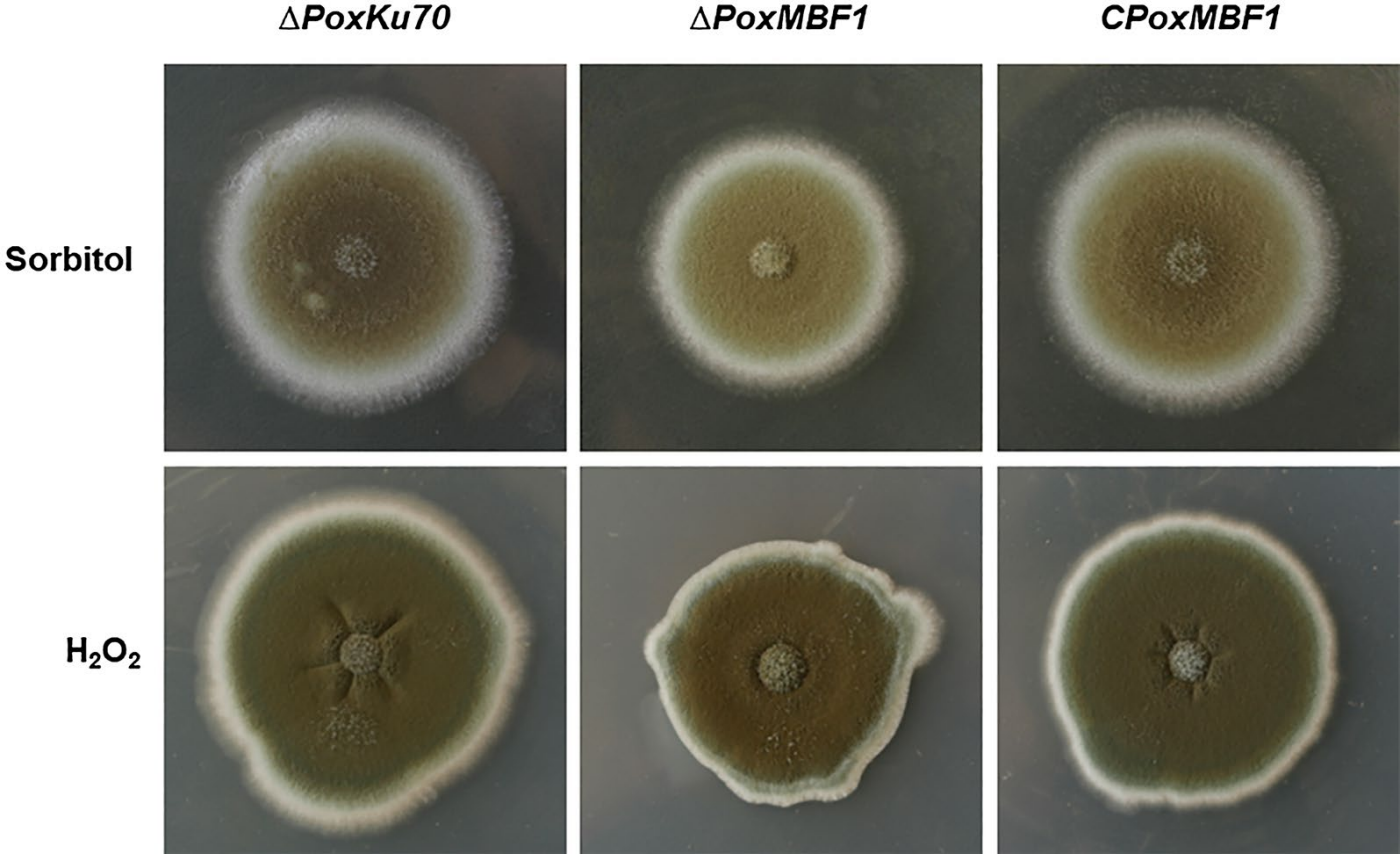
Phenotypic investigation of *P. oxalicum* deletion mutant ∆*PoxMBF1*, parental strain ∆*PoxKu70,* and complementary strain C*PoxMBF1* cultivated under extracellular stress

To determine whether *PoxMBF1* was involved in the responses of *P*. *oxalicum* to extracellular stress, strains ∆*PoxMBF1*, ∆*PoxKu70,* and C*PoxMBF1* were inoculated onto potato dextrose agar (PDA) plates containing 1.5 M sorbitol or 1.8 mM H_2_O_2_. The results revealed smaller colonies for the ∆*PoxMBF1* mutant than ∆*PoxKu70* and C*PoxMBF1*, and colonies of ∆*PoxKu70* and C*PoxMBF1* were similar (Fig. 4), suggesting that *PoxMBF1* deletion resulted in high sensitivity of *P*. *oxalicum* to exogenous stress.

### Transcriptional regulation of cellulase and xylanase genes in *P*. *oxalicum* by PoxMBF1 during SSF and SmF

PoxMBF1 was found to be involved in cellulase and xylanase production in *P*. *oxalicum*, possibly due to alterations in cellulase and xylanase genes at the mRNA level. To explore this further, real-time quantitative reverse-transcription PCR (RT-qPCR) was performed on *cbh* gene *POX05587*/*Cel7A*-*2*, *eg* genes *POX01166*/*Cel5B*, *POX02740* and *POX05571*/*Cel7B*, β-glucosidase gene *POX06835*/*Bgl1*, and *xyn* genes *POX06783*/*Xyn11A* and *POX08484*/*Xyn11B*. The transcriptional levels of these genes were measured in *P*. *oxalicum* strains ∆*PoxMBF1* and ∆*PoxKu70* cultivated under different fermentation conditions at 12, 24, and 48 h after a shift from glucose.

**Fig. 5:**
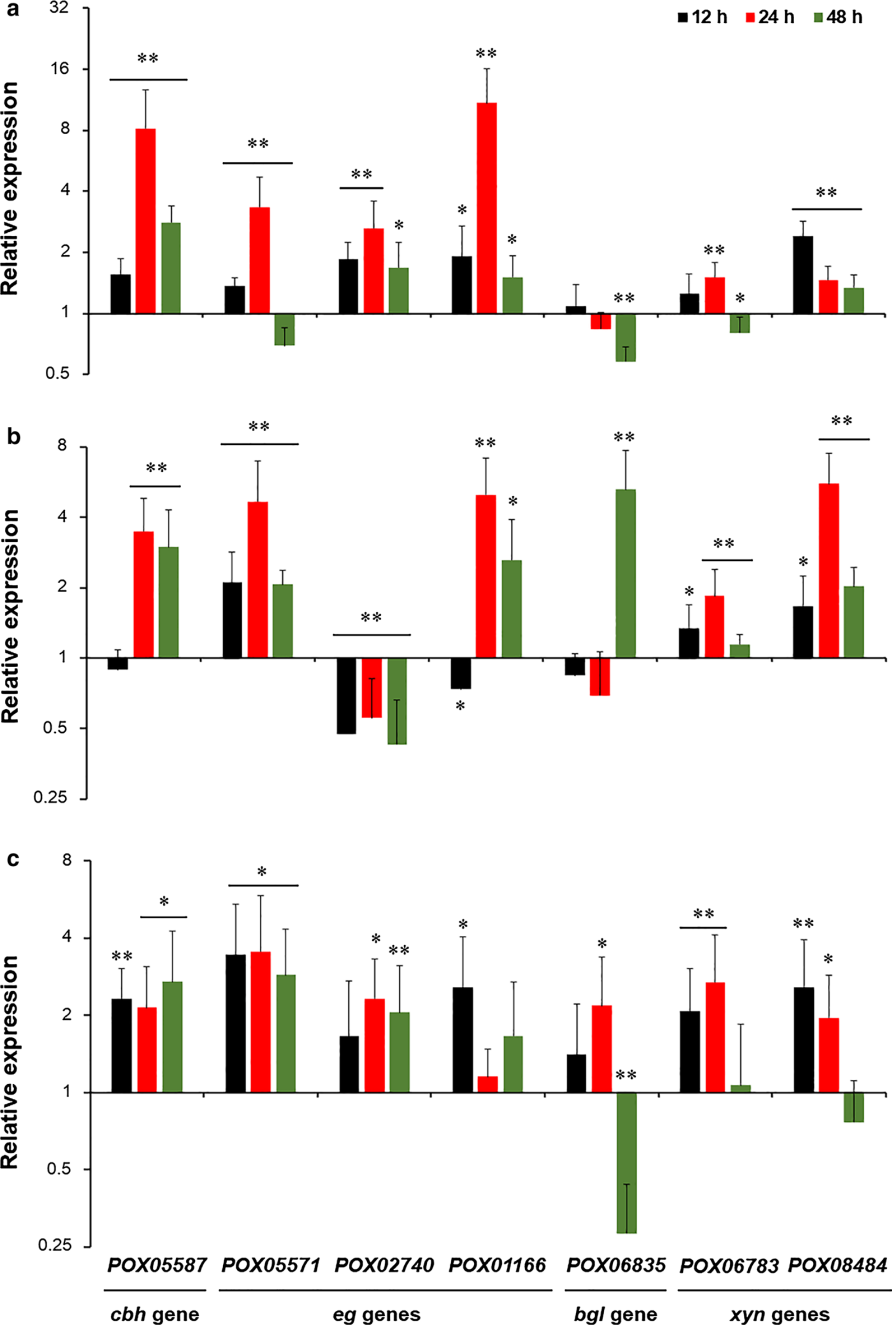
Transcriptional levels of major cellulase and xylanase genes regulated by *PoxMBF1* in *P. oxalicum* at three timepoints (12, 24, and 48 h) after a shift from glucose. **a** Solid medium containing WR; **b** liquid medium containing WR; **c** liquid medium containing Avicel. All gene expression levels were normalised against the parental strain ∆*PoxKu70*. ** or * indicates significant differences (*p* ≤ 0.01 or *p* ≤ 0.05, respectively) between the deletion mutant ∆*PoxMBF1* and parental strain ∆*PoxKu70* (Student’s *t* tests). WR, wheat bran plus rice straw

In ∆*PoxMBF1* under SSF with WR as the carbon source, transcripts of *POX05587*/*Cel7A*-*2*, *POX02740*, *POX01166*/*Cel5B,* and *POX08484*/*Xyn11B* were increased by 33.8–718.3% compared with ∆*PoxKu70* during the entire induction period (12–48 h), whereas *POX05571*/*Cel7B* and *POX06783*/*Xyn11A* transcripts were increased only at 12–24 h or 24 h. Strikingly, transcripts of *POX05571*/*Cel7B*, *POX06783*/*Xyn11A* and *POX06835*/*Bgl1* were decreased by 19.6–42.1% at 48 h (Fig. 5a). In addition, expression of these cellulase and xylanase genes was also observed in ∆*PoxMBF1* and ∆*PoxKu70* during SmF using either WR or Avicel as the carbon source. These results indicate that the expression of most of the tested cellulase and xylanase genes was significantly increased in ∆*PoxMBF1* compared with ∆*PoxKu70* at certain induction periods, although the extent varied with different carbon sources used for induction. For example, transcription of *POX05587*/*Cel7A*-*2* was increased by 132.6–270.7% after 12–48 h of induction with Avicel, and by 198.7% and 248.5% at 24 and 48 h of induction with WR, respectively (Fig. 5b, c).

### In vitro binding of PoxMBF1

**Fig. 6:**
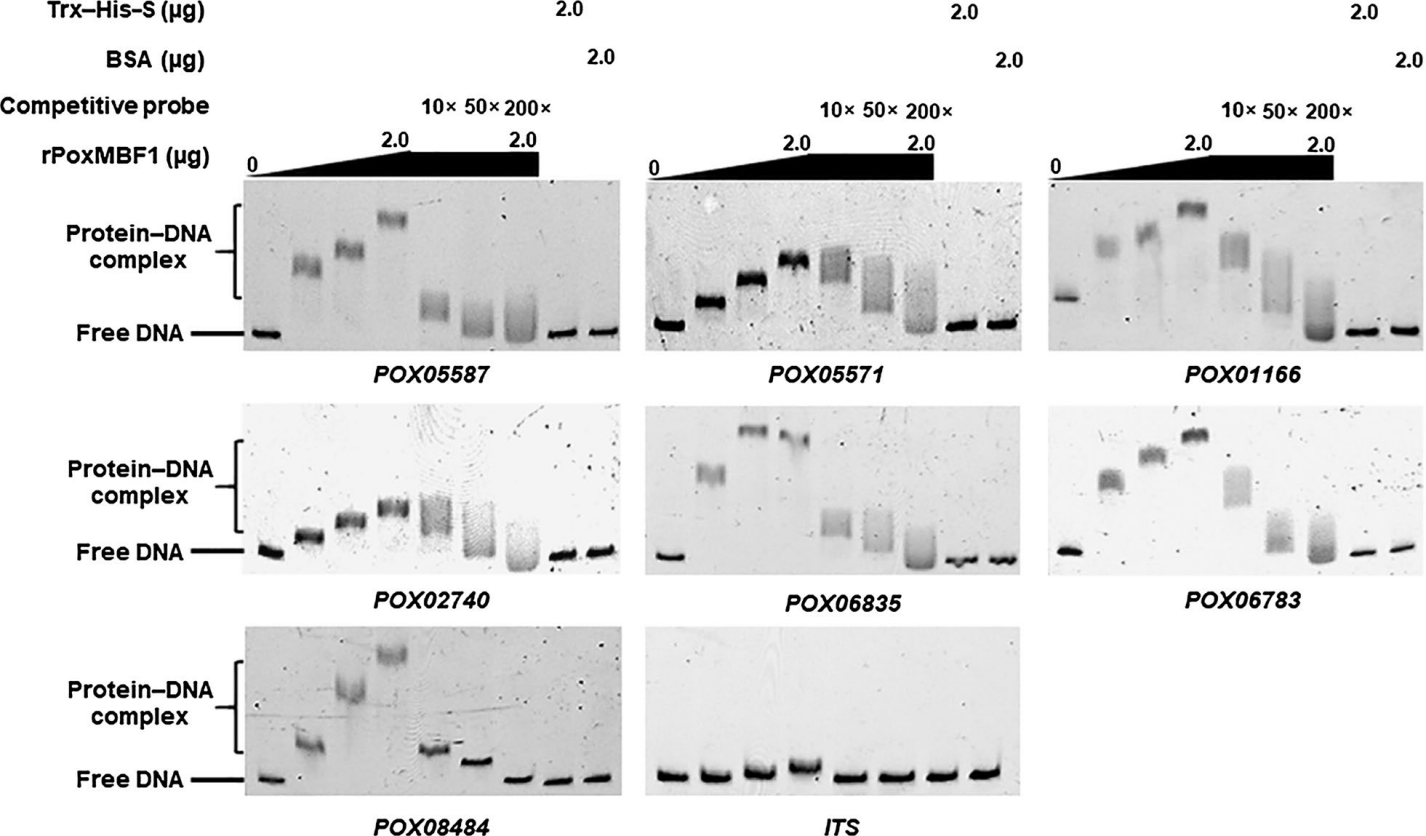
In vitro binding experiments using recombinant protein rPoxMBF1 and the promoter regions of target genes. Each EMSA reaction system contained 0–2.0 μg of Trx–His–S-tagged rPoxMBF1 and ~ 40 ng of each candidate probe. The same amounts of purified Trx–His–S fusion protein, BSA, and ITS sequence were, respectively, employed as negative controls

The above results revealed that PoxMBF1 regulates cellulase and xylanase production in *P*. *oxalicum* by controlling transcription of cellulase and xylanase genes. To determine whether PoxMBF1 directly or indirectly controls the expression of these genes, in vitro binding experiments were performed. Electrophoretic mobility shift assay (EMSA) results indicated that the rPoxMBF1–DNA complex was formed with each probe, and the concentration and size of each complex gradually increased with increasing quantity of rPoxMBF1 (0–2.0 µg). By contrast, rPoxMBF1 did not bind the fusion protein Trx–His–S, bovine serum albumin (BSA), or the internal transcribed spacer (ITS) sequence controls (Fig. 6). Moreover, when a mixture of 6-carboxyfluorescein (6-FAM)-labelled EMSA probes and competitive probes lacking 6-FAM were loaded into buffer containing a certain amount of rPoxMBF1, a gradual decrease was observed in the concentration and size of complexes with increasing amounts of competitive probes. These results suggest that PoxMBF1 binds specifically to the putative promoters of major cellulase and xylanase genes in *P*. *oxalicum* (Fig. 6).

## Discussion

In the present study, transcriptional profiling was performed on the soil fungus *P*. *oxalicum* cultivated under different fermentation conditions (SSF and SmF), and transcription of genes involved in metabolism and genetic information processing was found to vary. Intriguingly, compared with WR-based SmF, WR-based SSF induced higher expression of major cellulase genes in *P*. *oxalicum,* but repressed the transcription of genes involved in the TCA cycle, suggesting that SSF is favourable for fungal cellulase production. Indeed, cellulase and xylanase production was greater in SSF than SmF during both early and intermediate induction periods (Fig. 3). These findings demonstrate the advantages of SSF for cellulase production at the molecular level.

Comparative transcriptomic and genetic analyses identified 14 genes putatively regulating cellulase (FPase) production in *P*. *oxalicum* during SSF. However, the roles of these regulatory genes varied; four genes (*POX02083*, *POX05692*, *POX08292*/*PoxMBF1,* and *POX09500*) negatively regulated FPase production, while the others positively regulated FPase production. Some of these genes were found to function only during SSF (*POX01183*, *POX03888,* and *POX06377*), SmF (*POX01167* and *POX03890*), or both (*PoxMBF1*, *POX04772,* and *POX08910*) [9, 15], suggesting the presence of distinct regulatory networks that differ in SSF and SmF. Furthermore, detailed investigation of the regulatory role of the novel key TF PoxMBF1 in cellulase and xylanase production in *P*. *oxalicum* during SSF and SmF revealed that PoxMBF1 functions by binding directly to the promoter regions of major cellulase and xylanase genes. Thus, PoxMBF1 could be a potential direct target for genetic engineering to improve cellulase and xylanase yields.

The transcriptional co-activator MBF1 is present in a wide variety of organisms including archaea, humans, plants, and filamentous fungi, and plays significant roles in regulating diverse cellular processes. For example, in the plant *Arabidopsis thaliana*, MBF1 controls development and environmental stress tolerance [17, 18]. Human MBF1 functions in the differentiation of endothelial cells [19]. In *Magnaporthe oryzae*, MBF1 is involved in vegetative growth, osmotic stress, and virulence [20]. In the present study, MBF1 was found to directly control the expression of cellulase and xylanase genes in the filamentous fungus *P*. *oxalicum*.

Notably, MBF1 mediates transcriptional regulation via bridging specific regulatory factors and TATA-box binding protein (TBP) [21]. In insects such as the silkworm *Bombyx mori* and *Drosophila*, MBF1 is a transcriptional cofactor that links TBP and the nuclear hormone receptor FTZ-F1 by stimulating FTZ-F1 binding to its recognition site. FTZ-F1 is involved in the activation of the *fushi tarazu* gene during embryogenesis [21]. Yeast MBF1 mediates the GCN4-dependent transcriptional activation of the *HIS3* gene-encoding imidazole-3-phosphate dehydratase by directly binding to TBP and the DNA-binding domain of GCN4 [22]. However, remarkably, no interaction between MBF1 and GCN4 (Cpc1) was detected in *M*. *oryzae* [20] or *Fusarium fujikuroi* [23], suggesting that the biological functions of MBF1 are diverse and dependent on host cells. In addition, human EDF-1, a homolog of MBF1, can bind to calmodulin, which is mediated by Ca^2+^ concentration and phosphorylation of EDF-1 by protein kinase C [24].

In addition to the MBF1 domain (PF08523), the MBF1 protein includes an HTH XRE-family domain (SM000530), also known as a Cro/C1-type HTH domain (IPR001387), located in the C-terminus, that is annotated as a DNA-binding domain of transcriptional regulators. The HTH XRE domain contains four α-helices, and is responsible for MBF1 function [25–27]. In the present study, purified recombinant rPoxMBF1 protein could directly bind to the promoter regions of major cellulase and xylanase genes in vitro, thereby controlling their transcriptional levels in *P*. *oxalicum*. Remarkably, the regulatory role of PoxMBF1 in cellulase and xylanase production in *P*. *oxalicum* tended to diminish as fermentation proceeded in both SSF and SmF, and the extent of PoxMBF1 regulation was higher in SSF than SmF. In addition, levels of cellulase and xylanase transcripts varied due to differences in PoxMBF1 regulation under different induction conditions. These findings suggest that PoxMBF1 function is dependent on regulatory networks and host specificity. Nevertheless, further studies are needed to investigate the exact regulatory roles of PoxMBF1 in cellulase and xylanase production in *P*. *oxalicum*.

## Conclusions

In this study, we revealed differences in transcriptomic regulation in *P*. *oxalicum* cultivated under SSF and SmF; WR_SSF induced higher expression of major cellulase genes than WR_SmF, but repressed the transcription of genes involved in the citric acid cycle. A *PoxMFB1* null mutant displayed significantly higher cellulase and xylanase production than the parental strain in both SSF and SmF, accompanied by an increase in transcription of a subset of cellulase and xylanase genes via specific binding to their putative promoters. These findings expand our understanding of the regulatory mechanisms of fungal cellulase and xylanase genes during SSF, and could assist genetic engineering of fungal strains with improved cellulase and xylanase yields.

## Methods

### Microbial strains and media

Fungal strains were maintained on PDA plates at 4 °C for passage. Fungal spores were generated and collected from the PDA plates and incubated for 6 days at 28 °C. In general, fungal spores were resuspended in 0.1% Tween 80, and their concentration was adjusted to 1 × 10^8^/mL.

For enzyme activity assays and RT-qPCR, *P*. *oxalicum* strains were directly inoculated onto solid medium containing WR as the carbon source as described previously [4] and cultured for 5 days at 28 °C. WR medium contained 6.0 g of wheat bran, 4.0 g of rice straw, and 20 mL of mineral salt solution (2.5 g/L KH_2_PO_4_, 2.5 g/L MgSO_4_·7H_2_O, 5.0 g/L yeast extract, 0.1% Tween 80, 5.0 mg/mL FeSO_4_·7H_2_O, 1.6 mg/L MnSO_4_·H_2_O, 1.4 mg/L ZnSO_4_·7H_2_O, and 2.0 mg/L CoCl_2_; pH 5.0). Raw wheat bran and rice straw were purchased from a local farmer’s market (Nanning, China). All plant materials were dried in an oven at 50 °C overnight, and then milled to 40-mesh particle size for further study. For shift experiments, *P*. *oxalicum* spores (1.0 × 10^8^) were precultured in glucose medium comprising 4 g/L (NH_4_)_2_SO_4_, 4 g/L KH_2_PO_4_, 0.6 g/L CaCl_2_, 0.6 g/L MgSO_4_·7H_2_O, 0.005 g/L FeSO_4_·7H_2_O, 0.0016 g/L MnSO_4_, 0.0017 g/L ZnCl_2_, 0.002 g/L CoCl_2_, 10 g/L glucose, and 1 mL of Tween 80 for 20 h at 28 °C. Subsequently, a defined amount of precultured mycelia was transferred to fresh solid or liquid WR medium and incubated for 2–4 days at 28 °C (for enzyme activity assays) or 12, 24, and 48 h (for RT-qPCR). Crude cellulase solution was extracted from solid and liquid media according to a previous method [4] and by centrifugation, respectively. The resulting mycelia were used for the extraction of total RNA for RT-qPCR or RNA sequencing.

To perform phenotypic investigation, a defined number of fungal spores were inoculated onto PDA plates containing different carbon sources (glucose, WR or Avicel) or chemical agents (H_2_O_2_ or sorbitol) for 3–5 days. For RNA sequencing of *P*. *oxalicum* strain HP7-1 obtained from the China General Microbiological Culture Collection (CGMCC) 10781, 1 mL of spore suspension at a concentration of 1.0 × 10^8^/mL was inoculated into solid or liquid WR medium and incubated at 28 °C for 24 h under static or shaking conditions, and mycelia were used for total RNA extraction. *Escherichia coli* cells (TransGen, Beijing, China) were cultivated in Luria–Bertani medium at 37 °C and used for genetic engineering.

### Total DNA and RNA extraction

Extraction of total DNA and RNA from *P*. *oxalicum* was performed as described previously [3]. The collected mycelia were mechanically ground into powder with liquid nitrogen, mixed with lysate buffer (10.0 mM ethylenediaminetetraacetic acid, 20.0 mM sodium acetate, 1.0% sodium dodecyl sulfate, and 40.0 mM TRIS–HCl; pH 8.0), and used for extracting fungal total DNA. For total RNA extraction, a TRIzol RNA Kit (Life Technologies, Carlsbad, CA, USA) was employed according to the manufacturer’s instructions. Electrophoresis on 1% agarose gels and A_260_/A_280_ values were used to assess the quantity and quality of extracted DNA and RNA.

### RNA sequencing

Total RNA extracted from fungal mycelia cultivated for 24 h was employed for RNA sequencing using an Illumina HiSeq 2000 system. As reported previously [3], a cDNA library was constructed and evaluated with an ABI StepOnePlus Real-Time PCR System (Applied Biosystems, Foster City, CA, USA) and an Agilent 2100 Bioanalyzer (Agilent Technologies, Santa Clara, CA, USA). Each cDNA fragment in the constructed cDNA library was 100 bp in length. After filtering, the obtained clean reads were mapped onto the *P*. *oxalicum* HP7-1 genome for functional annotation using BWA v0.7.10-r789 [28] and Bowtie2 v2.1.0 [29]. Levels of gene transcripts were calculated as fragments per kilobase of exon per million mapped reads (FPKM) values using RSEM v1.2.12 [30]. DEGs were screened and selected by comparative analysis using NOISeq [31].

### Construction of gene deletion mutant and complementary strains

Deletion mutants of candidate regulatory genes and complementary strain C*PoxMBF1* were constructed using *P*. *oxalicum* parental strains ∆*PoxKu70* and ∆*PoxMBF1*, respectively, by homologous recombination according to previously described methods [9]. Briefly, ∆*PoxKu70* protoplasts were prepared using OM solution comprising 1.2 mM MgSO_4_·7H_2_O, 10 mM NaH_2_PO_4_, 4 g/L lysozyme, 6 g/L lysing enzymes from *Trichoderma harzianum* (Sigma-Aldrich, St. Louis, MO, USA), and 6 g/L snailase (pH 5.8). Knockout cassettes for each candidate gene, comprising ~ 2 kb of DNA sequence upstream and downstream of the target gene and a 1.8 kb DNA fragment encoding the G418 resistance gene, were constructed by recombinant PCR. Subsequently, ~ 2 µg of each knockout cassette was mixed with a defined number of ∆*PoxKu70* protoplasts on ice and transferred to plates containing OCM medium comprising 1 g/L casein enzymatic hydrolysate, 1 g/L yeast extract, and 10 g/L agar, and incubated at 50 °C for 30 min. PDA medium containing 250 μg/mL hygromycin B and 500 μg/mL G418 was then poured onto the surface of the OCM medium and incubated for 5 days at 28 °C, and transformants were selected and confirmed.

To further verify that only the *PoxMBF1* gene was deleted in the ∆*PoxMBF1* mutant compared with the parental strain ∆*PoxKu70* and that the phenotypic alteration of ∆*PoxMBF1* was due to deletion of *PoxMBF1* in ∆*PoxKu70*, a complementary strain was constructed. A complementary cassette was integrated at the site of aspartic protease gene *POX05007* in deletion mutant ∆*PoxMBF1*, and included ~ 2 kb of DNA sequence upstream and downstream of *POX05007*, a 1.2 kb DNA fragment encoding the bleomycin resistance gene, and a 4.7 kb complete *PoxMBF1* gene containing its promoter, coding region, and terminator. The complementary cassette was introduced into ∆*PoxMBF1* protoplasts, and bleomycin was used for selection of complementary transformants.

### Enzyme activity assays

Cellulase and xylanase activities of *P*. *oxalicum* strains were determined as described previously [3]. Appropriately diluted crude enzyme solution was added to 100 mM citrate buffer (pH 5.0) containing Whatman No. 1 filter paper (50 mg, 1.0 × 6.0 cm^2^), 1.0% CMC-Na (Sigma-Aldrich, Darmstadt, Germany), and 1.0% xylan from beechwood (Megazyme International Ireland, Wicklow, Ireland) to measure the activities of FPase, CMCase, and xylanase. Assays were incubated for 1 h, 30 min, or 10 min at 50 °C. The concentration of the resulting reducing sugars was determined using DNS reagent comprising 200 g/L potassium sodium tartrate, 0.5 g/L Na_2_SO_3_, 10 g/L 3,5-dinitrosalicyclic acid, 20 g/L NaOH, and 2 g/L phenol at 540 nm [32]. One unit (U) of enzyme activity was defined as the amount of enzyme required to produce 1 μmol of reducing sugar per min from the substrate.

Enzyme activities of pNPCase and pNPGase were determined using *p*-nitrophenyl-β-d-cellobioside and *p*-nitrophenyl-β-d-glucopyranoside (Sigma-Aldrich) as substrate, respectively, at 50 °C for 15 min, and the resulting *p*-nitrophenol (pNP) was measured at 410 nm. One unit (U) of enzyme activity was defined as the amount of enzyme required to produce 1 μmol of pNP per min from the substrates. Furthermore, a Detergent Compatible Bradford Assay Kit (Pierce Biotechnology, Rockford, IL, USA) was employed to determine the protein concentration in fungal cells according to the manufacturer’s instructions.

### RT-qPCR assays

RT-qPCR was performed as reported previously [3]. Briefly, first-stand cDNA was synthesised using the extracted total fungal RNA as template by employing a PrimeScript RT Reagent Kit (TaKaRa Bio Inc, Dalian, China). Subsequently, the tested DNA fragments were amplified by PCR using the synthesised cDNA as template in a 20 µL reaction containing 0.8 μL of 10 μM primers (Additional file 4: Table S3), 0.2 μL of first-stand cDNA, and 10 μL of SYBR Premix Ex Taq II (TaKaRa Bio Inc.). All reactions were run for 40 cycles at 95 °C for 5 s and 60 °C for 40 s, and fluorescent signals were determined at the end of the extension step at 80 °C. Relative gene expression levels in the Δ*PoxMBF1* deletion mutant were calculated using the actin gene *POX09428* as an internal control, and normalised against the Δ*PoxKu70* parental strain. Each RT-qPCR experiment was performed independently at least three times.

### Heterologous expression of *PoxMBF1*

A DNA fragment encoding the ProMBF1 protein was amplified by PCR with a specific primer pair (Additional file 4: Table S3) and cloned into the pET-32a(+) vector to generate recombinant plasmid pET32-PoxMBF1. This construct was introduced into *E*. *coli* Rosetta cells (Transgen Biotech, Beijing, China), and the resulting recombinant strain was first cultured in Luria–Bertani medium for 4 h and then induced with 1.0 mM isopropyl-β-d-thiogalactopyranoside at 25 °C for 20 h to produce the fusion protein possessing a TRX–His–S tag. The recombinant rPoxMBF1 protein was purified by affinity chromatography on TALON Metal Affinity Resin (Clontech, Palo Alto, CA, USA). TRX–His–S was purified from *E*. *coli* cells harbouring the empty pET-32a(+) vector and used as a control.

### In vitro binding experiments

To investigate whether the recombinant rPoxMBF1 protein directly binds to the promoter regions of major cellulase and xylanase genes, in vitro EMSA was performed as described previously [9]. DNA fragments (~ 1 kb upstream from the translation initiation ATG codon) labelled with 6-FAM at the 3′-terminus were amplified by PCR using specific primer pairs (Additional file 4: Table S3) and used as EMSA probes. The rPoxMBF1 and EMSA probes were mixed with binding buffer comprising 0.1 mg/mL BSA, 20 mM TRIS–HCl (pH 8.0), 5% glycerol, 50 mM KCl, 1 mM DTT, and 5.4 μg of sheared salmon sperm DNA, at room temperature for 20 min. The mixture was loaded onto a polyacrylamide TRIS–acetic acid–EDTA gel and run at 150 V for 77 min. The resulting protein–DNA complexes were investigated using a ChemiDoc MP imaging system (Bio-Rad Laboratories, Hercules, CA, USA). Competitive EMSA was performed as described above using EMSA probes without 6-FAM. Purified TRX–His–S from *E. coli* cells transformed with the empty pET-32a(+) vector and BSA alone were used as controls, along with the control probe ITS sequence.

### Phylogenetic analysis

The PoxMBF1 protein and its homologs (downloaded from the NCBI website) were subjected to phylogenetic analysis using MEGA7.0 [33]. A phylogenetic tree was constructed based on the neighbour-joining method and Poisson correction model. Bootstrap values were calculated from 1000 replicates.

### Data analysis and accession numbers

Experimental data were statistically analysed using Student’s *t* tests in Microsoft Excel within Microsoft Office 2016 (Microsoft, Redmond, WA, USA). Gene expression data and DNA sequences have been deposited in the Sequence Read Archive database (accession numbers SRR8392062–SRR8392067) and the GenBank database (accession numbers MK389644–MK389653), respectively.

## Additional files

**Additional file 1: Table S1.** Summary of RNA-sequencing data generated from *P. oxalicum* strain HP7-1.

**Additional file 2: Table S2.** DEGs identified in *P. oxalicum* strain HP7-1 grown in solid medium containing WR (HP7-1_WR-S) and liquid medium containing WR (HP7-1_WR-L).

**Additional file 3: Figure S1.** Confirmatory analysis of 25 deletion mutants constructed from the parental strain Δ*PoxKu70*. (a) Schematic illustration of deletion mutant construction. (b) PCR analysis. M, 1 kb DNA markers; Lanes 1–3, three transformants for each candidate gene; Lane 4, Δ*PoxKu70*; Lane 5, ddH_2_O. The top panel shows amplification of the region using primers Gene-Left-F/G418VR; the middle panel shows amplification of the region using primers G418VF/Gene-Right-R; the bottom panel shows amplification of the region of the target gene using primers GeneVF/GeneVR. (c) Southern hybridisation analysis of deletion mutant Δ*POX08292*. M, 1 kb DNA markers; Lane 1, Δ*PoxKu70*; Lane 2, Δ*POX08292*-2; Lane 3, Δ*POX08292*-6; Lane 4, Δ*POX08292*-12.

**Additional file 4: Table S3.** Primers used in this study.

**Additional file 5: Table S4.** Candidate regulatory genes knocked out in *P. oxalicum.*

**Additional file 6: Figure S2.** Confirmation of complementary strain C*PoxMBF1*. (a) Schematic illustration of complementary strain construction. (b–g) PCR analysis. M, 1 kb DNA markers; Lane 1, Δ*PoxMBF1*; Lane 2, Δ*PoxKu70*; Lanes 3–5, three transformants of C*PoxMBF1*; (b) PCR using primers bleVF/bleVR; (c) PCR using primers POX05007-V-F/POX05007-V-R; (d) PCR using primers Pro-V-F/Pro-V-R; (e) PCR using primers CPOX08292-F/CPOX08292-R; (f) PCR using primers 5007bleVF/CPOX05007-R–R; (g) PCR using primers CPOX05007-L-F/5007bleVR.

**Additional file 7: Figure S3.** Growth curve of *P*. *oxalicum* mutants Δ*PoxMBF1* and Δ*PoxKu70* in glucose (a) and Avicel (b) media.

## Abbreviations

6-FAM: 6-carboxyfluorescein
BSA: bovine serum albumin
CMCase: carboxymethylcellulase
EMSA: electrophoretic mobility shift assay
DEGs: differentially expressed genes
FPase: filter paper cellulase
HP7-1_WR-S: *P*. *oxalicum* strain HP7-1 grown in solid medium containing wheat bran plus rice straw
HP7-1_WR-L: *P*. *oxalicum* strain HP7-1 grown in liquid medium containing wheat bran plus rice straw
PDA: potato dextrose agar
pNPCase: *p*-nitrophenyl-β-cellobiosidase
pNPGase: *p*-nitrophenyl-β-glucopyranosidase
RT-qPCR: real-time quantitative reverse-transcription PCR
SSF: solid-state fermentation
SmF: submerged fermentation
TBP: TATA-box binding protein
TFs: transcriptional factors
WB: wheat bran
WR: wheat bran plus rice straw
