# Supplementary material for: Differential transcriptomic profiling of filamentous fungus during solid-state and submerged fermentation and identification of an essential regulatory gene PoxMBF1 that directly regulated cellulase and xylanase gene expression
Source: Biotechnol Biofuels. 2019 Apr 30;12:103. doi: 10.1186/s13068-019-1445-4 (PMC6489320; doi:10.1186/s13068-019-1445-4)
Supplement: Supplementary file 1 — Additional file 1: Table S1. Summary of RNA-sequencing data generated from P. oxalicum strain HP7-1. [file 13068_2019_1445_MOESM1_ESM.pdf]

Additional file 1: Table S1. Summary of RNA-sequencing data generated from *Penicillium oxalicum* strain HP7-1.

Samples[a]	Clean Reads	Number of nucleotides (bp)	Overall read alignment rate (%)	Concordant pair alignment rate (%)	Number of expressed genes
HP7-1_WR_S-1	24298252	2429825200	87.62	72.80	8950
HP7-1_WR_S-2	23826040	2382604000	94.89	76.38	8721
HP7-1_WR_S-3	23783778	2378377800	94.88	76.67	8711
HP7-1_WR_L-1	23992762	2399276200	85.95	71.25	8850
HP7-1_WR_L-2	23811528	2381152800	89.70	73.15	8932
HP7-1_WR_L-3	23751856	2375185600	90.11	71.76	8830

[a] WR: wheat bran plus rice straw; S: solid-state fermentation; L: submerged fermentation.
