# Supplementary material for: Differential transcriptomic profiling of filamentous fungus during solid-state and submerged fermentation and identification of an essential regulatory gene PoxMBF1 that directly regulated cellulase and xylanase gene expression
Source: Biotechnol Biofuels. 2019 Apr 30;12:103. doi: 10.1186/s13068-019-1445-4 (PMC6489320; doi:10.1186/s13068-019-1445-4)
Supplement: Supplementary file 2 — Additional file 2: Table S2. DEGs identified in P. oxalicum strain HP7-1 grown in solid medium containing WR (HP7-1_WR-S) and liquid medium containing WR (HP7-1_WR-L). [file 13068_2019_1445_MOESM2_ESM.pdf]

Additional file 2: Table S2. List of 1724 differentially expressed genes in the *Penicillium oxalicum* strain HP7-1 grown in solid medium containing WR (HP7-1_WR-S) as compared with that in liquid medium containing WR (HP7-1_WR-L).

Gene ID		CAZy family	HP7-1_WR_S	HP7-1_WR_L	log2 (HP7-1_WR_L/HP7-1_WR_S)	Regulation (HP7-1_WR_L/HP7-1_WR_S)	Probability
POX00007	Putative beta-xylosidase	GH3;AA5	104.11	5.51	-4.24	Down	0.91
POX00008	Putative exo-alpha-L-1,5-arabinanase	GH33;GH93	784.95	60.96	-3.69	Down	0.95
POX00044	Hypothetical protein	NA	337.39	1562.55	2.21	Up	0.93
POX00046	Hypothetical protein	NA	177.14	67.86	-1.38	Down	0.86
POX00048	Hypothetical protein	NA	18.28	109.29	2.58	Up	0.89
POX00050	Hypothetical protein	CE10	132.83	425.67	1.68	Up	0.90
POX00053	Putative alpha-mannosidase	GH92	236.98	11.26	-4.40	Down	0.93
POX00063	Endo-β-1,4-xylanase	CBM1;GH10	3144.62	288.79	-3.44	Down	0.95
POX00065	Hypothetical protein	NA	550.02	71.46	-2.94	Down	0.94
POX00069	Hypothetical protein	NA	697.30	3166.84	2.18	Up	0.94
POX00071	Hypothetical protein	NA	115.94	47.00	-1.30	Down	0.83
POX00072	Hypothetical protein	NA	150.40	15.64	-3.27	Down	0.92
POX00074	Hypothetical protein	NA	67.28	229.34	1.77	Up	0.89
POX00078	Hypothetical protein	NA	142.05	452.25	1.67	Up	0.90
POX00084	Hypothetical protein	NA	116.01	235.59	1.02	Up	0.81
POX00086	Hypothetical protein	NA	102.83	17.01	-2.60	Down	0.89
POX00089	Putative chitinase	GH18	1279.48	449.53	-1.51	Down	0.90
POX00098	Hypothetical protein	NA	1468.10	618.46	-1.25	Down	0.87
POX00103	Hypothetical protein	NA	478.65	971.87	1.02	Up	0.83
POX00105	Putative exo-beta-1,3-glucanase	GH55	671.10	67.08	-3.32	Down	0.94
POX00108	Hypothetical protein	NA	507.70	2475.62	2.29	Up	0.94
POX00113	Hypothetical protein	NA	59.39	17.64	-1.75	Down	0.81
POX00119	Hypothetical protein	NA	105.15	30.21	-1.80	Down	0.86
POX00121	Hypothetical protein	NA	171.22	366.15	1.10	Up	0.84
POX00122	Hypothetical protein	NA	517.99	1847.04	1.83	Up	0.92
POX00123	Hypothetical protein	NA	40.75	182.84	2.17	Up	0.90
POX00138	Hypothetical protein	NA	274.74	1081.10	1.98	Up	0.93
POX00141	Hypothetical protein	NA	44.87	160.00	1.83	Up	0.89
POX00144	Hypothetical protein	AA7	13.47	60.43	2.17	Up	0.84
POX00147	Hypothetical protein	NA	163.96	498.22	1.60	Up	0.90
POX00148	Hypothetical protein	NA	40.24	93.61	1.22	Up	0.80
POX00152	Hypothetical protein	NA	29.30	111.31	1.93	Up	0.87

POX00157	Hypothetical protein	NA	29.56	84.73	1.52	Up	0.83
POX00160	Hypothetical protein	NA	137.77	435.46	1.66	Up	0.90
POX00167	Hypothetical protein	NA	197.08	975.97	2.31	Up	0.93
POX00169	Hypothetical protein	NA	541.14	163.16	-1.73	Down	0.91
POX00176	Hypothetical protein	NA	492.44	2383.07	2.27	Up	0.94
POX00180	Hypothetical protein	NA	38.30	119.64	1.64	Up	0.86
POX00184	Hypothetical protein	NA	44.69	145.64	1.70	Up	0.87
POX00185	Hypothetical protein	NA	102.78	21.94	-2.23	Down	0.88
POX00186	Hypothetical protein	NA	76.44	177.74	1.22	Up	0.84
POX00197	Putative feruloyl esterase	CE1	110.74	27.79	-1.99	Down	0.88
POX00213	Hypothetical protein	NA	80.13	21.99	-1.87	Down	0.85
POX00214	Hypothetical protein	NA	73.56	24.44	-1.59	Down	0.82
POX00222	Hypothetical protein	NA	178.46	415.81	1.22	Up	0.86
POX00230	Hypothetical protein	NA	151.33	357.83	1.24	Up	0.86
POX00234	Hypothetical protein	NA	196.10	97.94	-1.00	Down	0.80
POX00246	Hypothetical protein	NA	232.13	68.63	-1.76	Down	0.89
POX00250	Hypothetical protein	NA	28.54	93.08	1.71	Up	0.85
POX00260	Hypothetical protein	NA	333.66	61.35	-2.44	Down	0.93
POX00261	Hypothetical protein	NA	54.15	8.74	-2.63	Down	0.84
POX00265	Hypothetical protein	NA	33.90	110.31	1.70	Up	0.86
POX00269	Hypothetical protein	NA	192.10	85.03	-1.18	Down	0.83
POX00286	Hypothetical protein	NA	57.89	200.31	1.79	Up	0.89
POX00287	Hypothetical protein	NA	27.84	128.57	2.21	Up	0.89
POX00291	Hypothetical protein	NA	107.97	322.14	1.58	Up	0.89
POX00293	Hypothetical protein	NA	243.48	1248.15	2.36	Up	0.94
POX00306	Hypothetical protein	NA	62.45	142.77	1.19	Up	0.82
POX00310	Hypothetical protein	NA	48.16	143.67	1.58	Up	0.87
POX00331	Hypothetical protein	NA	206.60	37.38	-2.47	Down	0.92
POX00334	Hypothetical protein	NA	68.61	13.50	-2.35	Down	0.86
POX00342	Hypothetical protein	NA	63.98	16.47	-1.96	Down	0.84
POX00347	Hypothetical protein	CBM21	514.42	75.15	-2.78	Down	0.94
POX00350	Hypothetical protein	NA	31.50	95.90	1.61	Up	0.85
POX00351	Hypothetical protein	NA	165.45	31.97	-2.37	Down	0.91
POX00365	Hypothetical protein	NA	460.69	204.14	-1.17	Down	0.85
POX00368	Hypothetical protein	NA	117.31	321.52	1.45	Up	0.88
POX00373	Hypothetical protein	NA	23.41	76.33	1.71	Up	0.83
POX00377	Hypothetical protein	NA	68.97	13.79	-2.32	Down	0.86
POX00379	Hypothetical protein	NA	97.39	40.95	-1.25	Down	0.81

POX00401	Hypothetical protein	NA	19.40	95.95	2.31	Up	0.88
POX00406	Hypothetical protein	NA	186.39	513.08	1.46	Up	0.89
POX00414	Hypothetical protein	NA	98.20	347.03	1.82	Up	0.91
POX00429	Hypothetical protein	NA	610.48	63.86	-3.26	Down	0.94
POX00430	Hypothetical protein	NA	93.12	382.05	2.04	Up	0.92
POX00437	Hypothetical protein	NA	30.39	128.69	2.08	Up	0.89
POX00441	Hypothetical protein	NA	28.92	103.72	1.84	Up	0.87
POX00445	Hypothetical protein	NA	227.00	482.06	1.09	Up	0.84
POX00449	Hypothetical protein	NA	808.00	258.25	-1.65	Down	0.91
POX00457	Hypothetical protein	NA	21.92	74.54	1.77	Up	0.84
POX00481	Hypothetical protein	NA	88.73	306.02	1.79	Up	0.90
POX00486	Hypothetical protein	NA	27.13	90.68	1.74	Up	0.85
POX00499	Hypothetical protein	NA	3820.76	1516.65	-1.33	Down	0.89
POX00504	Hypothetical protein	NA	21.42	87.87	2.04	Up	0.86
POX00523	Hypothetical protein	NA	180.29	32.66	-2.46	Down	0.91
POX00540	Hypothetical protein	NA	945.27	129.12	-2.87	Down	0.94
POX00555	Hypothetical protein	NA	2.07	53.60	4.69	Up	0.86
POX00585	Hypothetical protein	NA	22.21	88.33	1.99	Up	0.86
POX00588	Hypothetical protein	NA	126.83	321.03	1.34	Up	0.87
POX00599	Hypothetical protein	NA	1127.18	529.20	-1.09	Down	0.85
POX00617	Putative beta-galactosidase	GH2	87.86	20.35	-2.11	Down	0.87
POX00618	Hypothetical protein	NA	1224.05	162.93	-2.91	Down	0.94
POX00621	Hypothetical protein	NA	90.47	31.70	-1.51	Down	0.83
POX00641	Hypothetical protein	NA	44.52	6.33	-2.81	Down	0.83
POX00645	Hypothetical protein	NA	69.25	177.00	1.35	Up	0.85
POX00658	Hypothetical protein	NA	33.34	92.37	1.47	Up	0.83
POX00659	Hypothetical protein	NA	175.66	1961.55	3.48	Up	0.95
POX00665	Hypothetical protein	NA	224.10	2305.14	3.36	Up	0.95
POX00667	Hypothetical protein	NA	24.65	571.58	4.54	Up	0.95
POX00668	Feruloyl esterase	NA	112.65	5.38	-4.39	Down	0.91
POX00669	Hypothetical protein	NA	9.74	45.10	2.21	Up	0.81
POX00670	Hypothetical protein	NA	27.63	179.72	2.70	Up	0.92
POX00673	Hypothetical protein	NA	147.67	434.67	1.56	Up	0.90
POX00693	Hypothetical protein	NA	26.26	225.65	3.10	Up	0.93
POX00694	Putative chitosanase	GH75	80.13	3.94	-4.35	Down	0.89
POX00695	Hypothetical protein	NA	29.14	91.61	1.65	Up	0.85
POX00700	Hypothetical protein	NA	157.67	55.49	-1.51	Down	0.87
POX00718	Hypothetical protein	NA	7.92	46.78	2.56	Up	0.83

POX00719	Hypothetical protein	NA	130.56	33.79	-1.95	Down	0.88
POX00724	Hypothetical protein	NA	72.78	0.91	-6.32	Down	0.90
POX00725	Hypothetical protein	NA	87.79	2.62	-5.06	Down	0.90
POX00726	Hypothetical protein	NA	73.41	0.01	-12.84	Down	0.94
POX00727	Feruloyl esterase	NA	113.70	35.53	-1.68	Down	0.86
POX00732	Hypothetical protein	NA	204.11	19.45	-3.39	Down	0.93
POX00733	Hypothetical protein	NA	31.55	78.91	1.32	Up	0.80
POX00740	Hypothetical protein	NA	131.65	9.17	-3.84	Down	0.91
POX00759	Putative beta-mannosidase	GH2	75.57	20.58	-1.88	Down	0.85
POX00765	Hypothetical protein	NA	163.92	371.10	1.18	Up	0.85
POX00769	Hypothetical protein	NA	5.50	97.51	4.15	Up	0.90
POX00770	Hypothetical protein	NA	82.00	268.44	1.71	Up	0.90
POX00776	Hypothetical protein	NA	19.06	156.23	3.04	Up	0.92
POX00777	Hypothetical protein	NA	22.44	98.69	2.14	Up	0.88
POX00805	Hypothetical protein	NA	3.67	104.89	4.84	Up	0.91
POX00815	Hypothetical protein	NA	290.49	786.14	1.44	Up	0.89
POX00820	Hypothetical protein	NA	58.11	5.48	-3.41	Down	0.86
POX00826	Hypothetical protein	NA	65.13	178.19	1.45	Up	0.87
POX00832	Hypothetical protein	NA	29.46	76.75	1.38	Up	0.81
POX00836	Hypothetical protein	NA	454.84	24.62	-4.21	Down	0.94
POX00842	Hypothetical protein	NA	13.93	115.19	3.05	Up	0.90
POX00854	Hypothetical protein	NA	57.35	130.92	1.19	Up	0.82
POX00859	Hypothetical protein	NA	7.76	153.92	4.31	Up	0.92
POX00860	Hypothetical protein	NA	9.52	57.91	2.60	Up	0.85
POX00861	Hypothetical protein	NA	66.54	223.29	1.75	Up	0.89
POX00862	Hypothetical protein	NA	48.91	353.38	2.85	Up	0.93
POX00863	Hypothetical protein	NA	10.23	46.44	2.18	Up	0.81
POX00865	Hypothetical protein	NA	50.38	217.61	2.11	Up	0.91
POX00867	Hypothetical protein	NA	151.02	325.03	1.11	Up	0.84
POX00870	Hypothetical protein	NA	68.59	156.84	1.19	Up	0.83
POX00873	Hypothetical protein	NA	24.76	386.98	3.97	Up	0.94
POX00878	Hypothetical protein	NA	175.63	72.26	-1.28	Down	0.84
POX00884	Hypothetical protein	NA	25.90	183.28	2.82	Up	0.92
POX00887	Hypothetical protein	NA	23.98	121.03	2.34	Up	0.89
POX00913	Hypothetical protein	NA	324.40	863.20	1.41	Up	0.89
POX00914	Hypothetical protein	NA	210.23	102.69	-1.03	Down	0.81
POX00917	Hypothetical protein	NA	53.20	149.70	1.49	Up	0.86
POX00925	Hypothetical protein	NA	62.37	9.41	-2.73	Down	0.86

POX00932	Putative Hsp70 molecular chaperone	NA	134.62	762.67	2.50	Up	0.94
POX00933	Hypothetical protein	NA	169.60	49.32	-1.78	Down	0.89
POX00941	Putative chitin glucanosyltransferase	GH16	17.80	57.80	1.70	Up	0.81
POX00943	Hypothetical protein	NA	55.98	0.21	-8.04	Down	0.90
POX00944	Hypothetical protein	NA	255.39	39.33	-2.70	Down	0.92
POX00951	Hypothetical protein	NA	74.26	334.73	2.17	Up	0.92
POX00968	Putative beta-glucosidase	GH3	240.49	68.93	-1.80	Down	0.90
POX00971	Hypothetical protein	NA	55.06	149.98	1.45	Up	0.86
POX00972	Hypothetical protein	NA	36.08	152.49	2.08	Up	0.90
POX00976	Hypothetical protein	NA	542.83	1516.73	1.48	Up	0.90
POX00977	Hypothetical protein	NA	24.84	136.53	2.46	Up	0.90
POX00979	Hypothetical protein	NA	110.36	384.33	1.80	Up	0.91
POX00998	Hypothetical protein	NA	51.99	152.41	1.55	Up	0.87
POX01003	Hypothetical protein	NA	26.18	107.61	2.04	Up	0.88
POX01010	Hypothetical protein	NA	57.92	11.93	-2.28	Down	0.84
POX01017	Hypothetical protein	NA	51.80	124.27	1.26	Up	0.82
POX01021	Hypothetical protein	NA	16.59	89.50	2.43	Up	0.88
POX01022	Hypothetical protein	NA	40.69	163.47	2.01	Up	0.90
POX01030	Hypothetical protein	NA	32.78	110.18	1.75	Up	0.86
POX01031	Hypothetical protein	NA	117.49	476.79	2.02	Up	0.92
POX01032	Hypothetical protein	NA	235.08	593.98	1.34	Up	0.88
POX01033	Hypothetical protein	NA	2562.40	1036.96	-1.31	Down	0.88
POX01041	Hypothetical protein	NA	1194.33	247.59	-2.27	Down	0.93
POX01050	Hypothetical protein	NA	56.94	143.71	1.34	Up	0.84
POX01055	Hypothetical protein	NA	424.19	2.51	-7.40	Down	0.97
POX01067	Hypothetical protein	NA	131.92	603.55	2.19	Up	0.93
POX01073	Hypothetical protein	NA	96.23	388.64	2.01	Up	0.92
POX01075	Hypothetical protein	NA	144.02	651.61	2.18	Up	0.93
POX01083	Hypothetical protein	NA	35.15	96.31	1.45	Up	0.83
POX01087	Hypothetical protein	NA	242.93	69.49	-1.81	Down	0.90
POX01088	Hypothetical protein	NA	167.55	620.71	1.89	Up	0.92
POX01096	Hypothetical protein	NA	75.68	169.23	1.16	Up	0.82
POX01102	Hypothetical protein	NA	20.74	73.69	1.83	Up	0.84
POX01104	Hypothetical protein	NA	52.48	180.38	1.78	Up	0.89
POX01113	Hypothetical protein	NA	316.48	1207.73	1.93	Up	0.92
POX01120	Hypothetical protein	NA	29.22	93.75	1.68	Up	0.85
POX01134	Hypothetical protein	NA	506.48	1198.45	1.24	Up	0.87

POX01148	Hypothetical protein	NA	30.04	151.25	2.33	Up	0.90
POX01155	Hypothetical protein	NA	535.72	46.74	-3.52	Down	0.94
POX01158	SUN domain-containing protein	GH132	272.51	800.37	1.55	Up	0.90
POX01161	Hypothetical protein	NA	25.99	73.84	1.51	Up	0.82
POX01165	Hypothetical protein	NA	0.01	47.28	12.21	Up	0.90
POX01166	Putative endo-beta-1,4-glucanase	CBM1;GH5	2027.51	784.35	-1.37	Down	0.89
POX01167	Hypothetical protein	NA	136.46	50.41	-1.44	Down	0.85
POX01168	Hypothetical protein	NA	701.78	139.41	-2.33	Down	0.93
POX01169	Hypothetical protein	NA	3612.82	39.87	-6.50	Down	0.97
POX01170	Hypothetical protein	NA	552.94	14.05	-5.30	Down	0.95
POX01171	Hypothetical protein	NA	1351.87	16.45	-6.36	Down	0.96
POX01172	Hypothetical protein	NA	869.25	9.39	-6.53	Down	0.96
POX01173	Hypothetical protein	NA	2550.01	21.15	-6.91	Down	0.97
POX01174	Hypothetical protein	NA	3048.70	53.13	-5.84	Down	0.96
POX01175	Hypothetical protein	NA	1059.62	120.75	-3.13	Down	0.95
POX01176	Hypothetical protein	NA	6181.80	70.03	-6.46	Down	0.97
POX01177	Hypothetical protein	NA	1535.29	10.18	-7.24	Down	0.98
POX01178	Hypothetical protein	NA	1107.71	13.00	-6.41	Down	0.96
POX01179	Hypothetical protein	NA	4403.58	43.76	-6.65	Down	0.97
POX01180	Hypothetical protein	NA	3454.13	34.59	-6.64	Down	0.97
POX01181	Hypothetical protein	NA	2557.31	27.91	-6.52	Down	0.97
POX01182	Hypothetical protein	NA	4023.25	37.72	-6.74	Down	0.97
POX01183	Hypothetical protein	NA	99.42	12.84	-2.95	Down	0.89
POX01184	Hypothetical protein	NA	444.53	46.53	-3.26	Down	0.94
POX01203	Hypothetical protein	NA	51.94	400.82	2.95	Up	0.94
POX01217	Hypothetical protein	NA	13.42	62.33	2.22	Up	0.84
POX01218	Putative acetyl xylan esterase	CBM1;CE1	4404.43	369.61	-3.57	Down	0.95
POX01219	Putative exo-beta-1,3-galactanase	CBM35;GH43	544.53	60.18	-3.18	Down	0.94
POX01222	Hypothetical protein	NA	115.46	303.31	1.39	Up	0.87
POX01223	Hypothetical protein	NA	67.77	10.90	-2.64	Down	0.86
POX01224	Hypothetical protein	NA	51.14	11.46	-2.16	Down	0.82
POX01225	Putative rhamnogalacturonan alpha-L-rhamnopyranohydrolase	GH28	355.52	39.85	-3.16	Down	0.94
POX01251	Hypothetical protein	NA	82.82	236.71	1.52	Up	0.88
POX01253	Hypothetical protein	NA	22.46	63.49	1.50	Up	0.80
POX01274	Putative exo-alpha-L-1,5-arabinanase	GH93	117.72	22.68	-2.38	Down	0.89
POX01283	Hypothetical protein	NA	138.28	331.81	1.26	Up	0.86

POX01286	Hypothetical protein	NA	248.43	105.51	-1.24	Down	0.85
POX01287	Hypothetical protein	NA	205.80	99.80	-1.04	Down	0.81
POX01291	Hypothetical protein	NA	33.68	149.14	2.15	Up	0.90
POX01301	Hypothetical protein	NA	430.61	1821.43	2.08	Up	0.93
POX01306	Hypothetical protein	NA	34.80	120.05	1.79	Up	0.87
POX01308	Hypothetical protein	NA	170.70	58.54	-1.54	Down	0.87
POX01317	Hypothetical protein	NA	1085.08	5315.22	2.29	Up	0.94
POX01318	Hypothetical protein	NA	83.78	2.60	-5.01	Down	0.90
POX01319	Hypothetical protein	NA	70.47	2.48	-4.83	Down	0.89
POX01320	Hypothetical protein	NA	21.75	95.07	2.13	Up	0.87
POX01329	Putative chitin deacetylase	CE4	147.14	47.78	-1.62	Down	0.87
POX01331	Hypothetical protein	NA	48.13	11.80	-2.03	Down	0.81
POX01340	Hypothetical protein	NA	17.29	84.33	2.29	Up	0.87
POX01342	Hypothetical protein	NA	120.74	37.51	-1.69	Down	0.86
POX01344	Hypothetical protein	NA	320.88	66.04	-2.28	Down	0.92
POX01346	Putative chitin synthase	NA	56.97	2.15	-4.73	Down	0.87
POX01353	Hypothetical protein	NA	94.08	11.52	-3.03	Down	0.89
POX01356	Glucoamylase Amy15A	CBM20;GH15	36.31	1351.81	5.22	Up	0.96
POX01381	Chitin binding domain-containing protein	AA11	17.94	82.91	2.21	Up	0.87
POX01383	Hypothetical protein	NA	189.10	999.11	2.40	Up	0.94
POX01390	Hypothetical protein	CE1	135.46	29.60	-2.19	Down	0.89
POX01391	Hypothetical protein	AA1	73.36	12.66	-2.53	Down	0.87
POX01392	Hypothetical protein	NA	100.42	6.21	-4.01	Down	0.90
POX01394	Hypothetical protein	NA	161.04	474.67	1.56	Up	0.90
POX01407	Hypothetical protein	NA	124.93	276.70	1.15	Up	0.84
POX01408	Hypothetical protein	NA	339.86	127.34	-1.42	Down	0.88
POX01412	Hypothetical protein	NA	38.43	2.81	-3.77	Down	0.82
POX01420	Hypothetical protein	NA	89.43	242.73	1.44	Up	0.87
POX01423	Hypothetical protein	NA	232.28	48.10	-2.27	Down	0.91
POX01431	Hypothetical protein	AA1	83.97	17.79	-2.24	Down	0.87
POX01444	Putative alpha-glucosidase	GH31	31.62	81.97	1.37	Up	0.81
POX01450	Hypothetical protein	NA	40.74	129.45	1.67	Up	0.87
POX01456	Hypothetical protein	NA	26.22	95.44	1.86	Up	0.86
POX01462	Hypothetical protein	NA	78.73	12.25	-2.68	Down	0.88
POX01470	Putative chitinase	GH18	51.17	402.83	2.98	Up	0.94
POX01484	Hypothetical protein	NA	50.57	147.36	1.54	Up	0.86
POX01489	Hypothetical protein	NA	38.59	1.02	-5.25	Down	0.83
POX01491	Hypothetical protein	NA	56.67	177.49	1.65	Up	0.88

POX01494	Hypothetical protein	NA	54.00	196.35	1.86	Up	0.89
POX01521	Hypothetical protein	NA	110.26	20.00	-2.46	Down	0.89
POX01523	Hypothetical protein	NA	441.05	193.14	-1.19	Down	0.86
POX01524	Expansin-like	CBM63	26.15	94.43	1.85	Up	0.86
POX01531	Hypothetical protein	NA	26.73	101.92	1.93	Up	0.87
POX01532	Hypothetical protein	NA	84.04	329.39	1.97	Up	0.91
POX01535	Hypothetical protein	NA	369.18	114.72	-1.69	Down	0.90
POX01539	Hypothetical protein	NA	72.05	167.65	1.22	Up	0.83
POX01554	Hypothetical protein	NA	22.75	1290.84	5.83	Up	0.96
POX01556	Hypothetical protein	NA	27.17	72.69	1.42	Up	0.81
POX01561	Hypothetical protein	NA	25.12	78.30	1.64	Up	0.83
POX01573	Hypothetical protein	NA	75.44	16.53	-2.19	Down	0.86
POX01576	Hypothetical protein	NA	9.25	43.54	2.24	Up	0.81
POX01578	Hypothetical protein	NA	35.68	196.62	2.46	Up	0.91
POX01579	Hypothetical protein	NA	58.42	3.41	-4.10	Down	0.87
POX01583	Hypothetical protein	NA	33.74	110.65	1.71	Up	0.86
POX01591	Hypothetical protein	NA	77.93	23.44	-1.73	Down	0.84
POX01596	Hypothetical protein	NA	18.57	119.90	2.69	Up	0.90
POX01601	Hypothetical protein	NA	69.53	5.95	-3.55	Down	0.88
POX01602	Hypothetical protein	NA	39.19	1.11	-5.14	Down	0.83
POX01606	Hypothetical protein	NA	53.08	13.54	-1.97	Down	0.82
POX01614	Hypothetical protein	NA	15.01	131.22	3.13	Up	0.91
POX01617	Hypothetical protein	NA	25.27	81.96	1.70	Up	0.84
POX01626	Hypothetical protein	NA	46.26	131.21	1.50	Up	0.86
POX01635	Hypothetical protein	NA	19.14	89.99	2.23	Up	0.87
POX01636	Hypothetical protein	NA	325.44	144.83	-1.17	Down	0.85
POX01637	Hypothetical protein	NA	49.63	6.29	-2.98	Down	0.84
POX01639	Hypothetical protein	NA	32.58	110.55	1.76	Up	0.86
POX01660	Hypothetical protein	NA	87.38	29.14	-1.58	Down	0.84
POX01664	Hypothetical protein	NA	248.10	1251.28	2.33	Up	0.94
POX01671	Hypothetical protein	NA	74.82	254.43	1.77	Up	0.90
POX01682	Hypothetical protein	NA	3130.30	110.85	-4.82	Down	0.96
POX01688	Hypothetical protein	NA	333.78	673.78	1.01	Up	0.83
POX01691	Hypothetical protein	NA	60.18	146.09	1.28	Up	0.84
POX01692	Hypothetical protein	NA	245.62	1140.80	2.22	Up	0.93
POX01693	Hypothetical protein	NA	208.50	889.22	2.09	Up	0.93
POX01694	Hypothetical protein	NA	894.58	2516.47	1.49	Up	0.90
POX01695	Hypothetical protein	NA	1315.02	6892.32	2.39	Up	0.94

POX01697	Hypothetical protein	NA	81.63	290.87	1.83	Up		0.90
POX01701	Hypothetical protein	CE9	0.82	54.41	6.05	Up		0.87
POX01708	Hypothetical protein	NA	596.96	1870.00	1.65	Up		0.91
POX01709	Hypothetical protein	NA	42.81	9.43	-2.18	Down		0.80
POX01738	Hypothetical protein	NA	23.27	110.51	2.25	Up		0.89
POX01750	Hypothetical protein	NA	98.14	252.30	1.36	Up		0.87
POX01755	Hypothetical protein	NA	71.16	24.34	-1.55	Down		0.82
POX01758	Hypothetical protein	NA	50.79	160.05	1.66	Up		0.88
POX01760	Hypothetical protein	NA	38.44	142.74	1.89	Up		0.88
POX01761	Hypothetical protein	NA	55.24	171.69	1.64	Up		0.88
POX01762	Hypothetical protein	NA	43.10	5.76	-2.90	Down		0.82
POX01763	Hypothetical protein	NA	52.23	152.66	1.55	Up		0.87
POX01764	Hypothetical protein	NA	6619.39	1582.66	-2.06	Down		0.93
POX01775	Hypothetical protein	NA	28.49	100.88	1.82	Up		0.86
POX01776	Hypothetical protein	NA	42.86	123.89	1.53	Up		0.86
POX01777	Hypothetical protein	NA	2003.68	631.07	-1.67	Down		0.91
POX01787	Hypothetical protein	NA	101.44	27.95	-1.86	Down		0.87
POX01798	Hypothetical protein	NA	213.14	903.03	2.08	Up		0.93
POX01799	Hypothetical protein	NA	2083.03	369.82	-2.49	Down		0.94
POX01802	Hypothetical protein	NA	316.81	157.61	-1.01	Down		0.82
POX01803	Hypothetical protein	NA	46.13	11.34	-2.02	Down		0.80
POX01807	Hypothetical protein	NA	36.78	129.47	1.82	Up		0.88
POX01808	Hypothetical protein	NA	170.19	645.49	1.92	Up		0.92
POX01825	Hypothetical protein	GT90	168.19	59.76	-1.49	Down		0.87
POX01826	Hypothetical protein	NA	3313.81	185.23	-4.16	Down		0.95
POX01832	Hypothetical protein	NA	795.17	203.00	-1.97	Down		0.92
POX01833	Putative alpha-mannosyltransferase	GT71	105.73	22.64	-2.22	Down		0.88
POX01834	Hypothetical protein	NA	54.74	256.28	2.23	Up		0.92
POX01838	Hypothetical protein	NA	840.77	172.75	-2.28	Down		0.93
POX01840	Hypothetical protein	NA	341.57	46.78	-2.87	Down		0.93
POX01841	Hypothetical protein	NA	73.49	26.97	-1.45	Down		0.81
POX01852	Hypothetical protein	AA4;AA7	39.76	115.03	1.53	Up		0.85
POX01855	Hypothetical protein	NA	25.48	67.96	1.42	Up		0.80
POX01867	Hypothetical protein	NA	194.47	90.69	-1.10	Down		0.82
POX01876	Hypothetical protein	NA	48.75	110.60	1.18	Up		0.81
POX01880	Hypothetical protein	NA	182.88	373.47	1.03	Up		0.82
POX01886	Hypothetical protein	NA	17.69	58.93	1.74	Up		0.81
POX01890	Hypothetical protein	NA	86.33	15.82	-2.45	Down		0.88

POX01896	Endo-beta-1,4-glucanase Cel5C	CBM1;CBM46;GH5	723.03	183.81	-1.98	Down	0.92
POX01897	Hypothetical protein	NA	111.43	6.32	-4.14	Down	0.91
POX01898	Hypothetical protein	NA	35.69	96.70	1.44	Up	0.83
POX01912	Hypothetical protein	NA	79.46	5.61	-3.82	Down	0.89
POX01918	Hypothetical protein	NA	120.92	29.51	-2.03	Down	0.88
POX01921	Putative alpha-L-arabinofuranosidase	GH43	148.72	16.01	-3.22	Down	0.92
POX01924	Hypothetical protein	NA	331.99	155.10	-1.10	Down	0.83
POX01926	Hypothetical protein	NA	156.90	570.74	1.86	Up	0.92
POX01927	Hypothetical protein	NA	34.38	620.79	4.17	Up	0.95
POX01929	Hypothetical protein	NA	778.31	128.12	-2.60	Down	0.94
POX01931	Hypothetical protein	NA	89.31	196.91	1.14	Up	0.83
POX01933	Hypothetical protein	NA	77.98	23.67	-1.72	Down	0.84
POX01945	Hypothetical protein	AA7	114.32	27.72	-2.04	Down	0.88
POX01947	Hypothetical protein	NA	78.25	194.99	1.32	Up	0.85
POX01964	Hypothetical protein	NA	585.01	196.48	-1.57	Down	0.90
POX01971	Hypothetical protein	NA	289.20	53.35	-2.44	Down	0.92
POX01973	Hypothetical protein	NA	1930.77	546.92	-1.82	Down	0.92
POX01976	Hypothetical protein	NA	271.43	1399.46	2.37	Up	0.94
POX01981	Hypothetical protein	NA	201.96	66.78	-1.60	Down	0.88
POX01983	Hypothetical protein	NA	19.01	67.77	1.83	Up	0.83
POX01986	Hypothetical protein	AA7	409.93	23.53	-4.12	Down	0.94
POX01987	Hypothetical protein	NA	68.53	151.98	1.15	Up	0.82
POX01995	Hypothetical protein	NA	49.02	7.68	-2.67	Down	0.83
POX01996	Hypothetical protein	NA	69.82	24.93	-1.49	Down	0.81
POX02011	Hypothetical protein	NA	262.33	43.80	-2.58	Down	0.92
POX02014	Hypothetical protein	NA	94.09	17.39	-2.44	Down	0.88
POX02016	Hypothetical protein	NA	103.74	37.33	-1.47	Down	0.84
POX02023	Hypothetical protein	NA	178.31	475.16	1.41	Up	0.88
POX02026	Hypothetical protein	NA	102.04	515.43	2.34	Up	0.93
POX02039	Hypothetical protein	NA	30.52	92.38	1.60	Up	0.84
POX02044	Hypothetical protein	NA	34.45	270.02	2.97	Up	0.93
POX02048	Hypothetical protein	NA	108.68	316.45	1.54	Up	0.89
POX02051	Hypothetical protein	NA	83.91	9.50	-3.14	Down	0.89
POX02052	Hypothetical protein	NA	58.26	6.16	-3.24	Down	0.86
POX02067	Hypothetical protein	NA	853.05	383.47	-1.15	Down	0.86
POX02068	Hypothetical protein	NA	510.28	162.59	-1.65	Down	0.90
POX02069	Hypothetical protein	NA	120.39	37.15	-1.70	Down	0.86

POX02072	Hypothetical protein	NA	6535.06	2491.25	-1.39	Down		0.89
POX02079	Hypothetical protein	NA	124.01	49.51	-1.32	Down		0.83
POX02082	Hypothetical protein	NA	454.94	1572.15	1.79	Up		0.92
POX02083	Hypothetical protein	NA	92.73	0.29	-8.30	Down		0.94
POX02091	Hypothetical protein	NA	46.65	104.76	1.17	Up		0.80
POX02104	Hypothetical protein	NA	198.25	470.92	1.25	Up		0.86
POX02113	Hypothetical protein	NA	34.18	119.83	1.81	Up		0.87
POX02121	Hypothetical protein	NA	0.01	25.79	11.33	Up		0.82
POX02122	Hypothetical protein	NA	29.07	118.68	2.03	Up		0.88
POX02125	Hypothetical protein	NA	326.08	161.24	-1.02	Down		0.82
POX02127	Hypothetical protein	NA	90.06	17.08	-2.40	Down		0.88
POX02133	Hypothetical protein	NA	119.34	264.65	1.15	Up		0.84
POX02137	Hypothetical protein	NA	193.33	1370.57	2.83	Up		0.94
POX02153	Hypothetical protein	NA	227.08	107.02	-1.09	Down		0.82
POX02157	Hypothetical protein	NA	125.42	47.14	-1.41	Down		0.84
POX02160	Hypothetical protein	NA	67.39	187.57	1.48	Up		0.87
POX02163	Hypothetical protein	NA	415.23	16.26	-4.67	Down		0.94
POX02164	Hypothetical protein	NA	37.26	102.69	1.46	Up		0.84
POX02170	Hypothetical protein	NA	247.47	1122.62	2.18	Up		0.93
POX02182	Hypothetical protein	NA	108.89	283.36	1.38	Up		0.87
POX02188	Hypothetical protein	NA	214.90	23.04	-3.22	Down		0.93
POX02195	Hypothetical protein	NA	85.81	224.17	1.39	Up		0.87
POX02211	Hypothetical protein	NA	251.96	118.07	-1.09	Down		0.83
POX02216	Hypothetical protein	NA	85.00	228.08	1.42	Up		0.87
POX02220	Hypothetical protein	NA	125.21	54.90	-1.19	Down		0.82
POX02221	Hypothetical protein	NA	83.55	19.84	-2.07	Down		0.86
POX02223	Hypothetical protein	NA	86.33	14.06	-2.62	Down		0.88
POX02227	Hypothetical protein	NA	21.64	301.96	3.80	Up		0.94
POX02229	Hypothetical protein	NA	106.87	46.30	-1.21	Down		0.81
POX02231	Hypothetical protein	NA	1421.95	467.44	-1.61	Down		0.91
POX02233	Hypothetical protein	NA	91.27	20.65	-2.14	Down		0.87
POX02234	Hypothetical protein	NA	73.27	14.17	-2.37	Down		0.86
POX02238	Hypothetical protein	NA	163.90	58.35	-1.49	Down		0.87
POX02240	Hypothetical protein	NA	243.23	107.55	-1.18	Down		0.84
POX02244	Hypothetical protein	NA	59.21	250.86	2.08	Up		0.91
POX02248	Hypothetical protein	NA	75.25	233.49	1.63	Up		0.89
POX02264	Hypothetical protein	NA	337.79	1612.57	2.26	Up		0.94
POX02269	Hypothetical protein	NA	67.31	16.15	-2.06	Down		0.84

POX02270	Hypothetical protein	NA	39.55	5.77	-2.78	Down	0.81
POX02272	Hypothetical protein	NA	66.37	142.68	1.10	Up	0.81
POX02282	Hypothetical protein	NA	253.17	69.31	-1.87	Down	0.90
POX02284	Peptidoglycan binding domain-containing protein	CBM50	930.59	2.08	-8.81	Down	0.99
POX02286	Hypothetical protein	NA	5214.41	1941.67	-1.43	Down	0.90
POX02287	Hypothetical protein	NA	103.33	18.86	-2.45	Down	0.89
POX02288	Hypothetical protein	NA	154.67	0.82	-7.57	Down	0.95
POX02291	Hypothetical protein	NA	116.34	23.14	-2.33	Down	0.89
POX02292	Hypothetical protein	NA	457.36	1513.40	1.73	Up	0.92
POX02294	Hypothetical protein	NA	123.63	270.44	1.13	Up	0.83
POX02297	Hypothetical protein	NA	29.47	85.59	1.54	Up	0.83
POX02298	Hypothetical protein	NA	41.91	8.17	-2.36	Down	0.81
POX02306	Hypothetical protein	NA	240.98	1314.22	2.45	Up	0.94
POX02308	Cellulose monooxygenase Cel61A	AA9	3002.10	426.45	-2.82	Down	0.95
POX02329	Hypothetical protein	NA	65.66	216.81	1.72	Up	0.89
POX02332	Hypothetical protein	NA	9.12	56.56	2.63	Up	0.85
POX02334	Hypothetical protein	NA	30.26	94.82	1.65	Up	0.85
POX02337	Hypothetical protein	NA	62.56	11.41	-2.45	Down	0.85
POX02342	Hypothetical protein	NA	17.75	56.10	1.66	Up	0.80
POX02346	Hypothetical protein	NA	131.76	21.03	-2.65	Down	0.90
POX02354	Hypothetical protein	NA	59.07	10.31	-2.52	Down	0.85
POX02361	Hypothetical protein	NA	216.97	101.04	-1.10	Down	0.82
POX02365	Hypothetical protein	NA	57.09	16.81	-1.76	Down	0.81
POX02380	Hypothetical protein	NA	37.59	101.28	1.43	Up	0.83
POX02386	Hypothetical protein	NA	25.26	91.83	1.86	Up	0.86
POX02390	Hypothetical protein	NA	112.31	380.30	1.76	Up	0.90
POX02391	Hypothetical protein	NA	36.57	93.24	1.35	Up	0.82
POX02392	Hypothetical protein	NA	9.51	295.40	4.96	Up	0.94
POX02393	Hypothetical protein	NA	105.17	472.37	2.17	Up	0.92
POX02402	Hypothetical protein	NA	255.28	1074.54	2.07	Up	0.93
POX02405	Hypothetical protein	NA	1190.19	317.88	-1.90	Down	0.92
POX02407	Hypothetical protein	NA	1045.72	268.62	-1.96	Down	0.92
POX02411	Hypothetical protein	NA	308.66	100.16	-1.62	Down	0.89
POX02412	Putative glucoamylase	CBM20;GH15	38.13	157.92	2.05	Up	0.90
POX02414	Hypothetical protein	NA	52.85	7.90	-2.74	Down	0.84
POX02420	Hypothetical protein	NA	70.41	230.02	1.71	Up	0.89
POX02423	Putative beta-1,3-glucanase	GH64	93.85	22.49	-2.06	Down	0.87

POX02430	Hypothetical protein	NA	37.99	104.95	1.47	Up	0.84
POX02436	Hypothetical protein	NA	278.18	13.90	-4.32	Down	0.94
POX02437	Hypothetical protein	NA	73.38	17.87	-2.04	Down	0.85
POX02438	Hypothetical protein	NA	103.25	31.85	-1.70	Down	0.86
POX02439	Hypothetical protein	NA	44.04	129.52	1.56	Up	0.86
POX02444	Hypothetical protein	AA2	50.35	461.11	3.20	Up	0.94
POX02457	Hypothetical protein	NA	35.27	158.70	2.17	Up	0.90
POX02461	Hypothetical protein	NA	274.00	69.49	-1.98	Down	0.91
POX02466	Hypothetical protein	NA	20.25	86.48	2.09	Up	0.87
POX02468	Hypothetical protein	NA	249.70	96.24	-1.38	Down	0.87
POX02469	Hypothetical protein	NA	47.41	10.12	-2.23	Down	0.82
POX02470	Hypothetical protein	NA	130.45	289.72	1.15	Up	0.84
POX02473	Hypothetical protein	NA	169.57	9.93	-4.09	Down	0.92
POX02475	Hypothetical protein	NA	4.94	71.68	3.86	Up	0.88
POX02480	Hypothetical protein	NA	42.37	116.89	1.46	Up	0.85
POX02490	Putative cellobiohydrolase	GH7	50.60	4.45	-3.51	Down	0.85
POX02493	Hypothetical protein	NA	60.09	18.51	-1.70	Down	0.81
POX02496	Hypothetical protein	NA	177.51	67.54	-1.39	Down	0.86
POX02497	Hypothetical protein	NA	191.53	87.87	-1.12	Down	0.82
POX02503	Hypothetical protein	NA	53.60	159.91	1.58	Up	0.87
POX02504	Hypothetical protein	NA	24.36	69.19	1.51	Up	0.81
POX02520	Hypothetical protein	NA	369.93	81.60	-2.18	Down	0.92
POX02529	Hypothetical protein	NA	77.08	301.56	1.97	Up	0.91
POX02531	Hypothetical protein	NA	98.63	304.18	1.62	Up	0.89
POX02544	Hypothetical protein	NA	15.12	55.43	1.87	Up	0.82
POX02553	Hypothetical protein	NA	99.15	631.16	2.67	Up	0.94
POX02559	Hypothetical protein	NA	17.02	58.30	1.78	Up	0.81
POX02569	Hypothetical protein	NA	186.20	52.98	-1.81	Down	0.89
POX02591	Putative carbohydrate acetylesterase	CE16	387.64	114.29	-1.76	Down	0.91
POX02592	Hypothetical protein	NA	32.84	100.62	1.62	Up	0.85
POX02594	Hypothetical protein	NA	135.12	750.85	2.47	Up	0.94
POX02601	Hypothetical protein	NA	137.01	57.75	-1.25	Down	0.83
POX02618	Hypothetical protein	NA	403.46	1938.66	2.26	Up	0.94
POX02621	Hypothetical protein	NA	28.48	0.19	-7.20	Down	0.81
POX02623	Hypothetical protein	NA	395.04	15.68	-4.66	Down	0.94
POX02628	Hypothetical protein	NA	22.55	469.59	4.38	Up	0.94
POX02640	Hypothetical protein	NA	222.86	1188.25	2.41	Up	0.94
POX02646	Putative alpha-1,6-mannanase	GH76	513.54	46.40	-3.47	Down	0.94

POX02651	Hypothetical protein	NA	446.37	912.61	1.03	Up	0.83
POX02653	Hypothetical protein	NA	513.43	1675.05	1.71	Up	0.91
POX02655	Hypothetical protein	NA	149.86	410.22	1.45	Up	0.89
POX02668	Putative chitin glucanosyltransferase	GH16	205.29	527.97	1.36	Up	0.88
POX02677	Hypothetical protein	NA	74.34	29.02	-1.36	Down	0.80
POX02680	Hypothetical protein	NA	319.80	1398.61	2.13	Up	0.93
POX02682	Hypothetical protein	NA	261.96	81.43	-1.69	Down	0.89
POX02683	Hypothetical protein	NA	192.25	31.68	-2.60	Down	0.92
POX02685	Hypothetical protein	NA	206.75	625.88	1.60	Up	0.90
POX02687	Hypothetical protein	NA	191.51	69.17	-1.47	Down	0.87
POX02693	Hypothetical protein	NA	108.01	17.35	-2.64	Down	0.89
POX02694	Hypothetical protein	NA	172.09	13.08	-3.72	Down	0.92
POX02699	Hypothetical protein	NA	35.08	95.07	1.44	Up	0.83
POX02708	Hypothetical protein	NA	114.22	47.87	-1.25	Down	0.82
POX02711	Hypothetical protein	NA	140.62	43.39	-1.70	Down	0.87
POX02712	Hypothetical protein	NA	83.46	29.11	-1.52	Down	0.83
POX02718	Hypothetical protein	NA	5.59	104.96	4.23	Up	0.91
POX02721	Hypothetical protein	NA	1450.89	636.56	-1.19	Down	0.87
POX02730	Hypothetical protein	NA	366.36	1916.18	2.39	Up	0.94
POX02740	Endo-beta-1,4-glucanase	GH5	136.30	16.73	-3.03	Down	0.91
POX02741	Hypothetical protein	NA	59.59	192.97	1.70	Up	0.88
POX02746	Hypothetical protein	NA	62.05	17.14	-1.86	Down	0.83
POX02749	Hypothetical protein	NA	25.14	129.97	2.37	Up	0.90
POX02752	Hypothetical protein	NA	41.98	7.52	-2.48	Down	0.81
POX02755	Hypothetical protein	NA	74.54	200.99	1.43	Up	0.87
POX02758	Hypothetical protein	NA	59.28	12.16	-2.29	Down	0.84
POX02764	Hypothetical protein	NA	288.86	47.47	-2.61	Down	0.93
POX02768	Hypothetical protein	NA	112.89	36.48	-1.63	Down	0.86
POX02773	Hypothetical protein	NA	25.76	78.53	1.61	Up	0.83
POX02774	Hypothetical protein	NA	66.73	243.50	1.87	Up	0.90
POX02775	Hypothetical protein	NA	58.85	177.90	1.60	Up	0.88
POX02777	Hypothetical protein	NA	77.69	195.12	1.33	Up	0.86
POX02783	Hypothetical protein	NA	9.41	51.85	2.46	Up	0.83
POX02787	Hypothetical protein	NA	340.27	1703.99	2.32	Up	0.94
POX02804	Hypothetical protein	NA	83.18	216.84	1.38	Up	0.86
POX02815	Hypothetical protein	NA	30.97	120.11	1.96	Up	0.88
POX02821	Hypothetical protein	NA	211.83	827.57	1.97	Up	0.92
POX02822	Hypothetical protein	NA	56.36	200.55	1.83	Up	0.89

POX02834	Hypothetical protein	NA	75.63	626.64	3.05	Up	0.94
POX02837	Hypothetical protein	NA	618.21	31.26	-4.31	Down	0.95
POX02838	Hypothetical protein	NA	40.12	140.20	1.81	Up	0.88
POX02847	Hypothetical protein	NA	304.22	620.68	1.03	Up	0.83
POX02848	Putative chitinase	CBM18;CBM50;<br>GH18	114.05	5.25	-4.44	Down	0.91
POX02849	Hypothetical protein	NA	619.13	30.74	-4.33	Down	0.95
POX02853	Hypothetical protein	NA	1418.43	707.79	-1.00	Down	0.83
POX02856	Hypothetical protein	NA	47.36	122.29	1.37	Up	0.84
POX02860	Hypothetical protein	NA	1.38	110.17	6.32	Up	0.92
POX02861	Hypothetical protein	NA	94.40	6.83	-3.79	Down	0.90
POX02871	Hypothetical protein	NA	151.81	530.29	1.80	Up	0.91
POX02878	Hypothetical protein	NA	94.68	248.75	1.39	Up	0.87
POX02880	Hypothetical protein	NA	71.22	265.70	1.90	Up	0.90
POX02890	Hypothetical protein	NA	148.88	735.41	2.30	Up	0.93
POX02891	Hypothetical protein	NA	99.35	40.21	-1.30	Down	0.82
POX02896	Hypothetical protein	NA	429.36	1421.78	1.73	Up	0.92
POX02897	Hypothetical protein	NA	227.16	524.21	1.21	Up	0.86
POX02899	Hypothetical protein	NA	36.10	112.13	1.64	Up	0.86
POX02908	Hypothetical protein	NA	51.65	13.73	-1.91	Down	0.81
POX02909	Hypothetical protein	NA	175.01	55.97	-1.64	Down	0.88
POX02910	Hypothetical protein	GT41	40.25	102.38	1.35	Up	0.82
POX02918	Hypothetical protein	NA	1535.72	427.84	-1.84	Down	0.92
POX02926	Hypothetical protein	NA	40.67	105.72	1.38	Up	0.83
POX02943	Hypothetical protein	NA	375.39	98.43	-1.93	Down	0.91
POX02944	Hypothetical protein	NA	496.60	232.74	-1.09	Down	0.84
POX02953	Hypothetical protein	NA	68.90	205.83	1.58	Up	0.88
POX02959	Hypothetical protein	NA	202.69	92.83	-1.13	Down	0.83
POX02960	Hypothetical protein	NA	138.57	345.69	1.32	Up	0.87
POX02962	Hypothetical protein	NA	71.41	162.60	1.19	Up	0.83
POX02967	Hypothetical protein	NA	24.66	67.79	1.46	Up	0.80
POX02972	Hypothetical protein	NA	90.47	197.11	1.12	Up	0.82
POX02985	Hypothetical protein	NA	26.86	81.04	1.59	Up	0.83
POX02988	Hypothetical protein	NA	76.97	24.14	-1.67	Down	0.83
POX02992	Hypothetical protein	NA	295.74	60.71	-2.28	Down	0.92
POX02995	Hypothetical protein	CE1	58.30	151.60	1.38	Up	0.85
POX03007	Hypothetical protein	NA	60.68	8.87	-2.77	Down	0.86

POX03008	Hypothetical protein	NA	58.02	12.40	-2.23	Down	0.84
POX03009	Hypothetical protein	NA	132.98	58.79	-1.18	Down	0.82
POX03020	Hypothetical protein	NA	0.47	116.42	7.96	Up	0.95
POX03022	Hypothetical protein	NA	973.20	4042.50	2.05	Up	0.93
POX03025	Hypothetical protein	GH109	147.57	16.44	-3.17	Down	0.91
POX03029	Hypothetical protein	NA	73.95	174.94	1.24	Up	0.84
POX03033	Hypothetical protein	NA	0.47	38.84	6.38	Up	0.84
POX03051	Hypothetical protein	NA	157.74	65.14	-1.28	Down	0.84
POX03060	Hypothetical protein	NA	405.81	179.60	-1.18	Down	0.85
POX03068	Hypothetical protein	NA	212.58	52.55	-2.02	Down	0.90
POX03089	Hypothetical protein	NA	180.84	928.91	2.36	Up	0.94
POX03097	Hypothetical protein	NA	139.87	61.32	-1.19	Down	0.82
POX03104	Hypothetical protein	NA	24.06	281.69	3.55	Up	0.93
POX03105	Hypothetical protein	NA	74.01	13.82	-2.42	Down	0.87
POX03120	Hypothetical protein	NA	648.30	1418.93	1.13	Up	0.85
POX03129	Hypothetical protein	NA	816.52	298.90	-1.45	Down	0.89
POX03141	Hypothetical protein	NA	109.03	43.04	-1.34	Down	0.83
POX03142	Hypothetical protein	NA	24.93	103.67	2.06	Up	0.88
POX03159	Hypothetical protein	NA	35.88	123.70	1.79	Up	0.87
POX03183	Hypothetical protein	NA	351.74	112.73	-1.64	Down	0.90
POX03192	Hypothetical protein	NA	464.02	121.66	-1.93	Down	0.92
POX03222	Hypothetical protein	NA	156.08	71.39	-1.13	Down	0.81
POX03227	Hypothetical protein	NA	37.16	104.81	1.50	Up	0.84
POX03232	Hypothetical protein	NA	662.56	3118.53	2.23	Up	0.94
POX03233	Hypothetical protein	NA	110.51	43.07	-1.36	Down	0.83
POX03237	Hypothetical protein	NA	28.80	116.33	2.01	Up	0.88
POX03250	Hypothetical protein	NA	1.89	139.69	6.21	Up	0.93
POX03255	Hypothetical protein	NA	31.82	80.08	1.33	Up	0.80
POX03271	Hypothetical protein	NA	212.19	69.08	-1.62	Down	0.88
POX03272	Hypothetical protein	NA	6.86	46.37	2.76	Up	0.83
POX03285	Hypothetical protein	NA	100.94	42.66	-1.24	Down	0.81
POX03292	Hypothetical protein	NA	179.95	63.95	-1.49	Down	0.87
POX03297	Hypothetical protein	NA	36.77	92.88	1.34	Up	0.82
POX03300	Hypothetical protein	NA	47.50	155.99	1.72	Up	0.88
POX03301	Hypothetical protein	NA	30.38	91.29	1.59	Up	0.84
POX03308	Hypothetical protein	NA	666.35	250.12	-1.41	Down	0.89
POX03316	Hypothetical protein	NA	107.07	227.15	1.09	Up	0.82
POX03327	Hypothetical protein	CE10	51.78	156.85	1.60	Up	0.87

POX03331	Hypothetical protein	NA	442.36	1297.84	1.55	Up	0.90
POX03344	Hypothetical protein	NA	189.46	32.06	-2.56	Down	0.92
POX03347	Hypothetical protein	NA	24.72	954.58	5.27	Up	0.95
POX03354	Hypothetical protein	NA	56.64	14.89	-1.93	Down	0.82
POX03371	Hypothetical protein	NA	67.07	142.76	1.09	Up	0.80
POX03377	Hypothetical protein	NA	14.53	90.49	2.64	Up	0.88
POX03378	Hypothetical protein	NA	112.98	597.00	2.40	Up	0.93
POX03384	Hypothetical protein	NA	90.22	187.70	1.06	Up	0.81
POX03395	Hypothetical protein	NA	515.48	11.12	-5.53	Down	0.95
POX03396	Hypothetical protein	NA	83.18	6.31	-3.72	Down	0.89
POX03402	Hypothetical protein	NA	231.76	82.21	-1.50	Down	0.88
POX03413	Hypothetical protein	NA	445.57	62.57	-2.83	Down	0.94
POX03417	Hypothetical protein	NA	74.78	170.99	1.19	Up	0.83
POX03422	Hypothetical protein	NA	128.25	29.61	-2.11	Down	0.89
POX03429	Hypothetical protein	NA	142.11	52.12	-1.45	Down	0.86
POX03430	Putative endo-beta-1,4-xylanase	GH11	12941.12	705.12	-4.20	Down	0.95
POX03453	Hypothetical protein	NA	78.80	27.78	-1.50	Down	0.82
POX03468	Hypothetical protein	NA	219.82	50.62	-2.12	Down	0.91
POX03477	Hypothetical protein	NA	69.51	154.39	1.15	Up	0.82
POX03525	Hypothetical protein	NA	231.97	21.58	-3.43	Down	0.93
POX03534	Hypothetical protein	NA	105.81	21.02	-2.33	Down	0.89
POX03535	Hypothetical protein	CE12	13.43	56.24	2.07	Up	0.83
POX03540	Hypothetical protein	NA	370.07	46.87	-2.98	Down	0.93
POX03541	Hypothetical protein	NA	72.88	25.15	-1.53	Down	0.82
POX03547	Hypothetical protein	CE10;CE1	85.05	1.30	-6.04	Down	0.91
POX03563	Hypothetical protein	NA	71.21	208.37	1.55	Up	0.88
POX03568	Hypothetical protein	NA	66.55	164.27	1.30	Up	0.84
POX03571	Hypothetical protein	NA	449.92	1777.99	1.98	Up	0.93
POX03579	Hypothetical protein	NA	89.91	19.57	-2.20	Down	0.87
POX03582	Hypothetical protein	NA	70.92	407.43	2.52	Up	0.93
POX03584	Hypothetical protein	NA	346.98	796.07	1.20	Up	0.86
POX03588	Hypothetical protein	NA	18.69	58.39	1.64	Up	0.80
POX03598	Hypothetical protein	NA	450.04	85.08	-2.40	Down	0.93
POX03603	Hypothetical protein	NA	36.67	208.51	2.51	Up	0.92
POX03617	Hypothetical protein	NA	33.27	158.13	2.25	Up	0.90
POX03626	Hypothetical protein	NA	136.78	63.53	-1.11	Down	0.81
POX03627	Hypothetical protein	NA	14.55	146.10	3.33	Up	0.92
POX03628	Hypothetical protein	NA	91.75	20.09	-2.19	Down	0.87

POX03634	Hypothetical protein	NA	90.50	16.46	-2.46	Down	0.88
POX03636	Hypothetical protein	NA	250.49	0.24	-10.05	Down	0.98
POX03637	Hypothetical protein	NA	33.51	0.90	-5.22	Down	0.81
POX03665	Hypothetical protein	NA	56.49	272.59	2.27	Up	0.92
POX03666	Hypothetical protein	NA	66.54	189.73	1.51	Up	0.87
POX03674	Hypothetical protein	NA	42.55	124.54	1.55	Up	0.86
POX03696	Hypothetical protein	NA	68.08	147.30	1.11	Up	0.81
POX03698	Hypothetical protein	CE10	43.83	5.22	-3.07	Down	0.83
POX03699	Hypothetical protein	NA	223.62	60.26	-1.89	Down	0.90
POX03707	Hypothetical protein	NA	10.64	63.12	2.57	Up	0.86
POX03711	Putative acetyl xylan esterase	CE5	9432.99	393.80	-4.58	Down	0.96
POX03712	Hypothetical protein	NA	98.46	29.88	-1.72	Down	0.85
POX03728	Hypothetical protein	NA	114.04	38.38	-1.57	Down	0.85
POX03729	Hypothetical protein	NA	79.58	26.37	-1.59	Down	0.83
POX03730	Putative endopolygalacturonase	GH28	32.25	95.52	1.57	Up	0.84
POX03732	Putative UDP-GalNAc: alpha-1, 4-N-acetylgalactosaminyltransferase	GT32	26.43	117.72	2.15	Up	0.89
POX03733	Putative N-acetyl-1-D-myo-inosityl-2-amino-2-deoxy-alpha-D-glucopyranoside deacetylase	CE14	30.66	94.18	1.62	Up	0.85
POX03737	Hypothetical protein	NA	18.52	110.49	2.58	Up	0.89
POX03747	Hypothetical protein	NA	27.60	102.94	1.90	Up	0.87
POX03748	Hypothetical protein	NA	202.52	85.45	-1.24	Down	0.84
POX03749	Hypothetical protein	NA	65.39	312.12	2.25	Up	0.92
POX03758	Hypothetical protein	NA	403.44	1295.15	1.68	Up	0.91
POX03777	Hypothetical protein	NA	46.80	2.95	-3.99	Down	0.85
POX03782	Hypothetical protein	NA	1989.90	279.19	-2.83	Down	0.95
POX03784	Hypothetical protein	NA	623.29	2463.79	1.98	Up	0.93
POX03789	Hypothetical protein	NA	245.84	79.92	-1.62	Down	0.89
POX03793	Hypothetical protein	NA	142.82	32.72	-2.13	Down	0.89
POX03794	Hypothetical protein	NA	100.93	243.49	1.27	Up	0.85
POX03796	Hypothetical protein	NA	191.21	718.42	1.91	Up	0.92
POX03797	Hypothetical protein	NA	145.16	448.68	1.63	Up	0.90
POX03804	Hypothetical protein	NA	96.68	31.56	-1.62	Down	0.85
POX03813	Hypothetical protein	NA	272.61	80.98	-1.75	Down	0.90
POX03823	Hypothetical protein	NA	406.45	1532.91	1.92	Up	0.92
POX03824	Hypothetical protein	NA	25.35	74.62	1.56	Up	0.82

POX03825	Hypothetical protein	NA	56.07	177.07	1.66	Up	0.88
POX03826	Hypothetical protein	NA	68.00	9.31	-2.87	Down	0.87
POX03835	Hypothetical protein	NA	68.41	166.98	1.29	Up	0.84
POX03841	Hypothetical protein	NA	33.15	81.96	1.31	Up	0.80
POX03842	Hypothetical protein	NA	2.52	33.59	3.74	Up	0.80
POX03850	Hypothetical protein	NA	91.29	280.97	1.62	Up	0.89
POX03857	Hypothetical protein	NA	178.37	78.92	-1.18	Down	0.83
POX03860	Hypothetical protein	NA	96.98	29.47	-1.72	Down	0.85
POX03888	Hypothetical protein	NA	138.18	63.85	-1.11	Down	0.81
POX03889	Putative alpha-glucosidase	GH31	7.99	123.04	3.95	Up	0.91
POX03890	Hypothetical protein	NA	11.02	71.32	2.69	Up	0.87
POX03894	Hypothetical protein	NA	394.75	88.51	-2.16	Down	0.92
POX03896	Hypothetical protein	NA	170.57	61.21	-1.48	Down	0.87
POX03900	Hypothetical protein	NA	118.09	9.93	-3.57	Down	0.91
POX03902	Hypothetical protein	NA	115.84	1.69	-6.10	Down	0.92
POX03912	Hypothetical protein	NA	444.91	33.32	-3.74	Down	0.94
POX03913	Hypothetical protein	NA	202.14	22.43	-3.17	Down	0.92
POX03929	Hypothetical protein	NA	6.23	172.74	4.79	Up	0.93
POX03930	Hypothetical protein	NA	77.24	581.03	2.91	Up	0.94
POX03933	Hypothetical protein	NA	35.60	2.58	-3.78	Down	0.81
POX03935	Hypothetical protein	NA	306.71	11.00	-4.80	Down	0.94
POX03937	Hypothetical protein	NA	120.71	680.02	2.49	Up	0.93
POX03940	Hypothetical protein	NA	115.39	488.26	2.08	Up	0.92
POX03951	Hypothetical protein	NA	54.29	207.73	1.94	Up	0.90
POX03960	Hypothetical protein	NA	192.84	3.35	-5.85	Down	0.94
POX03963	Hypothetical protein	NA	38.57	114.00	1.56	Up	0.85
POX03966	Hypothetical protein	NA	56.69	15.50	-1.87	Down	0.82
POX03971	Hypothetical protein	NA	109.11	37.17	-1.55	Down	0.85
POX03988	Hypothetical protein	NA	134.43	497.96	1.89	Up	0.92
POX04011	Hypothetical protein	NA	60.31	136.61	1.18	Up	0.82
POX04016	Hypothetical protein	NA	425.98	1984.14	2.22	Up	0.94
POX04017	Hypothetical protein	NA	101.98	227.59	1.16	Up	0.84
POX04018	Hypothetical protein	NA	99.13	414.72	2.06	Up	0.92
POX04027	Hypothetical protein	NA	125.62	411.49	1.71	Up	0.90
POX04029	Hypothetical protein	NA	238.90	573.09	1.26	Up	0.87
POX04039	Hypothetical protein	NA	8.43	45.85	2.44	Up	0.82
POX04044	Hypothetical protein	NA	45.66	4.56	-3.32	Down	0.84
POX04047	Hypothetical protein	NA	49.35	110.94	1.17	Up	0.80

POX04048	Hypothetical protein	NA	308.80	1314.33	2.09	Up	0.93
POX04056	Hypothetical protein	NA	10.67	48.14	2.17	Up	0.81
POX04062	Hypothetical protein	NA	371.22	1718.37	2.21	Up	0.93
POX04063	Hypothetical protein	NA	35.83	130.66	1.87	Up	0.88
POX04068	Hypothetical protein	NA	130.33	40.30	-1.69	Down	0.87
POX04073	Hypothetical protein	NA	145.02	66.85	-1.12	Down	0.81
POX04075	Hypothetical protein	NA	341.57	1779.99	2.38	Up	0.94
POX04076	Hypothetical protein	NA	375.49	2008.65	2.42	Up	0.94
POX04079	Hypothetical protein	NA	67.70	19.06	-1.83	Down	0.83
POX04088	Hypothetical protein	NA	232.70	584.14	1.33	Up	0.88
POX04089	Hypothetical protein	NA	695.58	215.45	-1.69	Down	0.91
POX04093	Hypothetical protein	NA	54.52	316.80	2.54	Up	0.93
POX04101	Hypothetical protein	NA	119.41	281.63	1.24	Up	0.85
POX04114	Hypothetical protein	NA	36.34	175.51	2.27	Up	0.91
POX04118	Hypothetical protein	NA	19.81	61.87	1.64	Up	0.81
POX04136	Hypothetical protein	NA	598.71	3109.70	2.38	Up	0.94
POX04137	Putative endo-beta-1,4-glucanase	CBM1;GH5	66.67	489.44	2.88	Up	0.94
POX04143	Hypothetical protein	NA	68.18	288.55	2.08	Up	0.91
POX04149	Hypothetical protein	NA	283.02	120.84	-1.23	Down	0.85
POX04151	Hypothetical protein	NA	45.69	3.40	-3.75	Down	0.84
POX04160	Hypothetical protein	NA	50.09	118.65	1.24	Up	0.82
POX04161	Hypothetical protein	NA	376.85	812.75	1.11	Up	0.85
POX04166	Hypothetical protein	NA	19.80	64.29	1.70	Up	0.82
POX04181	Hypothetical protein	NA	312.11	1472.63	2.24	Up	0.94
POX04183	Hypothetical protein	NA	106.80	225.69	1.08	Up	0.82
POX04192	Hypothetical protein	NA	107.76	265.16	1.30	Up	0.86
POX04195	Hypothetical protein	NA	129.06	475.55	1.88	Up	0.91
POX04200	Hypothetical protein	NA	20.59	90.04	2.13	Up	0.87
POX04204	Hypothetical protein	NA	57.63	191.79	1.73	Up	0.89
POX04210	Hypothetical protein	NA	258.17	892.82	1.79	Up	0.92
POX04213	Hypothetical protein	NA	72.68	23.94	-1.60	Down	0.82
POX04214	Hypothetical protein	NA	2436.22	83.52	-4.87	Down	0.96
POX04215	Hypothetical protein	NA	869.63	26.54	-5.03	Down	0.95
POX04216	Hypothetical protein	NA	55.80	133.74	1.26	Up	0.83
POX04223	Hypothetical protein	NA	9.34	46.00	2.30	Up	0.81
POX04231	Hypothetical protein	NA	675.39	140.53	-2.26	Down	0.93
POX04232	Hypothetical protein	NA	260.84	47.35	-2.46	Down	0.92
POX04233	Hypothetical protein	NA	101.22	27.65	-1.87	Down	0.87

POX04234	Hypothetical protein	NA	19.51	63.23	1.70	Up	0.82
POX04239	Hypothetical protein	NA	36.93	91.59	1.31	Up	0.81
POX04242	Hypothetical protein	NA	25.08	94.90	1.92	Up	0.86
POX04257	Hypothetical protein	NA	46.85	2.30	-4.35	Down	0.85
POX04258	Hypothetical protein	NA	49.46	12.02	-2.04	Down	0.81
POX04265	Hypothetical protein	NA	83.81	34.20	-1.29	Down	0.80
POX04272	Hypothetical protein	NA	46.28	6.57	-2.82	Down	0.83
POX04276	Hypothetical protein	NA	684.83	318.00	-1.11	Down	0.85
POX04283	Hypothetical protein	NA	73.67	28.11	-1.39	Down	0.80
POX04292	Hypothetical protein	NA	150.51	69.31	-1.12	Down	0.81
POX04333	Hypothetical protein	NA	359.33	1623.32	2.18	Up	0.93
POX04334	Hypothetical protein	NA	279.84	795.26	1.51	Up	0.90
POX04337	Hypothetical protein	NA	244.86	85.87	-1.51	Down	0.88
POX04339	Hypothetical protein	NA	15.19	61.02	2.01	Up	0.83
POX04343	Hypothetical protein	NA	110.77	275.92	1.32	Up	0.86
POX04344	Hypothetical protein	NA	9.83	139.73	3.83	Up	0.92
POX04345	Hypothetical protein	NA	22.04	197.94	3.17	Up	0.92
POX04349	Hypothetical protein	NA	69.20	22.01	-1.65	Down	0.82
POX04356	Hypothetical protein	NA	67.31	191.59	1.51	Up	0.87
POX04363	Hypothetical protein	NA	70.74	19.90	-1.83	Down	0.84
POX04373	Hypothetical protein	NA	8.11	58.36	2.85	Up	0.85
POX04377	Putative rhamnogalacturonase	GH28	12.72	63.69	2.32	Up	0.85
POX04378	Hypothetical protein	NA	141.47	424.75	1.59	Up	0.90
POX04379	Hypothetical protein	NA	80.37	252.86	1.65	Up	0.89
POX04384	Hypothetical protein	NA	39.42	156.51	1.99	Up	0.89
POX04390	Putative beta-1,3-glucanosyltransglycosylase	CBM43;GH72	51.53	595.52	3.53	Up	0.94
POX04391	Hypothetical protein	NA	52.30	148.24	1.50	Up	0.86
POX04406	Hypothetical protein	NA	134.00	13.75	-3.29	Down	0.91
POX04407	Hypothetical protein	NA	253.81	34.75	-2.87	Down	0.93
POX04408	Hypothetical protein	NA	988.48	171.46	-2.53	Down	0.94
POX04411	Hypothetical protein	NA	3.72	50.46	3.76	Up	0.85
POX04413	Hypothetical protein	NA	113.06	504.88	2.16	Up	0.92
POX04414	Hypothetical protein	NA	275.48	729.84	1.41	Up	0.89
POX04446	Hypothetical protein	NA	2.50	67.17	4.75	Up	0.88
POX04448	Hypothetical protein	NA	371.22	1295.05	1.80	Up	0.92
POX04450	Hypothetical protein	NA	94.18	220.45	1.23	Up	0.85
POX04452	Hypothetical protein	NA	27.22	76.58	1.49	Up	0.82

POX04453	Hypothetical protein	NA	30.89	101.22	1.71	Up	0.86
POX04462	Hypothetical protein	NA	58.00	728.41	3.65	Up	0.95
POX04474	Hypothetical protein	NA	94.01	289.19	1.62	Up	0.89
POX04476	Hypothetical protein	NA	68.77	23.83	-1.53	Down	0.81
POX04478	Hypothetical protein	NA	291.63	9.37	-4.96	Down	0.94
POX04485	Hypothetical protein	NA	132.71	31.38	-2.08	Down	0.89
POX04499	Hypothetical protein	NA	87.47	33.64	-1.38	Down	0.82
POX04510	Hypothetical protein	NA	88.91	26.48	-1.75	Down	0.85
POX04521	Hypothetical protein	NA	79.83	14.28	-2.48	Down	0.87
POX04523	Hypothetical protein	NA	159.56	47.13	-1.76	Down	0.88
POX04524	Hypothetical protein	NA	612.58	184.98	-1.73	Down	0.91
POX04529	Hypothetical protein	NA	2096.63	69.53	-4.91	Down	0.96
POX04531	Hypothetical protein	NA	1159.59	217.28	-2.42	Down	0.94
POX04532	Carbohydrate binding domain-containing protein	NA	4.72	156.21	5.05	Up	0.93
POX04533	Hypothetical protein	NA	58.43	6.98	-3.07	Down	0.86
POX04534	Hypothetical protein	NA	114.05	21.61	-2.40	Down	0.89
POX04535	Hypothetical protein	NA	160.60	47.67	-1.75	Down	0.88
POX04539	Hypothetical protein	NA	216.03	47.37	-2.19	Down	0.91
POX04545	Hypothetical protein	NA	93.84	31.87	-1.56	Down	0.84
POX04549	Hypothetical protein	NA	24.67	106.85	2.11	Up	0.88
POX04564	Hypothetical protein	NA	69.75	193.28	1.47	Up	0.87
POX04565	Hypothetical protein	NA	56.05	166.02	1.57	Up	0.87
POX04566	Hypothetical protein	NA	31.85	89.17	1.49	Up	0.83
POX04574	Hypothetical protein	NA	63.64	22.50	-1.50	Down	0.80
POX04578	Hypothetical protein	NA	292.31	1.25	-7.87	Down	0.97
POX04590	Hypothetical protein	NA	57.55	16.81	-1.78	Down	0.81
POX04592	Hypothetical protein	NA	8.44	50.29	2.57	Up	0.83
POX04593	Hypothetical protein	NA	8.96	182.25	4.35	Up	0.93
POX04594	Hypothetical protein	NA	195.05	45.93	-2.09	Down	0.90
POX04599	Hypothetical protein	NA	12.86	62.99	2.29	Up	0.85
POX04606	Hypothetical protein	NA	12.81	1318.42	6.69	Up	0.97
POX04611	Hypothetical protein	NA	311.33	630.41	1.02	Up	0.83
POX04615	Hypothetical protein	NA	80.24	176.76	1.14	Up	0.82
POX04626	Putative beta-1,3-glucanosyltransglycosylase	CBM43;GH72	77.70	355.20	2.19	Up	0.92
POX04630	Hypothetical protein	NA	267.16	36.52	-2.87	Down	0.93

POX04640	Hypothetical protein	NA	193.73	92.21	-1.07	Down	0.81
POX04673	Hypothetical protein	NA	164.08	68.88	-1.25	Down	0.84
POX04681	Hypothetical protein	NA	121.93	40.72	-1.58	Down	0.86
POX04683	Hypothetical protein	NA	983.26	274.19	-1.84	Down	0.92
POX04686	Hypothetical protein	GH128	215.05	594.73	1.47	Up	0.89
POX04693	Hypothetical protein	NA	1.08	31.92	4.89	Up	0.81
POX04694	Hypothetical protein	NA	75.65	216.13	1.51	Up	0.88
POX04696	Hypothetical protein	NA	55.73	169.84	1.61	Up	0.88
POX04708	Hypothetical protein	NA	82.98	182.56	1.14	Up	0.82
POX04725	Hypothetical protein	NA	486.67	83.97	-2.53	Down	0.93
POX04726	Hypothetical protein	NA	300.93	106.32	-1.50	Down	0.88
POX04737	Hypothetical protein	NA	70.68	226.22	1.68	Up	0.89
POX04738	Hypothetical protein	NA	29.50	75.30	1.35	Up	0.80
POX04741	Hypothetical protein	NA	38.07	111.09	1.54	Up	0.85
POX04748	Hypothetical protein	NA	14.52	138.48	3.25	Up	0.91
POX04751	Hypothetical protein	NA	1529.53	320.09	-2.26	Down	0.94
POX04772	Hypothetical protein	NA	26.25	79.34	1.60	Up	0.83
POX04773	Hypothetical protein	NA	57.82	15.93	-1.86	Down	0.82
POX04775	Hypothetical protein	NA	75.13	168.69	1.17	Up	0.83
POX04778	Hypothetical protein	NA	9.53	67.24	2.82	Up	0.87
POX04779	Hypothetical protein	NA	6.63	93.37	3.82	Up	0.90
POX04781	Hypothetical protein	NA	11211.34	3770.05	-1.57	Down	0.91
POX04786	Cellobiohydrolase Cel6A	CBM1;GH6	3051.15	661.79	-2.20	Down	0.94
POX04795	Hypothetical protein	NA	17.90	72.70	2.02	Up	0.85
POX04798	Hypothetical protein	NA	142.87	65.75	-1.12	Down	0.81
POX04801	Hypothetical protein	NA	16.58	133.47	3.01	Up	0.91
POX04808	Hypothetical protein	NA	75.86	21.68	-1.81	Down	0.84
POX04824	Hypothetical protein	NA	84.66	28.43	-1.57	Down	0.83
POX04833	Hypothetical protein	NA	62.69	18.35	-1.77	Down	0.82
POX04834	Hypothetical protein	NA	88.00	5.60	-3.97	Down	0.90
POX04840	Hypothetical protein	NA	216.29	105.91	-1.03	Down	0.81
POX04850	Hypothetical protein	NA	270.46	1451.47	2.42	Up	0.94
POX04860	Hypothetical protein	NA	384.98	97.10	-1.99	Down	0.92
POX04865	Hypothetical protein	NA	55.63	148.92	1.42	Up	0.85
POX04866	Hypothetical protein	NA	5.03	113.01	4.49	Up	0.91
POX04867	Hypothetical protein	NA	8.90	214.78	4.59	Up	0.93
POX04868	Hypothetical protein	NA	7.84	43.20	2.46	Up	0.81
POX04869	Hypothetical protein	NA	5.98	52.92	3.14	Up	0.85

POX04872	Hypothetical protein	NA	853.03	177.22	-2.27	Down	0.93
POX04878	Hypothetical protein	NA	33.41	98.08	1.55	Up	0.84
POX04895	Putative alpha-mannosidase	GH92	405.77	34.19	-3.57	Down	0.94
POX04909	Hypothetical protein	NA	29.78	92.91	1.64	Up	0.85
POX04917	Hypothetical protein	NA	159.57	50.17	-1.67	Down	0.88
POX04918	Hypothetical protein	NA	18.54	105.41	2.51	Up	0.89
POX04920	Putative pectin lyase	PL1	497.22	23.97	-4.37	Down	0.94
POX04922	Hypothetical protein	NA	13.26	51.31	1.95	Up	0.81
POX04925	Hypothetical protein	NA	205.64	528.22	1.36	Up	0.88
POX04928	Hypothetical protein	NA	151.50	57.44	-1.40	Down	0.85
POX04929	Hypothetical protein	NA	158.21	76.52	-1.05	Down	0.80
POX04933	Hypothetical protein	NA	140.15	40.81	-1.78	Down	0.88
POX04934	Hypothetical protein	NA	202.37	46.37	-2.13	Down	0.91
POX04938	Putative glycogen branching enzyme	CBM48;GH13	249.99	93.79	-1.41	Down	0.87
POX04940	Hypothetical protein	NA	739.00	1943.75	1.40	Up	0.89
POX04941	Hypothetical protein	NA	140.15	467.58	1.74	Up	0.91
POX04943	Hypothetical protein	NA	164.74	511.94	1.64	Up	0.90
POX04947	Hypothetical protein	NA	106.80	477.43	2.16	Up	0.92
POX04959	Hypothetical protein	NA	39.35	146.53	1.90	Up	0.89
POX04981	Hypothetical protein	NA	204.38	83.24	-1.30	Down	0.85
POX04985	Hypothetical protein	NA	77.26	163.07	1.08	Up	0.81
POX04987	Hypothetical protein	NA	92.78	190.99	1.04	Up	0.81
POX04995	Putative beta-xylosidase/alpha-L-arabinofuranosidase	GH43	124.79	24.16	-2.37	Down	0.90
POX05004	Hypothetical protein	NA	32.09	121.34	1.92	Up	0.88
POX05007	Hypothetical protein	NA	63.62	4402.02	6.11	Up	0.96
POX05009	Hypothetical protein	NA	82.92	26.31	-1.66	Down	0.84
POX05015	Hypothetical protein	NA	68.32	20.72	-1.72	Down	0.83
POX05027	Hypothetical protein	NA	18.25	70.30	1.95	Up	0.84
POX05048	Hypothetical protein	NA	369.44	139.15	-1.41	Down	0.88
POX05050	Hypothetical protein	NA	51.92	14.57	-1.83	Down	0.80
POX05053	Hypothetical protein	NA	39.91	4.75	-3.07	Down	0.82
POX05055	Hypothetical protein	NA	269.42	53.54	-2.33	Down	0.92
POX05058	Hypothetical protein	NA	49.83	142.84	1.52	Up	0.86
POX05059	Hypothetical protein	NA	35.71	163.91	2.20	Up	0.90
POX05061	Hypothetical protein	NA	72.98	16.63	-2.13	Down	0.85
POX05066	Hypothetical protein	NA	32.43	96.77	1.58	Up	0.84

POX05069	Hypothetical protein	NA	100.43	40.08	-1.33	Down	0.82
POX05076	Hypothetical protein	NA	48.05	8.61	-2.48	Down	0.83
POX05078	Hypothetical protein	GH109	66.94	18.21	-1.88	Down	0.83
POX05081	Hypothetical protein	NA	150.01	66.31	-1.18	Down	0.82
POX05090	Hypothetical protein	NA	48.84	11.24	-2.12	Down	0.81
POX05091	Hypothetical protein	NA	41.08	8.40	-2.29	Down	0.80
POX05097	Hypothetical protein	NA	134.90	63.28	-1.09	Down	0.80
POX05110	Hypothetical protein	NA	10.48	54.48	2.38	Up	0.84
POX05112	Hypothetical protein	NA	278.22	1139.28	2.03	Up	0.93
POX05113	Hypothetical protein	NA	109.23	34.21	-1.67	Down	0.86
POX05120	Hypothetical protein	NA	112.62	42.22	-1.42	Down	0.84
POX05122	Hypothetical protein	NA	120.93	355.51	1.56	Up	0.89
POX05129	Hypothetical protein	NA	36.68	4.67	-2.97	Down	0.80
POX05131	Hypothetical protein	NA	66.66	301.84	2.18	Up	0.92
POX05132	Putative cutinase	CE5	11896.19	79.73	-7.22	Down	0.98
POX05137	Hypothetical protein	NA	107.81	28.35	-1.93	Down	0.87
POX05139	Hypothetical protein	NA	84.10	30.78	-1.45	Down	0.82
POX05145	Hypothetical protein	NA	102.50	14.91	-2.78	Down	0.89
POX05170	Hypothetical protein	NA	28.70	92.10	1.68	Up	0.85
POX05177	Hypothetical protein	NA	51.43	178.16	1.79	Up	0.89
POX05180	Hypothetical protein	NA	514.85	61.41	-3.07	Down	0.94
POX05190	Hypothetical protein	NA	2.85	46.60	4.03	Up	0.85
POX05193	Hypothetical protein	NA	28.35	115.63	2.03	Up	0.88
POX05205	Hypothetical protein	NA	27.77	89.30	1.69	Up	0.85
POX05232	Hypothetical protein	NA	114.56	30.54	-1.91	Down	0.87
POX05233	Hypothetical protein	NA	109.93	37.65	-1.55	Down	0.85
POX05238	Hypothetical protein	NA	45.56	156.76	1.78	Up	0.88
POX05256	Hypothetical protein	NA	364.23	121.64	-1.58	Down	0.89
POX05262	Hypothetical protein	NA	356.35	24.37	-3.87	Down	0.94
POX05263	Hypothetical protein	CE9	105.02	9.66	-3.44	Down	0.90
POX05269	Hypothetical protein	NA	927.47	215.29	-2.11	Down	0.93
POX05271	Hypothetical protein	NA	188.51	62.39	-1.60	Down	0.88
POX05272	Hypothetical protein	NA	90.38	27.83	-1.70	Down	0.85
POX05275	Hypothetical protein	NA	174.01	43.63	-2.00	Down	0.90
POX05276	Hypothetical protein	NA	116.69	32.43	-1.85	Down	0.87
POX05277	Hypothetical protein	NA	59.57	15.94	-1.90	Down	0.82
POX05279	Hypothetical protein	NA	19.00	62.01	1.71	Up	0.82
POX05283	Hypothetical protein	NA	418.05	864.48	1.05	Up	0.84

POX05290	Hypothetical protein	NA	463.59	186.30	-1.32	Down	0.87
POX05297	Hypothetical protein	NA	37.80	90.56	1.26	Up	0.80
POX05299	Small GTPase superfamily	NA	23.39	147.65	2.66	Up	0.91
POX05301	Hypothetical protein	NA	130.28	27.88	-2.22	Down	0.89
POX05306	Hypothetical protein	NA	36.22	636.44	4.14	Up	0.95
POX05307	Hypothetical protein	NA	2.67	84.00	4.98	Up	0.90
POX05314	Hypothetical protein	NA	4.06	94.80	4.54	Up	0.90
POX05315	Hypothetical protein	NA	39.87	123.50	1.63	Up	0.86
POX05320	Hypothetical protein	NA	163.07	53.71	-1.60	Down	0.87
POX05321	Hypothetical protein	NA	188.46	61.34	-1.62	Down	0.88
POX05322	Hypothetical protein	NA	343.87	124.61	-1.46	Down	0.88
POX05335	Hypothetical protein	NA	61.01	18.71	-1.71	Down	0.81
POX05339	Hypothetical protein	NA	84.35	31.78	-1.41	Down	0.82
POX05343	Hypothetical protein	NA	81.94	23.95	-1.77	Down	0.84
POX05358	Hypothetical protein	NA	64.84	5.26	-3.62	Down	0.87
POX05378	Hypothetical protein	NA	1460.86	409.15	-1.84	Down	0.92
POX05386	Hypothetical protein	NA	76.04	166.57	1.13	Up	0.82
POX05391	Hypothetical protein	NA	148.20	40.61	-1.87	Down	0.89
POX05396	Hypothetical protein	NA	157.40	378.49	1.27	Up	0.86
POX05405	Hypothetical protein	NA	884.72	284.60	-1.64	Down	0.91
POX05406	Hypothetical protein	NA	66.19	156.13	1.24	Up	0.83
POX05407	Hypothetical protein	NA	261.19	123.39	-1.08	Down	0.83
POX05422	Hypothetical protein	NA	273.67	1278.29	2.22	Up	0.93
POX05433	Hypothetical protein	NA	117.52	49.25	-1.25	Down	0.82
POX05437	Hypothetical protein	NA	70.81	231.53	1.71	Up	0.89
POX05443	Hypothetical protein	NA	116.51	42.63	-1.45	Down	0.85
POX05451	Hypothetical protein	GH125	282.22	134.14	-1.07	Down	0.83
POX05459	Hypothetical protein	NA	24.17	116.46	2.27	Up	0.89
POX05464	Hypothetical protein	NA	79.25	9.72	-3.03	Down	0.88
POX05468	Hypothetical protein	NA	227.10	483.17	1.09	Up	0.84
POX05470	Hypothetical protein	NA	61.28	5.82	-3.40	Down	0.87
POX05482	Hypothetical protein	NA	88.03	776.34	3.14	Up	0.94
POX05485	Hypothetical protein	NA	77.83	18.62	-2.06	Down	0.86
POX05486	Hypothetical protein	NA	29.22	117.19	2.00	Up	0.88
POX05497	Hypothetical protein	NA	408.16	2381.53	2.54	Up	0.94
POX05498	Hypothetical protein	NA	119.95	52.11	-1.20	Down	0.81
POX05512	Hypothetical protein	NA	23.45	3653.23	7.28	Up	0.98
POX05515	Hypothetical protein	NA	20.12	210.11	3.38	Up	0.93

POX05529	Hypothetical protein	NA	209.88	11.08	-4.24	Down	0.93
POX05530	Hypothetical protein	NA	56.17	17.98	-1.64	Down	0.80
POX05535	Hypothetical protein	NA	98.97	23.54	-2.07	Down	0.87
POX05540	Putative alpha-L-arabinofuranosidase	GH62	1260.66	51.92	-4.60	Down	0.95
POX05545	Hypothetical protein	NA	48.85	173.12	1.83	Up	0.89
POX05553	Hypothetical protein	NA	274.15	87.98	-1.64	Down	0.89
POX05561	Hypothetical protein	NA	58.81	135.14	1.20	Up	0.82
POX05569	Hypothetical protein	NA	4173.30	38.84	-6.75	Down	0.97
POX05570	Endo-beta-1,4-glucanase Cel45A	CBM1;GH45	1279.21	610.98	-1.07	Down	0.84
POX05572	Hypothetical protein	NA	14.62	196.39	3.75	Up	0.93
POX05575	Hypothetical protein	NA	211.48	3569.46	4.08	Up	0.95
POX05577	Hypothetical protein	NA	1334.09	30.39	-5.46	Down	0.96
POX05578	Hypothetical protein	NA	15787.09	28.14	-9.13	Down	1.00
POX05580	Putative polygalacturonase	GH28	13.28	418.93	4.98	Up	0.95
POX05589	Hypothetical protein	NA	18.82	80.25	2.09	Up	0.86
POX05593	Hypothetical protein	NA	88.78	206.44	1.22	Up	0.84
POX05598	Hypothetical protein	NA	36.50	3.87	-3.24	Down	0.81
POX05601	Hypothetical protein	NA	114.65	35.55	-1.69	Down	0.86
POX05604	Putative beta-1, 3-glucanosyltransglycosylase	GH72	588.55	197.58	-1.57	Down	0.90
POX05605	Hypothetical protein	NA	64.88	161.95	1.32	Up	0.85
POX05641	Hypothetical protein	NA	32.30	172.60	2.42	Up	0.91
POX05654	Hypothetical protein	NA	33.39	102.82	1.62	Up	0.85
POX05660	Hypothetical protein	NA	115.93	623.26	2.43	Up	0.93
POX05677	Hypothetical protein	NA	179.58	485.14	1.43	Up	0.89
POX05679	Hypothetical protein	NA	63.27	152.19	1.27	Up	0.84
POX05690	Hypothetical protein	NA	146.17	65.25	-1.16	Down	0.82
POX05692	Hypothetical protein	NA	183.77	60.85	-1.59	Down	0.88
POX05702	Hypothetical protein	NA	119.40	2.75	-5.44	Down	0.92
POX05703	Hypothetical protein	NA	325.41	132.76	-1.29	Down	0.86
POX05707	Hypothetical protein	NA	43.13	6.69	-2.69	Down	0.82
POX05726	Hypothetical protein	NA	72.69	19.08	-1.93	Down	0.84
POX05728	Hypothetical protein	NA	322.64	70.60	-2.19	Down	0.92
POX05729	Hypothetical protein	NA	170.43	373.66	1.13	Up	0.84
POX05732	Hypothetical protein	NA	36.03	213.01	2.56	Up	0.92
POX05735	Hypothetical protein	NA	116.88	23.45	-2.32	Down	0.89
POX05741	Hypothetical protein	NA	101.24	11.52	-3.14	Down	0.90
POX05747	Hypothetical protein	NA	32.83	104.61	1.67	Up	0.86

POX05748	beta-oligosaccharyltransferase	GT66	25.64	94.37	1.88	Up	0.86
POX05750	Hypothetical protein	NA	95.71	442.29	2.21	Up	0.92
POX05754	Hypothetical protein	NA	68.64	18.08	-1.92	Down	0.84
POX05769	Hypothetical protein	NA	18.75	81.83	2.13	Up	0.86
POX05772	Hypothetical protein	NA	68.12	225.84	1.73	Up	0.89
POX05778	Hypothetical protein	NA	245.43	494.06	1.01	Up	0.82
POX05779	Hypothetical protein	NA	42.90	98.92	1.21	Up	0.80
POX05783	Hypothetical protein	NA	134.92	37.30	-1.85	Down	0.88
POX05787	Hypothetical protein	NA	104.45	45.71	-1.19	Down	0.80
POX05796	Hypothetical protein	NA	104.51	33.99	-1.62	Down	0.85
POX05799	Hypothetical protein	NA	32.05	111.64	1.80	Up	0.87
POX05804	Hypothetical protein	NA	14.12	68.88	2.29	Up	0.86
POX05807	Hypothetical protein	NA	89.25	184.28	1.05	Up	0.81
POX05818	Hypothetical protein	NA	171.34	26.11	-2.71	Down	0.91
POX05832	Hypothetical protein	NA	2.80	38.17	3.77	Up	0.82
POX05835	Hypothetical protein	NA	298.25	7.40	-5.33	Down	0.94
POX05837	Hypothetical protein	NA	29.24	156.65	2.42	Up	0.91
POX05838	Hypothetical protein	NA	19.95	63.91	1.68	Up	0.82
POX05844	Hypothetical protein	NA	109.34	532.44	2.28	Up	0.93
POX05869	Hypothetical protein	NA	87.74	25.03	-1.81	Down	0.85
POX05873	Hypothetical protein	NA	41.01	104.36	1.35	Up	0.83
POX05880	Ribosomal protein L1	NA	240.25	915.39	1.93	Up	0.92
POX05881	Hypothetical protein	NA	110.66	45.57	-1.28	Down	0.82
POX05887	Hypothetical protein	NA	131.74	22.23	-2.57	Down	0.90
POX05888	Hypothetical protein	NA	136.61	6.82	-4.32	Down	0.92
POX05897	Hypothetical protein	NA	52.17	298.83	2.52	Up	0.93
POX05898	Hypothetical protein	NA	323.70	111.76	-1.53	Down	0.89
POX05900	Hypothetical protein	NA	120.09	355.02	1.56	Up	0.89
POX05905	Hypothetical protein	NA	182.43	69.66	-1.39	Down	0.86
POX05907	Hypothetical protein	NA	77.86	12.01	-2.70	Down	0.88
POX05908	Hypothetical protein	NA	4.71	39.00	3.05	Up	0.81
POX05911	Hypothetical protein	NA	30.54	97.50	1.67	Up	0.85
POX05912	Hypothetical protein	NA	129.11	58.76	-1.14	Down	0.81
POX05913	Hypothetical protein	NA	55.66	6.90	-3.01	Down	0.85
POX05915	Cellodextrin transporter cdt-d	NA	3033.06	1426.36	-1.09	Down	0.85
POX05916	Putative endo-beta-1,4-xylanase	GH10	96.99	443.77	2.19	Up	0.92
POX05917	Hypothetical protein	NA	19.11	74.18	1.96	Up	0.85
POX05932	Hypothetical protein	NA	51.76	115.07	1.15	Up	0.80

POX05933	Hypothetical protein	NA	171.20	407.03	1.25	Up	0.86
POX05936	Hypothetical protein	NA	140.07	391.32	1.48	Up	0.89
POX05943	Hypothetical protein	NA	75.64	265.65	1.81	Up	0.90
POX05952	Hypothetical protein	NA	77.13	214.24	1.47	Up	0.87
POX05956	Hypothetical protein	NA	123.33	1020.94	3.05	Up	0.95
POX05959	Hypothetical protein	NA	125.37	54.95	-1.19	Down	0.82
POX05962	Hypothetical protein	NA	120.57	27.46	-2.13	Down	0.89
POX05963	Hypothetical protein	NA	48.29	116.34	1.27	Up	0.82
POX05966	Hypothetical protein	NA	306.56	65.07	-2.24	Down	0.92
POX05981	Hypothetical protein	NA	7.64	46.79	2.61	Up	0.83
POX05983	Hypothetical protein	NA	53.95	126.07	1.22	Up	0.82
POX05985	Hypothetical protein	NA	137.19	46.99	-1.55	Down	0.86
POX05987	Hypothetical protein	NA	63.99	0.95	-6.07	Down	0.89
POX05989	Hypothetical protein	NA	385.13	992.41	1.37	Up	0.89
POX05998	Hypothetical protein	NA	451.41	178.14	-1.34	Down	0.88
POX05999	Hypothetical protein	NA	205.30	88.33	-1.22	Down	0.84
POX06017	Hypothetical protein	NA	114.97	47.65	-1.27	Down	0.82
POX06025	Hypothetical protein	NA	61.24	5.29	-3.53	Down	0.87
POX06028	Hypothetical protein	NA	387.91	1966.89	2.34	Up	0.94
POX06033	Hypothetical protein	NA	19.79	154.45	2.96	Up	0.91
POX06034	Hypothetical protein	NA	378.34	923.46	1.29	Up	0.88
POX06036	Hypothetical protein	NA	1905.15	4123.69	1.11	Up	0.86
POX06044	Hypothetical protein	NA	121.64	331.61	1.45	Up	0.88
POX06052	Hypothetical protein	NA	104.81	32.31	-1.70	Down	0.86
POX06054	Hypothetical protein	NA	125.58	8.96	-3.81	Down	0.91
POX06064	Hypothetical protein	NA	84.11	280.33	1.74	Up	0.90
POX06067	Putative alpha-mannosidase	GH92	108.06	4.44	-4.61	Down	0.91
POX06069	Hypothetical protein	NA	135.41	32.72	-2.05	Down	0.89
POX06073	Hypothetical protein	NA	87.74	36.53	-1.26	Down	0.80
POX06079	beta-glucosidase	GH1	1327.08	580.30	-1.19	Down	0.87
POX06090	Hypothetical protein	NA	172.87	365.25	1.08	Up	0.83
POX06092	Hypothetical protein	NA	106.93	329.08	1.62	Up	0.89
POX06098	Hypothetical protein	NA	44.81	2.13	-4.40	Down	0.84
POX06099	Hypothetical protein	NA	288.31	102.33	-1.49	Down	0.88
POX06101	Hypothetical protein	NA	121.43	50.23	-1.27	Down	0.82
POX06102	Hypothetical protein	NA	69.61	16.80	-2.05	Down	0.85
POX06103	Hypothetical protein	NA	63.55	15.58	-2.03	Down	0.84
POX06104	Hypothetical protein	NA	136.19	13.74	-3.31	Down	0.91

POX06117	Hypothetical protein	NA	78.66	251.86	1.68	Up	0.89
POX06123	Hypothetical protein	NA	135.48	12.38	-3.45	Down	0.91
POX06128	Hypothetical protein	NA	86.18	347.31	2.01	Up	0.91
POX06145	Hypothetical protein	NA	1433.28	10408.15	2.86	Up	0.95
POX06146	Hypothetical protein	NA	665.88	4645.62	2.80	Up	0.95
POX06147	Endo-beta-1,4-glucanase Cel5A	CBM1;GH5	727.51	348.56	-1.06	Down	0.84
POX06151	Hypothetical protein	NA	447.76	104.48	-2.10	Down	0.92
POX06153	Hypothetical protein	NA	751.26	110.58	-2.76	Down	0.94
POX06155	Hypothetical protein	AA1	81.13	11.61	-2.80	Down	0.88
POX06159	Hypothetical protein	NA	36.63	103.77	1.50	Up	0.84
POX06162	Hypothetical protein	NA	257.42	1446.94	2.49	Up	0.94
POX06165	Hypothetical protein	NA	27.54	117.77	2.10	Up	0.88
POX06166	Hypothetical protein	NA	7.51	130.61	4.12	Up	0.92
POX06167	Hypothetical protein	NA	63.99	306.12	2.26	Up	0.92
POX06168	Hypothetical protein	NA	15.96	1578.07	6.63	Up	0.97
POX06176	Hypothetical protein	NA	295.17	716.41	1.28	Up	0.87
POX06194	Hypothetical protein	NA	50.90	125.90	1.31	Up	0.83
POX06199	Hypothetical protein	NA	18.19	81.09	2.16	Up	0.86
POX06200	Hypothetical protein	CE1	345.15	84.45	-2.03	Down	0.92
POX06203	Hypothetical protein	CE10	46.04	4.17	-3.46	Down	0.84
POX06204	Hypothetical protein	NA	141.07	911.16	2.69	Up	0.94
POX06205	Hypothetical protein	NA	98.94	9.80	-3.34	Down	0.90
POX06207	Hypothetical protein	NA	132.05	21.79	-2.60	Down	0.90
POX06221	Hypothetical protein	NA	52.16	257.70	2.30	Up	0.92
POX06223	Hypothetical protein	NA	1.31	40.49	4.95	Up	0.84
POX06224	Hypothetical protein	NA	15.60	144.84	3.21	Up	0.91
POX06231	Hypothetical protein	NA	2055.31	528.55	-1.96	Down	0.93
POX06232	Hypothetical protein	NA	27.27	73.09	1.42	Up	0.81
POX06236	Hypothetical protein	NA	98.58	401.23	2.03	Up	0.92
POX06241	Putative chitin glucanosyltransferase	GH16	50.44	502.89	3.32	Up	0.94
POX06242	Putative alpha-glucosidase	GH31	194.41	50.71	-1.94	Down	0.90
POX06245	Hypothetical protein	NA	75.25	439.70	2.55	Up	0.93
POX06262	Hypothetical protein	NA	226.69	978.79	2.11	Up	0.93
POX06270	Hypothetical protein	NA	79.46	340.02	2.10	Up	0.92
POX06285	Hypothetical protein	NA	13.78	180.74	3.71	Up	0.92
POX06286	Hypothetical protein	NA	59.10	15.19	-1.96	Down	0.83
POX06288	Hypothetical protein	NA	8.59	49.49	2.53	Up	0.83
POX06290	Hypothetical protein	NA	74.76	268.82	1.85	Up	0.90

POX06301	Putative alpha-mannosidase	GH47	1533.37	74.71	-4.36	Down	0.95
POX06312	Hypothetical protein	NA	23.43	157.93	2.75	Up	0.91
POX06317	Hypothetical protein	NA	12.60	81.93	2.70	Up	0.88
POX06329	Ribosomal protein L22/L17	NA	140.03	537.55	1.94	Up	0.92
POX06337	Hypothetical protein	NA	9.71	48.78	2.33	Up	0.82
POX06346	Hypothetical protein	NA	72.57	236.85	1.71	Up	0.89
POX06348	Hypothetical protein	NA	137.73	475.66	1.79	Up	0.91
POX06353	Hypothetical protein	NA	281.81	37.58	-2.91	Down	0.93
POX06358	Hypothetical protein	NA	123.66	260.24	1.07	Up	0.82
POX06361	Hypothetical protein	NA	24.10	73.83	1.61	Up	0.83
POX06373	Hypothetical protein	NA	40.96	6.11	-2.75	Down	0.81
POX06377	Hypothetical protein	NA	41.77	137.81	1.72	Up	0.87
POX06379	Hypothetical protein	NA	7.60	123.23	4.02	Up	0.91
POX06380	SUN domain-containing protein	GH132	28.31	82.44	1.54	Up	0.83
POX06390	Hypothetical protein	NA	17.49	74.31	2.09	Up	0.85
POX06391	Hypothetical protein	NA	46.25	373.43	3.01	Up	0.94
POX06394	Histone H4	NA	760.06	2688.26	1.82	Up	0.92
POX06396	Hypothetical protein	NA	22.45	82.16	1.87	Up	0.85
POX06398	Hypothetical protein	NA	14.29	103.90	2.86	Up	0.90
POX06399	Hypothetical protein	NA	57.85	343.07	2.57	Up	0.93
POX06410	Hypothetical protein	NA	25.83	84.58	1.71	Up	0.84
POX06415	Hypothetical protein	NA	25.87	71.07	1.46	Up	0.81
POX06417	Hypothetical protein	NA	103.74	253.72	1.29	Up	0.86
POX06424	Hypothetical protein	NA	75.24	19.63	-1.94	Down	0.85
POX06431	Hypothetical protein	NA	261.68	920.04	1.81	Up	0.92
POX06446	Hypothetical protein	NA	75.35	1384.28	4.20	Up	0.95
POX06450	Hypothetical protein	NA	340.58	48.52	-2.81	Down	0.93
POX06452	Hypothetical protein	NA	67.97	184.79	1.44	Up	0.87
POX06455	Hypothetical protein	NA	31.11	102.63	1.72	Up	0.86
POX06459	Hypothetical protein	NA	68.38	175.22	1.36	Up	0.85
POX06464	Hypothetical protein	NA	50.79	7.92	-2.68	Down	0.84
POX06465	Hypothetical protein	NA	126.29	1.55	-6.35	Down	0.93
POX06466	Hypothetical protein	NA	83.36	292.72	1.81	Up	0.90
POX06469	Hypothetical protein	NA	14.06	50.36	1.84	Up	0.80
POX06478	Hypothetical protein	NA	1.28	141.74	6.79	Up	0.94
POX06491	Hypothetical protein	NA	13.95	51.66	1.89	Up	0.81
POX06500	Putative glycogen/starch phosphorylase	GT35	1413.55	236.27	-2.58	Down	0.94
POX06508	Hypothetical protein	NA	193.38	15.09	-3.68	Down	0.93

POX06516	Hypothetical protein	NA	890.73	2886.32	1.70	Up	0.92
POX06530	Hypothetical protein	NA	137.69	357.81	1.38	Up	0.88
POX06534	Hypothetical protein	NA	70.56	6.57	-3.42	Down	0.88
POX06551	Putative alpha-1,6-mannosyltransferase	GT32	19.91	89.34	2.17	Up	0.87
POX06554	Hypothetical protein	NA	14.18	61.48	2.12	Up	0.84
POX06563	Hypothetical protein	NA	137.77	52.46	-1.39	Down	0.85
POX06566	Hypothetical protein	NA	64.39	138.47	1.10	Up	0.81
POX06571	beta-xylosidase	GH3	97.67	41.01	-1.25	Down	0.81
POX06574	Hypothetical protein	NA	5.09	52.99	3.38	Up	0.85
POX06575	Hypothetical protein	NA	62.75	164.50	1.39	Up	0.85
POX06580	Hypothetical protein	NA	288.94	746.43	1.37	Up	0.88
POX06582	Putative GDP-Man: alpha-D-mannose-a-(1, 6)-phosphatidyl myo-inositol monomannosyltransferase	GT4;GT5	50.35	11.32	-2.15	Down	0.82
POX06590	Hypothetical protein	NA	126.98	277.13	1.13	Up	0.83
POX06595	Hypothetical protein	NA	702.34	34.92	-4.33	Down	0.95
POX06599	Putative alpha-L-arabinofuranosidase	CBM1;GH62	3463.29	419.94	-3.04	Down	0.95
POX06600	Putative alpha-L-arabinofuranosidase	CBM1;GH43	914.69	45.31	-4.34	Down	0.95
POX06601	Putative endo-beta-1, 4-xylanase	CBM1;GH30	17.56	73.71	2.07	Up	0.85
POX06602	Hypothetical protein	AA7	29.24	264.26	3.18	Up	0.93
POX06604	Hypothetical protein	NA	258.96	23.61	-3.46	Down	0.93
POX06605	Hypothetical protein	NA	69.42	9.98	-2.80	Down	0.87
POX06606	Hypothetical protein	NA	513.19	60.66	-3.08	Down	0.94
POX06607	Hypothetical protein	NA	1091.28	109.08	-3.32	Down	0.95
POX06608	Hypothetical protein	NA	155.69	17.94	-3.12	Down	0.92
POX06609	Hypothetical protein	NA	21.18	84.94	2.00	Up	0.86
POX06610	Hypothetical protein	NA	1007.86	54.89	-4.20	Down	0.95
POX06611	Putative rhamnogalacturonan lyase	PL4	33.25	2.67	-3.64	Down	0.80
POX06615	Hypothetical protein	NA	73.09	19.82	-1.88	Down	0.84
POX06621	Hypothetical protein	NA	1284.51	276.27	-2.22	Down	0.93
POX06622	Hypothetical protein	NA	81.50	25.22	-1.69	Down	0.84
POX06623	Hypothetical protein	NA	421.62	198.09	-1.09	Down	0.84
POX06627	Hypothetical protein	NA	77.74	213.53	1.46	Up	0.87
POX06628	Hypothetical protein	NA	17.03	78.95	2.21	Up	0.86
POX06641	Hypothetical protein	NA	361.12	18.22	-4.31	Down	0.94

POX06642	Hypothetical protein	NA	37.19	118.32	1.67	Up	0.86
POX06646	Hypothetical protein	NA	330.90	668.81	1.02	Up	0.83
POX06654	Hypothetical protein	NA	205.13	559.72	1.45	Up	0.89
POX06664	Hypothetical protein	NA	54.17	144.53	1.42	Up	0.85
POX06682	Hypothetical protein	NA	114.79	19.78	-2.54	Down	0.90
POX06689	Putative endo-beta-1,4-galactanase	GH53	428.89	120.46	-1.83	Down	0.91
POX06696	Hypothetical protein	NA	337.11	1636.36	2.28	Up	0.94
POX06699	Hypothetical protein	NA	11.61	45.95	1.99	Up	0.80
POX06702	Hypothetical protein	NA	2143.15	4396.93	1.04	Up	0.84
POX06705	Hypothetical protein	NA	305.59	1428.17	2.22	Up	0.93
POX06738	Hypothetical protein	NA	1010.69	121.66	-3.05	Down	0.95
POX06748	Hypothetical protein	NA	38.14	108.97	1.51	Up	0.85
POX06752	Hypothetical protein	NA	29.14	79.54	1.45	Up	0.82
POX06753	Hypothetical protein	NA	2065.50	4537.18	1.14	Up	0.86
POX06754	Hypothetical protein	NA	140.23	16.85	-3.06	Down	0.91
POX06757	Hypothetical protein	NA	140.95	38.71	-1.86	Down	0.88
POX06758	Hypothetical protein	NA	234.78	67.03	-1.81	Down	0.90
POX06761	Hypothetical protein	NA	517.43	52.96	-3.29	Down	0.94
POX06766	Hypothetical protein	NA	109.68	231.20	1.08	Up	0.82
POX06767	Hypothetical protein	NA	132.42	338.69	1.35	Up	0.87
POX06768	Hypothetical protein	NA	263.99	29.57	-3.16	Down	0.93
POX06783	Putative endo-beta-1,4-xylanase	CBM1;GH11	1513.14	598.91	-1.34	Down	0.89
POX06788	Hypothetical protein	NA	410.52	1991.52	2.28	Up	0.94
POX06794	Hypothetical protein	NA	363.77	1846.10	2.34	Up	0.94
POX06800	Hypothetical protein	NA	246.64	106.12	-1.22	Down	0.85
POX06807	Hypothetical protein	NA	413.74	2385.15	2.53	Up	0.94
POX06818	Heat shock protein DnaJ	GT41	27.69	73.71	1.41	Up	0.81
POX06820	Hypothetical protein	NA	1310.47	6212.62	2.25	Up	0.94
POX06823	Hypothetical protein	NA	156.05	347.35	1.15	Up	0.84
POX06834	Hypothetical protein	NA	50.15	4.11	-3.61	Down	0.85
POX06837	Hypothetical protein	NA	152.21	43.48	-1.81	Down	0.88
POX06842	Hypothetical protein	NA	70.94	266.93	1.91	Up	0.91
POX06846	Hypothetical protein	NA	68.35	271.20	1.99	Up	0.91
POX06848	Hypothetical protein	NA	37.16	108.63	1.55	Up	0.85
POX06853	Hypothetical protein	NA	262.33	1182.20	2.17	Up	0.93
POX06862	Hypothetical protein	NA	13.74	324.93	4.56	Up	0.94
POX06875	Hypothetical protein	NA	482.88	2362.02	2.29	Up	0.94
POX06879	Hypothetical protein	NA	103.60	43.94	-1.24	Down	0.81

POX06884	Hypothetical protein	NA	762.25	3630.41	2.25	Up	0.94
POX06886	Hypothetical protein	NA	120.82	20.78	-2.54	Down	0.90
POX06887	Hypothetical protein	NA	203.21	87.37	-1.22	Down	0.84
POX06891	Hypothetical protein	NA	56.83	18.16	-1.65	Down	0.80
POX06892	Hypothetical protein	NA	261.86	1228.82	2.23	Up	0.93
POX06897	Hypothetical protein	NA	43.99	154.65	1.81	Up	0.88
POX06900	Putative beta-glucuronidase	GH2	362.98	14.66	-4.63	Down	0.94
POX06901	Hypothetical protein	NA	11.07	262.72	4.57	Up	0.94
POX06904	Hypothetical protein	NA	322.45	770.43	1.26	Up	0.87
POX06907	Hypothetical protein	NA	88.08	19.20	-2.20	Down	0.87
POX06916	Hypothetical protein	NA	199.70	81.34	-1.30	Down	0.85
POX06927	Hypothetical protein	NA	34.98	120.57	1.79	Up	0.87
POX06936	Hypothetical protein	NA	41.80	97.67	1.22	Up	0.80
POX06940	Hypothetical protein	NA	107.25	461.29	2.10	Up	0.92
POX06946	Hypothetical protein	NA	58.60	195.60	1.74	Up	0.89
POX06951	Hypothetical protein	NA	55.35	331.48	2.58	Up	0.93
POX06957	Hypothetical protein	NA	34.22	87.76	1.36	Up	0.81
POX06959	Putative endo-beta-1,3-glucanase	GH81	118.16	20.80	-2.51	Down	0.90
POX06961	Hypothetical protein	NA	451.03	2149.34	2.25	Up	0.94
POX06963	Hypothetical protein	NA	70.18	158.69	1.18	Up	0.83
POX06968	Hypothetical protein	NA	19.12	65.47	1.78	Up	0.83
POX06972	Hypothetical protein	NA	70.83	163.81	1.21	Up	0.83
POX06981	Hypothetical protein	NA	115.51	1064.83	3.20	Up	0.95
POX06987	Hypothetical protein	NA	114.48	23.52	-2.28	Down	0.89
POX06993	Hypothetical protein	NA	10.42	44.51	2.09	Up	0.80
POX06996	Hypothetical protein	NA	233.43	24.96	-3.23	Down	0.93
POX06998	Putative d-4,5 unsaturated beta-glucuronyl hydrolase	GH88	110.63	16.07	-2.78	Down	0.90
POX06999	Hypothetical protein	NA	82.50	13.44	-2.62	Down	0.88
POX07002	Hypothetical protein	NA	271.23	1111.74	2.04	Up	0.93
POX07010	Hypothetical protein	NA	91.46	207.94	1.18	Up	0.84
POX07011	Hypothetical protein	NA	358.10	1898.49	2.41	Up	0.94
POX07016	Hypothetical protein	NA	108.36	220.80	1.03	Up	0.81
POX07031	Hypothetical protein	NA	107.29	246.35	1.20	Up	0.84
POX07033	Hypothetical protein	NA	4.28	51.44	3.59	Up	0.85
POX07053	Hypothetical protein	NA	64.52	141.04	1.13	Up	0.81
POX07063	Hypothetical protein	NA	292.91	896.56	1.61	Up	0.91

POX07068	Hypothetical protein	NA	123.06	334.88	1.44	Up	0.88
POX07070	Hypothetical protein	NA	59.79	128.76	1.11	Up	0.80
POX07072	Hypothetical protein	NA	85.13	31.00	-1.46	Down	0.82
POX07081	Hypothetical protein	NA	455.68	90.98	-2.32	Down	0.93
POX07083	Putative beta-1,3-1,4-glucanase	GH16	60.90	10.58	-2.52	Down	0.85
POX07086	Hypothetical protein	NA	74.76	213.99	1.52	Up	0.88
POX07087	Hypothetical protein	NA	462.36	1072.97	1.21	Up	0.87
POX07097	Hypothetical protein	NA	293.66	1074.90	1.87	Up	0.92
POX07104	Putative alpha-xylosidase	GH31	362.97	76.90	-2.24	Down	0.92
POX07107	Hypothetical protein	NA	106.22	485.90	2.19	Up	0.93
POX07108	Hypothetical protein	NA	81.47	217.22	1.41	Up	0.87
POX07109	Hypothetical protein	NA	295.50	21.12	-3.81	Down	0.94
POX07116	Hypothetical protein	NA	94.89	39.50	-1.26	Down	0.81
POX07118	Hypothetical protein	NA	81.95	179.00	1.13	Up	0.82
POX07120	Hypothetical protein	NA	31.81	138.34	2.12	Up	0.89
POX07127	Hypothetical protein	NA	55.22	124.45	1.17	Up	0.81
POX07129	Hypothetical protein	NA	103.21	45.06	-1.20	Down	0.80
POX07132	Hypothetical protein	NA	76.33	221.20	1.54	Up	0.88
POX07145	Putative chitinase	GH18	71.39	179.38	1.33	Up	0.85
POX07153	Hypothetical protein	NA	108.18	228.59	1.08	Up	0.82
POX07163	Hypothetical protein	NA	20.20	74.03	1.87	Up	0.84
POX07166	Hypothetical protein	NA	19.30	59.79	1.63	Up	0.81
POX07175	Hypothetical protein	NA	52.52	149.85	1.51	Up	0.86
POX07201	Putative exo-beta-1,3-glucanase	GH17	160.32	1441.24	3.17	Up	0.95
POX07202	Hypothetical protein	NA	46.64	254.70	2.45	Up	0.92
POX07232	Hypothetical protein	NA	50.28	10.76	-2.22	Down	0.82
POX07234	Hypothetical protein	NA	195.95	431.12	1.14	Up	0.85
POX07236	Hypothetical protein	NA	31.18	79.15	1.34	Up	0.80
POX07237	Hypothetical protein	NA	28.71	0.41	-6.14	Down	0.80
POX07238	Hypothetical protein	NA	266.32	54.86	-2.28	Down	0.92
POX07249	Hypothetical protein	NA	14.78	62.92	2.09	Up	0.84
POX07258	Hypothetical protein	NA	49.15	3.22	-3.93	Down	0.85
POX07265	Hypothetical protein	NA	292.78	1140.19	1.96	Up	0.93
POX07266	Hypothetical protein	NA	120.40	44.39	-1.44	Down	0.85
POX07270	Hypothetical protein	NA	100.27	9.31	-3.43	Down	0.90
POX07285	Putative beta-1,3-mannanase	GH5	81.16	2.93	-4.79	Down	0.90
POX07300	Hypothetical protein	NA	39.52	184.68	2.22	Up	0.91
POX07325	Hypothetical protein	NA	4.08	35.80	3.13	Up	0.80

POX07327	Hypothetical protein	CE10	81.95	29.35	-1.48	Down	0.82
POX07328	Hypothetical protein	NA	140.76	12.03	-3.55	Down	0.92
POX07330	Hypothetical protein	NA	48.94	146.27	1.58	Up	0.87
POX07339	Hypothetical protein	NA	4897.65	296.99	-4.04	Down	0.95
POX07346	Hypothetical protein	NA	112.28	14.06	-3.00	Down	0.90
POX07360	Hypothetical protein	NA	4.66	42.80	3.20	Up	0.83
POX07363	Hypothetical protein	NA	17.17	2879.49	7.39	Up	0.98
POX07374	Hypothetical protein	NA	94.00	707.59	2.91	Up	0.94
POX07375	Putative beta-1,3-glucanase	GH16	37.69	677.71	4.17	Up	0.95
POX07376	Chitin binding domain-containing protein	AA5	20.56	3082.71	7.23	Up	0.98
POX07383	Hypothetical protein	NA	30.30	434.77	3.84	Up	0.94
POX07393	Hypothetical protein	NA	277.82	25.27	-3.46	Down	0.93
POX07397	Carbohydrate binding domain-containing protein	NA	2.75	158.14	5.85	Up	0.93
POX07399	Putative UDP-Glc: sterol glucosyltransferase	GT1	5.53	50.77	3.20	Up	0.85
POX07405	Hypothetical protein	NA	104.99	36.71	-1.52	Down	0.85
POX07409	Hypothetical protein	GH131	62.68	9.69	-2.69	Down	0.86
POX07410	Hypothetical protein	GT41	73.52	9.47	-2.96	Down	0.88
POX07419	Hypothetical protein	NA	194.88	97.25	-1.00	Down	0.80
POX07422	Hypothetical protein	NA	74.08	20.72	-1.84	Down	0.84
POX07423	Chitin binding domain- and peptidoglycan binding domain-containing protein	CBM18	35.25	162.90	2.21	Up	0.90
POX07424	Putative chitinase	GH18	25.64	173.22	2.76	Up	0.92
POX07428	Hypothetical protein	NA	595.85	59.58	-3.32	Down	0.94
POX07432	Hypothetical protein	NA	611.34	300.49	-1.02	Down	0.83
POX07441	Putative alpha-L-arabinofuranosidase	GH51	72.80	27.08	-1.43	Down	0.81
POX07450	Hypothetical protein	NA	53.70	153.08	1.51	Up	0.86
POX07457	Hypothetical protein	NA	161.38	1677.42	3.38	Up	0.95
POX07466	Hypothetical protein	NA	24.79	71.97	1.54	Up	0.82
POX07470	Putative beta-1,6-N-acetylglucosaminidase	GH20	124.39	44.27	-1.49	Down	0.85
POX07471	Hypothetical protein	NA	30.82	107.17	1.80	Up	0.86
POX07491	Hypothetical protein	NA	147.55	24.45	-2.59	Down	0.91
POX07497	Hypothetical protein	NA	328.52	28.72	-3.52	Down	0.94

POX07502	Hypothetical protein	NA	729.02	1824.38	1.32	Up	0.89
POX07503	Hypothetical protein	NA	134.40	599.32	2.16	Up	0.93
POX07507	Hypothetical protein	NA	248.75	92.77	-1.42	Down	0.87
POX07513	Hypothetical protein	NA	788.33	2166.41	1.46	Up	0.90
POX07524	Putative alpha-L-rhamnosidase	GH78;CBM67	65.98	18.93	-1.80	Down	0.83
POX07532	Hypothetical protein	NA	20.10	918.60	5.51	Up	0.95
POX07534	Putative beta-1,3-glucanosyltransferase	GH17	992.38	2062.06	1.06	Up	0.84
POX07535	Putative endo-beta-1,4-glucanase	GH12	1482.74	516.92	-1.52	Down	0.90
POX07573	Putative beta-N-acetylhexosaminidase	GH3	49.55	118.15	1.25	Up	0.82
POX07576	Hypothetical protein	NA	1.25	108.69	6.44	Up	0.92
POX07580	Hypothetical protein	NA	89.94	23.90	-1.91	Down	0.86
POX07582	Hypothetical protein	NA	3678.11	1209.97	-1.60	Down	0.91
POX07585	Hypothetical protein	NA	47.70	125.83	1.40	Up	0.84
POX07586	Hypothetical protein	NA	95.72	39.96	-1.26	Down	0.81
POX07594	Hypothetical protein	NA	19.04	91.33	2.26	Up	0.88
POX07601	Hypothetical protein	NA	56.84	156.88	1.46	Up	0.86
POX07608	Hypothetical protein	NA	154.54	334.89	1.12	Up	0.84
POX07615	Hypothetical protein	NA	36.19	101.07	1.48	Up	0.84
POX07622	Hypothetical protein	NA	12.04	66.92	2.47	Up	0.86
POX07624	Hypothetical protein	NA	225.81	87.41	-1.37	Down	0.86
POX07625	Hypothetical protein	NA	37.72	111.52	1.56	Up	0.85
POX07626	Hypothetical protein	NA	12.24	79.42	2.70	Up	0.88
POX07644	Hypothetical protein	NA	1367.21	145.14	-3.24	Down	0.95
POX07646	Hypothetical protein	NA	62.18	249.36	2.00	Up	0.91
POX07668	Hypothetical protein	NA	289.82	22.09	-3.71	Down	0.93
POX07669	Hypothetical protein	NA	354.05	32.86	-3.43	Down	0.94
POX07688	Hypothetical protein	NA	290.67	57.84	-2.33	Down	0.92
POX07701	Hypothetical protein	NA	61.85	197.78	1.68	Up	0.88
POX07702	Hypothetical protein	NA	25.18	94.51	1.91	Up	0.86
POX07713	Hypothetical protein	NA	30.53	119.75	1.97	Up	0.88
POX07716	Hypothetical protein	NA	111.44	29.22	-1.93	Down	0.87
POX07730	Hypothetical protein	NA	40.98	118.54	1.53	Up	0.85
POX07736	Hypothetical protein	NA	159.18	48.81	-1.71	Down	0.88
POX07739	Hypothetical protein	NA	137.82	16.81	-3.04	Down	0.91
POX07747	Hypothetical protein	NA	158.51	32.16	-2.30	Down	0.90
POX07757	Hypothetical protein	NA	0.16	27.53	7.43	Up	0.81
POX07761	Hypothetical protein	NA	38.21	103.91	1.44	Up	0.84

POX07771	Hypothetical protein	NA	68.76	144.37	1.07	Up	0.80
POX07772	Hypothetical protein	NA	1.64	53.82	5.03	Up	0.87
POX07773	Hypothetical protein	NA	22.83	88.34	1.95	Up	0.86
POX07782	Hypothetical protein	NA	24.77	233.27	3.24	Up	0.93
POX07788	Hypothetical protein	NA	27.17	95.91	1.82	Up	0.86
POX07802	Putative beta-fructofuranosidase	GH32	13.30	77.43	2.54	Up	0.87
POX07804	Hypothetical protein	NA	0.20	135.21	9.38	Up	0.96
POX07805	Hypothetical protein	NA	125.17	54.75	-1.19	Down	0.82
POX07813	Hypothetical protein	NA	506.72	1540.07	1.60	Up	0.91
POX07820	Ecm33 domain-containing protein	NA	620.91	1642.76	1.40	Up	0.89
POX07822	Hypothetical protein	NA	249.66	742.09	1.57	Up	0.90
POX07832	Expansin-like	NA	258.24	624.64	1.27	Up	0.87
POX07863	Hypothetical protein	NA	50.67	2.67	-4.24	Down	0.86
POX07883	Hypothetical protein	NA	53.09	13.91	-1.93	Down	0.81
POX07890	Putative pectate lyase	PL1	374.31	5.66	-6.05	Down	0.95
POX07891	Putative alpha-L-arabinofuranosidase	CBM35;CBM36;<br>CBM6;GH43	1033.68	78.25	-3.72	Down	0.95
POX07896	Hypothetical protein	NA	218.78	682.08	1.64	Up	0.90
POX07899	Hypothetical protein	NA	18.32	66.54	1.86	Up	0.83
POX07900	Putative endo-beta-1,6-galactanase	GH30	395.66	94.02	-2.07	Down	0.92
POX07913	Hypothetical protein	NA	24.73	256.42	3.37	Up	0.93
POX07933	Hypothetical protein	NA	17.46	69.60	2.00	Up	0.84
POX07938	Hypothetical protein	NA	6.59	142.66	4.44	Up	0.92
POX07940	Hypothetical protein	NA	461.53	158.59	-1.54	Down	0.90
POX07946	Hypothetical protein	NA	20.10	67.19	1.74	Up	0.83
POX07954	Hypothetical protein	NA	134.99	4.99	-4.76	Down	0.92
POX07960	Hypothetical protein	NA	113.57	43.50	-1.38	Down	0.83
POX07974	Hypothetical protein	NA	55.10	123.25	1.16	Up	0.81
POX07980	Hypothetical protein	NA	422.92	1727.02	2.03	Up	0.93
POX07989	Hypothetical protein	NA	181.26	416.41	1.20	Up	0.86
POX08027	Hypothetical protein	NA	30.74	0.44	-6.13	Down	0.81
POX08029	Hypothetical protein	NA	118.19	20.09	-2.56	Down	0.90
POX08031	Hypothetical protein	NA	24.64	98.55	2.00	Up	0.87
POX08042	Hypothetical protein	NA	72.07	176.07	1.29	Up	0.85
POX08044	Hypothetical protein	NA	119.00	332.28	1.48	Up	0.88
POX08046	Hypothetical protein	NA	16.85	81.21	2.27	Up	0.87
POX08048	Hypothetical protein	NA	17.97	66.14	1.88	Up	0.83
POX08049	Hypothetical protein	NA	0.01	49.70	12.28	Up	0.90
POX08052	Hypothetical protein	NA	244.67	55.23	-2.15	Down	0.91

POX08055	Hypothetical protein	NA	17.97	60.17	1.74	Up	0.82
POX08066	Hypothetical protein	NA	266.10	1229.47	2.21	Up	0.93
POX08067	Putative pectin methylesterase	CE8	21.62	63.47	1.55	Up	0.81
POX08079	Hypothetical protein	NA	42.22	189.18	2.16	Up	0.91
POX08085	Hypothetical protein	NA	356.05	1779.27	2.32	Up	0.94
POX08089	Hypothetical protein	NA	84.03	685.42	3.03	Up	0.94
POX08090	Hypothetical protein	NA	104.17	350.64	1.75	Up	0.90
POX08097	Hypothetical protein	NA	10.37	143.50	3.79	Up	0.92
POX08103	Hypothetical protein	NA	314.52	37.81	-3.06	Down	0.93
POX08104	Hypothetical protein	NA	210.04	98.81	-1.09	Down	0.82
POX08116	Hypothetical protein	NA	64.11	20.45	-1.65	Down	0.81
POX08126	Hypothetical protein	NA	26.45	89.04	1.75	Up	0.85
POX08127	Hypothetical protein	NA	7.71	58.81	2.93	Up	0.86
POX08136	Hypothetical protein	NA	61.68	487.33	2.98	Up	0.94
POX08140	Putative alpha-L-arabinofuranosidase	GH43	13.39	86.22	2.69	Up	0.88
POX08145	Hypothetical protein	NA	59.17	205.71	1.80	Up	0.89
POX08158	Hypothetical protein	NA	2842.29	376.83	-2.92	Down	0.95
POX08172	Hypothetical protein	NA	302.32	109.68	-1.46	Down	0.88
POX08175	Hypothetical protein	AA7	5.98	39.40	2.72	Up	0.81
POX08178	Hypothetical protein	NA	64.68	139.65	1.11	Up	0.81
POX08185	Hypothetical protein	NA	98.92	39.30	-1.33	Down	0.82
POX08190	Hypothetical protein	NA	664.09	67.86	-3.29	Down	0.94
POX08194	Hypothetical protein	NA	332.69	131.20	-1.34	Down	0.87
POX08198	Hypothetical protein	NA	353.40	2066.16	2.55	Up	0.94
POX08199	Hypothetical protein	NA	314.09	1981.52	2.66	Up	0.94
POX08200	Hypothetical protein	NA	210.48	1090.05	2.37	Up	0.94
POX08202	Hypothetical protein	NA	82.34	200.65	1.28	Up	0.85
POX08210	Hypothetical protein	NA	308.60	1465.24	2.25	Up	0.94
POX08217	Hypothetical protein	NA	139.85	614.35	2.14	Up	0.93
POX08219	Hypothetical protein	NA	169.69	63.99	-1.41	Down	0.86
POX08223	Hypothetical protein	NA	35.57	135.24	1.93	Up	0.88
POX08225	Hypothetical protein	NA	356.89	1525.06	2.10	Up	0.93
POX08235	Hypothetical protein	NA	286.66	69.78	-2.04	Down	0.91
POX08236	Hypothetical protein	NA	145.27	33.87	-2.10	Down	0.89
POX08243	Hypothetical protein	NA	109.53	342.06	1.64	Up	0.90
POX08249	Hypothetical protein	NA	47.90	110.53	1.21	Up	0.81
POX08255	Hypothetical protein	NA	279.27	100.08	-1.48	Down	0.88
POX08265	Hypothetical protein	NA	237.29	599.89	1.34	Up	0.88

POX08273	Hypothetical protein	NA	2.78	55.39	4.32	Up		0.86
POX08274	Hypothetical protein	NA	79.53	1.32	-5.91	Down		0.90
POX08291	Hypothetical protein	NA	94.16	211.75	1.17	Up		0.84
POX08292	Hypothetical protein	NA	101.48	417.92	2.04	Up		0.92
POX08305	Hypothetical protein	NA	69.62	4.57	-3.93	Down		0.88
POX08307	Hypothetical protein	NA	588.37	3151.33	2.42	Up		0.94
POX08314	Hypothetical protein	NA	316.20	1306.06	2.05	Up		0.93
POX08316	Hypothetical protein	NA	225.81	20.45	-3.46	Down		0.93
POX08322	Hypothetical protein	NA	389.62	942.50	1.27	Up		0.87
POX08323	Hypothetical protein	NA	38.70	120.29	1.64	Up		0.86
POX08334	Hypothetical protein	NA	37.21	93.47	1.33	Up		0.82
POX08339	Hypothetical protein	NA	63.32	4.50	-3.82	Down		0.87
POX08340	Hypothetical protein	NA	79.49	20.54	-1.95	Down		0.85
POX08341	Hypothetical protein	NA	165.24	43.02	-1.94	Down		0.89
POX08343	Hypothetical protein	NA	15.36	71.32	2.22	Up		0.86
POX08347	Putative alpha-mannosidase	GH38	163.78	68.91	-1.25	Down		0.84
POX08353	Hypothetical protein	NA	139.01	58.59	-1.25	Down		0.83
POX08356	Hypothetical protein	NA	74.63	5.49	-3.76	Down		0.88
POX08359	Hypothetical protein	NA	343.74	909.73	1.40	Up		0.89
POX08371	Hypothetical protein	NA	16.68	507.33	4.93	Up		0.95
POX08382	Hypothetical protein	NA	35.20	1.48	-4.57	Down		0.82
POX08383	Hypothetical protein	NA	55.56	12.93	-2.10	Down		0.83
POX08386	Hypothetical protein	NA	35.48	2.45	-3.86	Down		0.81
POX08389	Hypothetical protein	NA	20.98	63.21	1.59	Up		0.81
POX08390	Putative beta-1,6-glucanase	GH30	109.38	266.36	1.28	Up		0.86
POX08418	Hypothetical protein	CE10	161.47	58.45	-1.47	Down		0.86
POX08426	Hypothetical protein	NA	55.25	1.36	-5.34	Down		0.87
POX08427	Hypothetical protein	NA	104.33	46.28	-1.17	Down		0.80
POX08429	Hypothetical protein	NA	117.67	47.31	-1.31	Down		0.83
POX08438	Hypothetical protein	NA	71.61	12.98	-2.46	Down		0.86
POX08439	Hypothetical protein	NA	156.84	45.75	-1.78	Down		0.88
POX08440	Hypothetical protein	NA	120.54	19.70	-2.61	Down		0.90
POX08441	Hypothetical protein	NA	218.21	33.36	-2.71	Down		0.92
POX08443	Hypothetical protein	NA	283.95	32.65	-3.12	Down		0.93
POX08450	Hypothetical protein	NA	233.09	35.74	-2.71	Down		0.92
POX08451	Hypothetical protein	NA	105.18	10.25	-3.36	Down		0.90
POX08452	Hypothetical protein	NA	140.09	6.29	-4.48	Down		0.92
POX08456	Hypothetical protein	NA	865.43	49.16	-4.14	Down		0.95

POX08457	Hypothetical protein	NA	265.73	4.76	-5.80	Down	0.94
POX08459	Hypothetical protein	NA	34.29	164.89	2.27	Up	0.90
POX08473	Hypothetical protein	NA	176.65	45.08	-1.97	Down	0.90
POX08474	Hypothetical protein	NA	90.90	301.03	1.73	Up	0.90
POX08476	Putative rhamnogalacturonan acetylesterase	CE12	284.08	38.07	-2.90	Down	0.93
POX08478	Hypothetical protein	NA	169.76	2992.12	4.14	Up	0.95
POX08483	Putative alpha-N-acetylglucosaminidase	GH89	90.88	13.93	-2.71	Down	0.89
POX08484	Endo-β-1,4-xylanase	CBM1;GH11	1712.77	460.35	-1.90	Down	0.92
POX08485	Swollenin	CBM1	1641.97	326.71	-2.33	Down	0.94
POX08486	Hypothetical protein	NA	1768.49	566.76	-1.64	Down	0.91
POX08497	Hypothetical protein	NA	83.87	341.43	2.03	Up	0.92
POX08502	Hypothetical protein	NA	619.82	2620.09	2.08	Up	0.93
POX08524	Hypothetical protein	NA	35.62	153.82	2.11	Up	0.90
POX08525	Hypothetical protein	NA	347.31	1454.66	2.07	Up	0.93
POX08531	Hypothetical protein	NA	24.92	69.53	1.48	Up	0.81
POX08532	Hypothetical protein	NA	78.93	183.91	1.22	Up	0.84
POX08561	Hypothetical protein	NA	148.47	337.16	1.18	Up	0.85
POX08562	Hypothetical protein	NA	350.19	2485.24	2.83	Up	0.95
POX08567	Hypothetical protein	NA	73.33	205.44	1.49	Up	0.87
POX08583	Hypothetical protein	NA	176.99	645.34	1.87	Up	0.92
POX08602	Hypothetical protein	NA	5.53	39.52	2.84	Up	0.81
POX08613	Hypothetical protein	NA	212.90	17.40	-3.61	Down	0.93
POX08617	Hypothetical protein	NA	212.57	13.22	-4.01	Down	0.93
POX08622	Hypothetical protein	NA	124.76	319.52	1.36	Up	0.87
POX08627	Hypothetical protein	NA	63.15	12.22	-2.37	Down	0.85
POX08635	Hypothetical protein	NA	34.24	100.89	1.56	Up	0.85
POX08644	Hypothetical protein	NA	173.68	371.13	1.10	Up	0.84
POX08646	Hypothetical protein	NA	169.05	30.30	-2.48	Down	0.91
POX08652	Hypothetical protein	NA	368.56	101.93	-1.85	Down	0.91
POX08659	Hypothetical protein	NA	6.33	78.60	3.64	Up	0.89
POX08660	Hypothetical protein	NA	118.24	25.29	-2.22	Down	0.89
POX08663	Hypothetical protein	NA	77.31	28.33	-1.45	Down	0.82
POX08665	Hypothetical protein	NA	136.86	51.72	-1.40	Down	0.85
POX08671	Hypothetical protein	NA	139.73	383.15	1.46	Up	0.89
POX08673	Hypothetical protein	NA	366.55	1132.01	1.63	Up	0.91
POX08676	Hypothetical protein	NA	236.97	2.97	-6.32	Down	0.95

POX08677	Hypothetical protein	NA	366.70	1614.99	2.14	Up	0.93
POX08696	Hypothetical protein	NA	137.25	38.59	-1.83	Down	0.88
POX08698	Hypothetical protein	NA	22.98	185.67	3.01	Up	0.92
POX08700	Hypothetical protein	NA	20.76	100.10	2.27	Up	0.88
POX08702	Hypothetical protein	NA	50.52	192.05	1.93	Up	0.90
POX08706	Hypothetical protein	NA	22.52	141.34	2.65	Up	0.91
POX08723	Hypothetical protein	NA	63.32	180.98	1.51	Up	0.87
POX08726	Hypothetical protein	NA	11.74	58.28	2.31	Up	0.84
POX08750	Hypothetical protein	NA	9.74	52.68	2.43	Up	0.83
POX08753	Hypothetical protein	NA	151.49	38.26	-1.99	Down	0.89
POX08766	Hypothetical protein	NA	81.40	13.23	-2.62	Down	0.88
POX08768	alpha-trehalase	GH65	12.61	68.25	2.44	Up	0.86
POX08776	Hypothetical protein	NA	125.73	55.30	-1.18	Down	0.81
POX08780	Hypothetical protein	NA	28.27	75.81	1.42	Up	0.81
POX08782	Hypothetical protein	NA	18.34	82.75	2.17	Up	0.87
POX08783	Hypothetical protein	NA	56.10	153.40	1.45	Up	0.86
POX08785	Putative rhamnogalacturonan lyase	PL4	388.89	13.77	-4.82	Down	0.94
POX08787	Hypothetical protein	NA	140.76	17.62	-3.00	Down	0.91
POX08788	Hypothetical protein	NA	85.24	30.65	-1.48	Down	0.83
POX08789	Hypothetical protein	NA	53.67	410.16	2.93	Up	0.94
POX08796	Hypothetical protein	NA	54.50	14.98	-1.86	Down	0.81
POX08797	Hypothetical protein	NA	175.71	52.69	-1.74	Down	0.89
POX08800	Hypothetical protein	NA	22.83	0.01	-11.16	Down	0.80
POX08801	Hypothetical protein	NA	82.25	366.46	2.16	Up	0.92
POX08804	Hypothetical protein	NA	72.44	273.45	1.92	Up	0.91
POX08812	Hypothetical protein	NA	100.95	28.51	-1.82	Down	0.86
POX08814	Hypothetical protein	NA	122.42	8.82	-3.79	Down	0.91
POX08818	Hypothetical protein	NA	133.86	18.66	-2.84	Down	0.91
POX08819	Hypothetical protein	NA	355.93	849.31	1.25	Up	0.87
POX08821	Hypothetical protein	NA	1015.19	273.89	-1.89	Down	0.92
POX08822	Hypothetical protein	NA	40.05	752.40	4.23	Up	0.95
POX08831	Hypothetical protein	NA	159.00	5099.90	5.00	Up	0.96
POX08835	Hypothetical protein	NA	431.79	1164.89	1.43	Up	0.90
POX08849	Hypothetical protein	NA	9.02	70.62	2.97	Up	0.87
POX08872	Hypothetical protein	NA	42.46	9.20	-2.21	Down	0.80
POX08873	Hypothetical protein	NA	30.32	0.68	-5.49	Down	0.80
POX08874	Hypothetical protein	NA	156.45	15.62	-3.32	Down	0.92

POX08875	Putative unsaturated rhamnogalacturonyl hydrolase	GH105	50.95	11.28	-2.18	Down	0.82
POX08877	Hypothetical protein	NA	136.94	22.89	-2.58	Down	0.90
POX08878	Hypothetical protein	CE10	192.17	19.70	-3.29	Down	0.92
POX08879	Hypothetical protein	NA	61.85	12.29	-2.33	Down	0.85
POX08897	Putative cellulose monooxygenase	CBM1;AA9	1096.42	142.73	-2.94	Down	0.94
POX08901	Hypothetical protein	NA	53.15	7.64	-2.80	Down	0.84
POX08903	Putative chitosanase	GH75	193.22	15.18	-3.67	Down	0.93
POX08906	Hypothetical protein	NA	10398.58	1618.29	-2.68	Down	0.95
POX08910	Hypothetical protein	NA	265.14	36.20	-2.87	Down	0.93
POX08939	Hypothetical protein	NA	72.83	283.69	1.96	Up	0.91
POX08943	Hypothetical protein	NA	23.27	180.42	2.96	Up	0.92
POX08945	Hypothetical protein	NA	1003.70	449.03	-1.16	Down	0.86
POX08947	Hypothetical protein	NA	15.24	92.44	2.60	Up	0.88
POX08949	Hypothetical protein	NA	116.70	1064.45	3.19	Up	0.95
POX08954	Carbohydrate binding domain-containing protein	NA	23.93	263.27	3.46	Up	0.93
POX08958	Hypothetical protein	NA	16.16	59.63	1.88	Up	0.82
POX08964	Hypothetical protein	NA	48.91	152.80	1.64	Up	0.87
POX08974	Hypothetical protein	NA	29.78	76.98	1.37	Up	0.80
POX08976	Hypothetical protein	NA	8.58	54.52	2.67	Up	0.84
POX08977	Hypothetical protein	NA	44.41	155.28	1.81	Up	0.88
POX08990	Putative endo-beta-1,4-xylanase	GH10	9284.97	83.68	-6.79	Down	0.97
POX08991	Hypothetical protein	NA	229.60	10.54	-4.45	Down	0.93
POX08998	Hypothetical protein	NA	13.88	78.39	2.50	Up	0.87
POX09006	Hypothetical protein	NA	90.35	35.84	-1.33	Down	0.81
POX09010	Hypothetical protein	NA	43.28	117.53	1.44	Up	0.85
POX09012	Hypothetical protein	NA	88.16	302.39	1.78	Up	0.90
POX09017	Hypothetical protein	NA	48.09	228.09	2.25	Up	0.91
POX09025	Hypothetical protein	NA	626.60	236.76	-1.40	Down	0.88
POX09026	Hypothetical protein	NA	108.18	441.52	2.03	Up	0.92
POX09028	Hypothetical protein	NA	154.69	575.00	1.89	Up	0.92
POX09030	Hypothetical protein	NA	16.88	66.71	1.98	Up	0.84
POX09032	Hypothetical protein	NA	47.79	10.76	-2.15	Down	0.81
POX09040	Hypothetical protein	NA	52.39	709.94	3.76	Up	0.95
POX09042	Hypothetical protein	NA	56.00	173.35	1.63	Up	0.88
POX09049	Hypothetical protein	NA	139.83	301.44	1.11	Up	0.83

POX09064	Hypothetical protein	NA	156.72	41.81	-1.91	Down	0.89
POX09065	Hypothetical protein	NA	85.88	313.48	1.87	Up	0.91
POX09066	Hypothetical protein	NA	252.30	34.58	-2.87	Down	0.93
POX09076	Hypothetical protein	NA	12.97	1219.21	6.55	Up	0.97
POX09084	Hypothetical protein	NA	32.56	392.84	3.59	Up	0.94
POX09085	Putative alpha-galactosidase	CBM1;GH27;GH36	947.61	365.24	-1.38	Down	0.89
POX09088	Hypothetical protein	NA	11.18	45.94	2.04	Up	0.80
POX09092	Hypothetical protein	NA	58.86	6.19	-3.25	Down	0.86
POX09101	Hypothetical protein	NA	1186.33	216.40	-2.45	Down	0.94
POX09103	Hypothetical protein	NA	106.66	0.95	-6.81	Down	0.93
POX09104	Hypothetical protein	NA	33.54	1.58	-4.41	Down	0.81
POX09106	Hypothetical protein	NA	79.02	1.21	-6.03	Down	0.90
POX09108	Hypothetical protein	NA	114.15	12.81	-3.16	Down	0.90
POX09109	Hypothetical protein	NA	81.23	14.75	-2.46	Down	0.87
POX09110	Putative alpha-1,3-glucanase	CBM24;GH71	342.82	24.56	-3.80	Down	0.94
POX09112	Hypothetical protein	NA	205.07	56.13	-1.87	Down	0.90
POX09115	Hypothetical protein	NA	96.86	198.76	1.04	Up	0.81
POX09116	Hypothetical protein	NA	61.78	1.14	-5.76	Down	0.88
POX09117	Hypothetical protein	NA	313.60	4.28	-6.19	Down	0.95
POX09124	Hypothetical protein	NA	291.66	19.60	-3.90	Down	0.94
POX09135	Hypothetical protein	NA	49.88	8.10	-2.62	Down	0.83
POX09136	Hypothetical protein	NA	138.65	18.73	-2.89	Down	0.91
POX09137	Putative feruloyl esterase	CBM1;CE1	6046.06	282.38	-4.42	Down	0.95
POX09147	Hypothetical protein	NA	37.22	139.75	1.91	Up	0.88
POX09150	Hypothetical protein	NA	25.40	77.44	1.61	Up	0.83
POX09154	Putative alpha-L-arabinofuranosidase	GH43	48.15	2.78	-4.12	Down	0.85
POX09161	Hypothetical protein	NA	139.31	303.01	1.12	Up	0.84
POX09166	Hypothetical protein	NA	59.36	209.68	1.82	Up	0.89
POX09172	Hypothetical protein	NA	36.76	94.75	1.37	Up	0.82
POX09192	Hypothetical protein	NA	394.06	1440.94	1.87	Up	0.92
POX09195	Hypothetical protein	NA	93.82	1125.40	3.58	Up	0.95
POX09198	Hypothetical protein	NA	32.88	85.77	1.38	Up	0.81
POX09200	Hypothetical protein	NA	37.93	102.21	1.43	Up	0.84
POX09201	Hypothetical protein	NA	47.89	124.94	1.38	Up	0.84
POX09205	Hypothetical protein	NA	50.18	162.20	1.69	Up	0.88
POX09210	Hypothetical protein	NA	427.86	1021.22	1.26	Up	0.87
POX09233	Hypothetical protein	AA3;AA8	531.50	33.68	-3.98	Down	0.94

POX09243	Ribosomal protein S23	NA	726.70	3260.52	2.17	Up	0.94
POX09246	Hypothetical protein	NA	44.13	125.51	1.51	Up	0.85
POX09254	Hypothetical protein	NA	46.94	112.08	1.26	Up	0.82
POX09258	Hypothetical protein	GH127	884.34	66.66	-3.73	Down	0.95
POX09259	Hypothetical protein	NA	42.15	104.61	1.31	Up	0.82
POX09280	Ribosomal protein L6	NA	205.26	689.17	1.75	Up	0.91
POX09284	Hypothetical protein	NA	5.19	38.88	2.90	Up	0.81
POX09287	Hypothetical protein	NA	5009.19	1253.61	-2.00	Down	0.93
POX09288	Hypothetical protein	NA	109.16	17.58	-2.63	Down	0.89
POX09289	Hypothetical protein	NA	245.34	8131.35	5.05	Up	0.96
POX09299	Hypothetical protein	NA	45.91	875.81	4.25	Up	0.95
POX09319	Hypothetical protein	NA	5.97	38.47	2.69	Up	0.80
POX09323	Hypothetical protein	NA	122.67	19.00	-2.69	Down	0.90
POX09324	Hypothetical protein	NA	53.03	5.78	-3.20	Down	0.85
POX09330	Hypothetical protein	NA	57.59	173.97	1.60	Up	0.88
POX09331	Hypothetical protein	NA	281.35	876.11	1.64	Up	0.91
POX09332	Hypothetical protein	NA	51.37	264.91	2.37	Up	0.92
POX09333	Hypothetical protein	NA	5.20	156.84	4.91	Up	0.93
POX09334	Hypothetical protein	NA	12.98	227.35	4.13	Up	0.93
POX09335	Hypothetical protein	NA	21.88	221.71	3.34	Up	0.93
POX09336	Hypothetical protein	NA	27.52	625.10	4.51	Up	0.95
POX09337	Hypothetical protein	NA	65.45	977.38	3.90	Up	0.95
POX09338	Hypothetical protein	NA	16.69	176.19	3.40	Up	0.92
POX09339	Hypothetical protein	NA	13.05	160.33	3.62	Up	0.92
POX09340	Hypothetical protein	NA	5.04	64.87	3.69	Up	0.87
POX09345	Hypothetical protein	NA	15.99	204.22	3.67	Up	0.93
POX09352	alpha-amylase Amy13A	CBM20;GH13	24.53	320.01	3.71	Up	0.94
POX09353	Hypothetical protein	CE10	577.87	79.73	-2.86	Down	0.94
POX09354	Hypothetical protein	NA	71.39	165.03	1.21	Up	0.83
POX09356	Hypothetical protein	NA	5.66	85.25	3.91	Up	0.89
POX09357	Hypothetical protein	NA	7.10	163.11	4.52	Up	0.93
POX09358	Hypothetical protein	NA	60.76	8075.01	7.05	Up	0.97
POX09359	Hypothetical protein	NA	9.16	74.33	3.02	Up	0.88
POX09360	Hypothetical protein	NA	15.57	92.56	2.57	Up	0.88
POX09361	Hypothetical protein	NA	40.35	845.71	4.39	Up	0.95
POX09364	Hypothetical protein	NA	0.88	87.42	6.63	Up	0.91
POX09365	Hypothetical protein	NA	56.93	1856.10	5.03	Up	0.96
POX09377	Hypothetical protein	NA	104.00	242.53	1.22	Up	0.85

POX09387	Putative chitinase	GH18	230.54	72.01	-1.68	Down	0.89
POX09390	Hypothetical protein	NA	426.67	162.18	-1.40	Down	0.88
POX09398	Hypothetical protein	NA	29.89	110.96	1.89	Up	0.87
POX09406	Hypothetical protein	NA	0.01	26.56	11.37	Up	0.83
POX09413	Hypothetical protein	NA	76.80	14.86	-2.37	Down	0.87
POX09428	Hypothetical protein	NA	668.35	1837.49	1.46	Up	0.90
POX09429	Hypothetical protein	NA	32.68	212.18	2.70	Up	0.92
POX09430	Hypothetical protein	NA	84.23	185.92	1.14	Up	0.82
POX09440	Hypothetical protein	NA	110.20	440.36	2.00	Up	0.92
POX09456	Hypothetical protein	NA	177.76	73.76	-1.27	Down	0.84
POX09469	Hypothetical protein	NA	354.68	7.79	-5.51	Down	0.95
POX09470	Hypothetical protein	NA	164.29	14.30	-3.52	Down	0.92
POX09472	Hypothetical protein	NA	208.82	19.77	-3.40	Down	0.93
POX09473	Hypothetical protein	NA	253.90	27.01	-3.23	Down	0.93
POX09498	Hypothetical protein	NA	117.60	36.16	-1.70	Down	0.86
POX09500	Hypothetical protein	NA	60.05	14.72	-2.03	Down	0.83
POX09508	Hypothetical protein	NA	127.63	43.64	-1.55	Down	0.86
POX09511	Hypothetical protein	NA	50.87	176.73	1.80	Up	0.89
POX09521	Hypothetical protein	NA	87.09	266.80	1.62	Up	0.89
POX09523	Hypothetical protein	NA	18.87	154.38	3.03	Up	0.92
POX09525	Hypothetical protein	NA	30.64	110.79	1.85	Up	0.87
POX09537	Hypothetical protein	NA	224.25	857.63	1.94	Up	0.92
POX09540	Hypothetical protein	NA	12.72	49.15	1.95	Up	0.81
POX09552	Hypothetical protein	NA	117.56	46.06	-1.35	Down	0.83
POX09559	Hypothetical protein	NA	156.08	62.73	-1.32	Down	0.84
POX09578	Hypothetical protein	CE10;CE1	4.30	276.10	6.00	Up	0.95
POX09591	Hypothetical protein	NA	7.84	80.50	3.36	Up	0.89
POX09616	Hypothetical protein	NA	169.11	6.34	-4.74	Down	0.93
POX09630	Hypothetical protein	NA	212.78	96.76	-1.14	Down	0.83
POX09632	Hypothetical protein	NA	46.13	11.02	-2.07	Down	0.81
POX09637	Hypothetical protein	NA	384.49	1824.42	2.25	Up	0.94
POX09654	Hypothetical protein	NA	41.82	148.20	1.83	Up	0.88
POX09658	Hypothetical protein	NA	52.37	131.50	1.33	Up	0.84
POX09665	Hypothetical protein	NA	163.32	399.93	1.29	Up	0.87
POX09666	Hypothetical protein	NA	1172.89	2910.75	1.31	Up	0.89
POX09667	Hypothetical protein	NA	1406.23	3228.75	1.20	Up	0.87
POX09668	Hypothetical protein	NA	142.36	56.58	-1.33	Down	0.84
POX09669	Hypothetical protein	NA	48.63	6.79	-2.84	Down	0.84

POX09676	Hypothetical protein	NA	4.85	394.43	6.34	Up	0.95
POX09677	Hypothetical protein	NA	18.60	614.67	5.05	Up	0.95
POX09687	Hypothetical protein	NA	485.23	17.53	-4.79	Down	0.95
POX09705	Hypothetical protein	NA	49.22	6.54	-2.91	Down	0.84
POX09717	Hypothetical protein	NA	37.41	245.48	2.71	Up	0.92
POX09718	Hypothetical protein	NA	1.73	83.89	5.60	Up	0.90
POX09719	Hypothetical protein	NA	1.31	82.80	5.98	Up	0.90
POX09732	Hypothetical protein	NA	165.16	9.13	-4.18	Down	0.92
POX09736	Hypothetical protein	NA	210.93	48.82	-2.11	Down	0.91
POX09739	Hypothetical protein	NA	63.93	10.97	-2.54	Down	0.86
POX09755	Hypothetical protein	NA	79.94	29.19	-1.45	Down	0.82
POX09759	Hypothetical protein	NA	719.75	132.98	-2.44	Down	0.94
POX09760	Hypothetical protein	NA	250.40	42.62	-2.55	Down	0.92
POX09766	Hypothetical protein	NA	152.86	397.19	1.38	Up	0.88
POX09772	Hypothetical protein	NA	93.85	21.30	-2.14	Down	0.87
POX09781	Hypothetical protein	NA	386.79	30.36	-3.67	Down	0.94
POX09783	Hypothetical protein	NA	179.76	57.92	-1.63	Down	0.88
POX09786	Hypothetical protein	NA	37.33	145.90	1.97	Up	0.89
POX09801	Putative exo-beta-1, 3-galactanase	GH43	71.95	16.46	-2.13	Down	0.85
POX09819	Hypothetical protein	NA	12.09	159.71	3.72	Up	0.92
POX09821	Hypothetical protein	NA	70.51	1772.37	4.65	Up	0.95
POX09822	Hypothetical protein	NA	45.08	1224.02	4.76	Up	0.95

Note: CAZy, Carbohydrate-Active Enzymes; GH, Glycoside hydrolase; CE, Carbohydrate esterase; AA, Auxiliary activities; CWDEs, Plant cell wall degrading enzymes; WR, wheat bran and rice straw; L, submerged fermentation; FPKM, fragments per kilobaseof exon per million fragments mapped
