# Supplementary figures and images for: Differential transcriptomic profiling of filamentous fungus during solid-state and submerged fermentation and identification of an essential regulatory gene PoxMBF1 that directly regulated cellulase and xylanase gene expression
Source: Biotechnol Biofuels. 2019 Apr 30;12:103. doi: 10.1186/s13068-019-1445-4 (PMC6489320; doi:10.1186/s13068-019-1445-4)

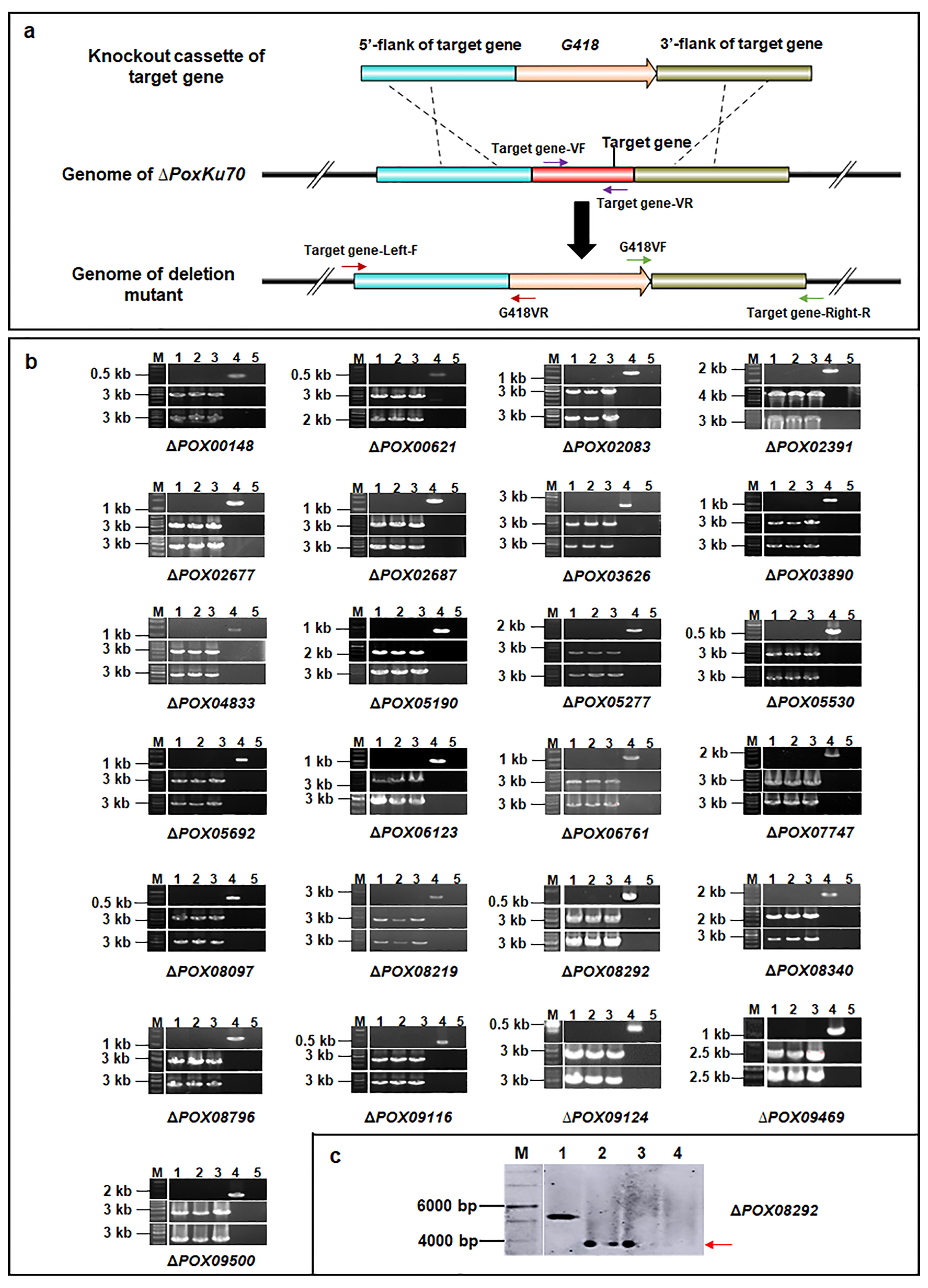

Supplement: Supplementary file 3 — Additional file 3: Figure S1. Confirmatory analysis of 25 deletion mutants constructed from the parental strain ΔPoxKu70. (a) Schematic illustration of deletion mutant construction. (b) PCR analysis. M, 1 kb DNA markers; Lanes 1–3, three transformants for each candidate gene; Lane 4, ΔPoxKu70; Lane 5, ddH2O. The top panel shows amplification of the region using primers Gene-Left-F/G418VR; the middle panel shows amplification of the region using primers G418VF/Gene-Right-R; the bottom panel shows amplification of the region of the target gene using primers GeneVF/GeneVR. (c) Southern hybridisation analysis of deletion mutant ΔPOX08292. M, 1 kb DNA markers; Lane 1, ΔPoxKu70; Lane 2, ΔPOX08292-2; Lane 3, ΔPOX08292-6; Lane 4, ΔPOX08292-12. [file 13068_2019_1445_MOESM3_ESM.tif]

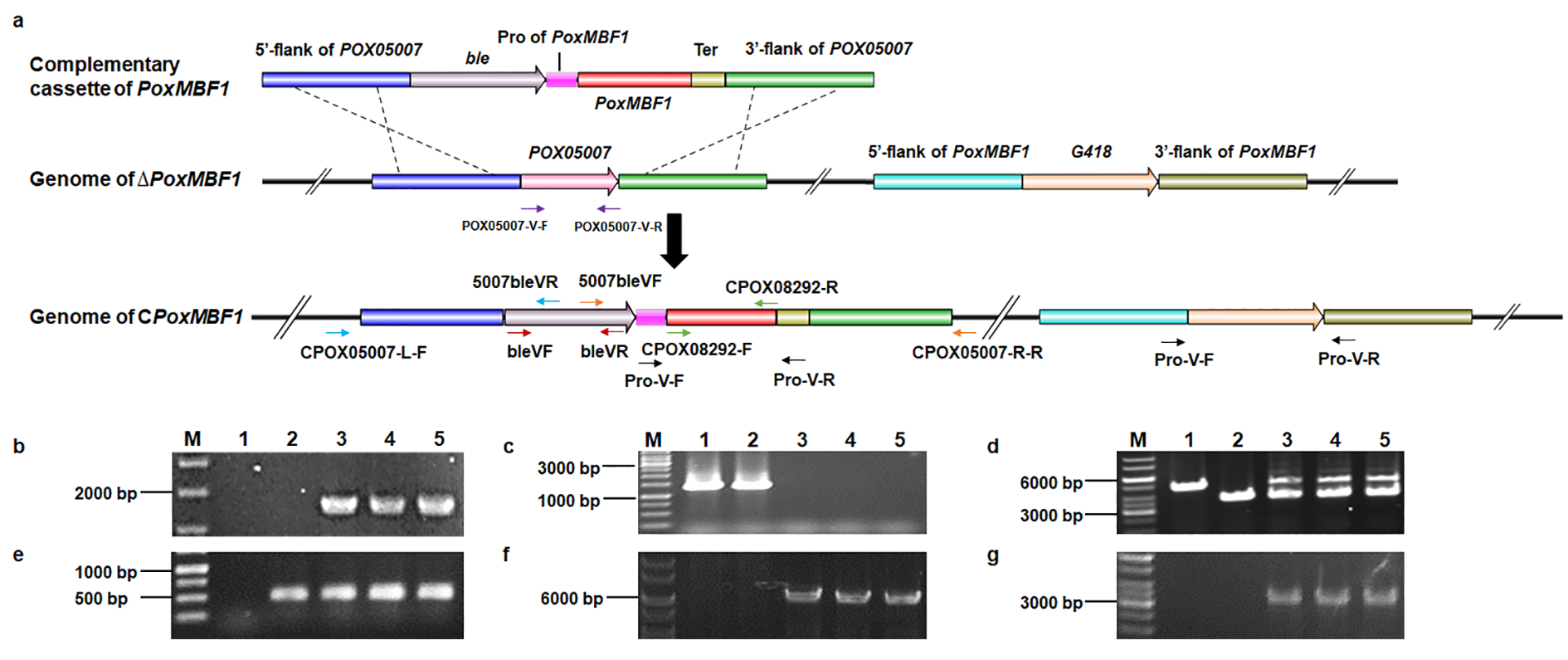

Supplement: Supplementary file 6 — Additional file 6: Figure S2. Confirmation of complementary strain CPoxMBF1. (a) Schematic illustration of complementary strain construction. (b–g) PCR analysis. M, 1 kb DNA markers; Lane 1, ΔPoxMBF1; Lane 2, ΔPoxKu70; Lanes 3–5, three transformants of CPoxMBF1; (b) PCR using primers bleVF/bleVR; (c) PCR using primers POX05007-V-F/POX05007-V-R; (d) PCR using primers Pro-V-F/Pro-V-R; (e) PCR using primers CPOX08292-F/CPOX08292-R; (f) PCR using primers 5007bleVF/CPOX05007-R–R; (g) PCR using primers CPOX05007-L-F/5007bleVR. [file 13068_2019_1445_MOESM6_ESM.tif]

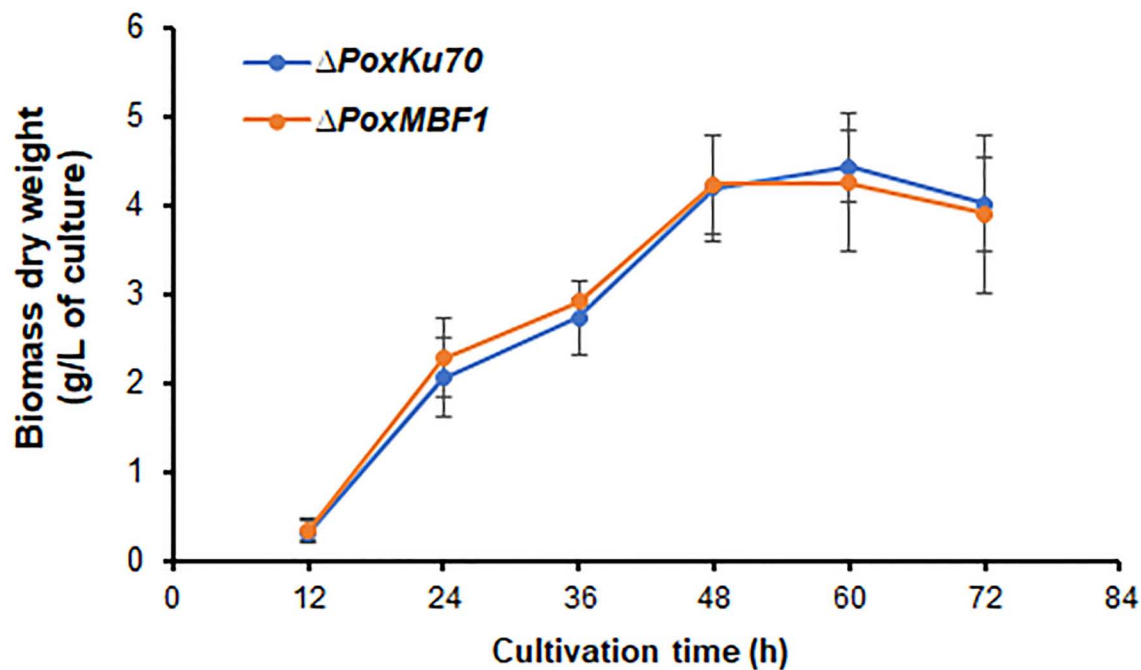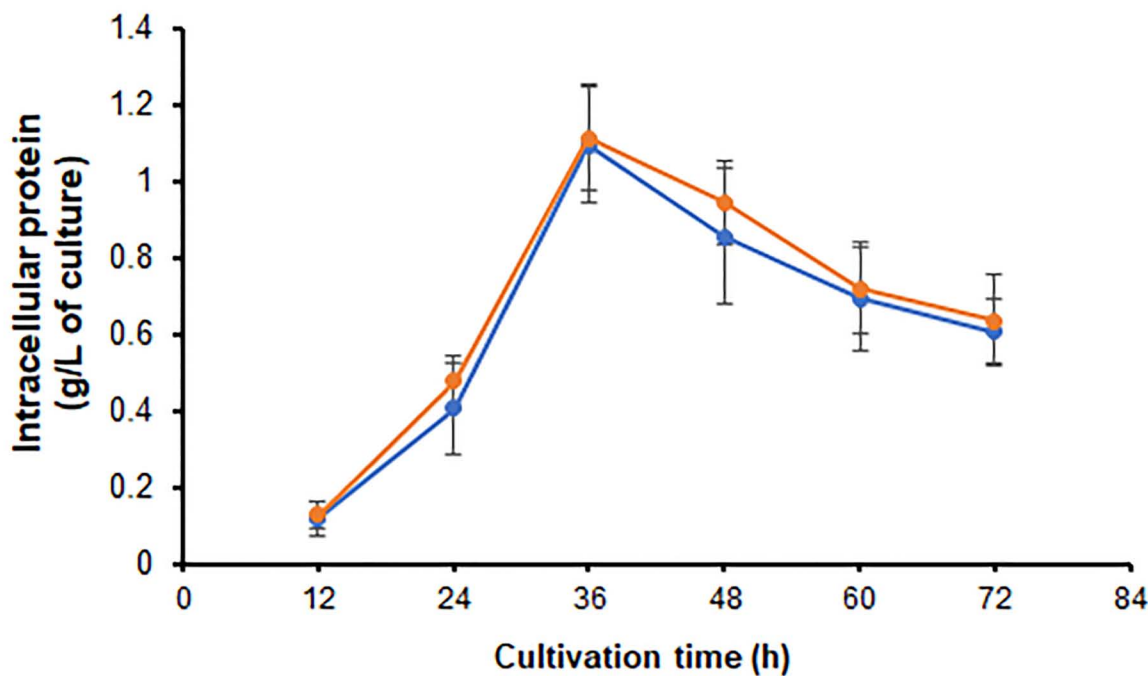

Supplement: Supplementary file 7 — Additional file 7: Figure S3. Growth curve of P. oxalicum mutants ΔPoxMBF1 and ΔPoxKu70 in glucose (a) and Avicel (b) media. [file 13068_2019_1445_MOESM7_ESM.pdf]
