# Supplementary material for: Differential transcriptomic profiling of filamentous fungus during solid-state and submerged fermentation and identification of an essential regulatory gene PoxMBF1 that directly regulated cellulase and xylanase gene expression
Source: Biotechnol Biofuels. 2019 Apr 30;12:103. doi: 10.1186/s13068-019-1445-4 (PMC6489320; doi:10.1186/s13068-019-1445-4)
Supplement: Supplementary file 4 — Additional file 4: Table S3. Primers used in this study. [file 13068_2019_1445_MOESM4_ESM.pdf]

Additional file 4: Table S3. Primers used in this study**.**

Primer name	Sequence（5'-3'）
**Primers used for the construction of deletion mutants**	
POX00148-L-F	GTAGTCGTACATGCGGTCGTTGAA
POX00148-L-R	GGTAATCCTTCTTTCTAGATTTGGAATTTTCGGCCCCG
POX00148-R-F	CAATATCATCTTCTGTCGACGCTGGACTCCGAAATCTTCTGA
POX00148-R-R	ATATCCGCCAGGCTCTTATGTA
POX00148-N-F	CAGGACATCATTGTTCACGGTAG
POX00148-N-R	GGTTGGCATCCTTATCATTTATT
POX00148-V-F	AGCGTCGCACAAGGTATG
POX00148-V-R	CCACGTTGTCTTCCTCCC
POX00621-L-F	CTTTTCGGTTCGGTCACT
POX00621-L-R	GGTAATCCTTCTTTCTAGAGTTTAGGGTGATACTGGGAGTT
POX00621-R-F	CAATATCATCTTCTGTCGACAATAAGAGCAGGAAAGAAAGA
POX00621-R-R	GGATACCAATGCCTACGAT
POX00621-N-F	AGGTGGTCATTTCCGATGAGGGTA
POX00621-N-R	CTGTCGGCAACCCCGTGAAATG
POX00621-V-F	ACACCATCATGTCCGACC
POX00621-V-R	CTTGACTCTCCCCCTTCC
POX02083-L-F	CCCCAGTGTCCCCCAGTA
POX02083-L-R	GGTAATCCTTCTTTCTAGATGCAAGTGAACAGTCCCGT
POX02083-R-F	CAATATCATCTTCTGTCGACGGGAGTGCTGGAGTCATCCCA
POX02083-R-R	CTAAGCCTGTGGTTGTTGTAGCC
POX02083-V-F	GTATGCCGAACATGAGAACCAGC
POX02083-V-R	GAGGCTTAAGTCCTGTTTCGGAA
POX02083-N-F	ATGCCGACGGAGAAAAAT
POX02083-N-R	CCACCTAAAGGCGAAACA
POX02391-L-F	ACATTGGTGGTGTCCTGG
POX02391-L-R	GGTAATCCTTCTTTCTAGATGTGGACTGGGATCTGCA
POX02319-R-F	CAATATCATCTTCTGTCGACGAGCCCCGCATTTGCGTG
POX02319-R-R	ACTCTGCTTAACACCGCCGAA
POX02319-N-F	GATTGACTACCCTTTCGTGACTG
POX02319-N-R	CTCCTTAATCAATGTGGCGAGATA
POX02319-V-F	GAGAGAAGAGAAAGCGGG
POX02319-V-R	GGATTTGGCGTCGTAATA
POX02677-L-F	ACAATCCGCCACCTAAAG
POX02677-L-R	GGTAATCCTTCTTTCTAGAGGTGCTCCACTGTCTAGTAGT
POX02677-R-F	CAATATCATCTTCTGTCGACAAGATCAAAGTCCTCATCCCGT
POX02677-R-R	ACGAGGAGCGATACCGACTT
POX02677-N-F	TCCCTGACTAGCGGACTCGTTA
POX02677-N-R	TTGTCTGGTGGTGTTATTGAGTCTA
POX02677-V-F	CGTTGCTCTCTGGTGATT

POX02677-V-R	GTAGTGTGGCTGGGTCTG
POX02687-L-F	ACTCGCATTTTTGCCGCT
POX02687-L-R	GGTAATCCTTCTTTCTAGAGAGGCTGGAGGGGAGGGT
POX02687-R-F	CAATATCATCTTCTGTCGACGCTTCTGACCTTGACGACC
POX02687-R-R	GGGAACCATCTCAGCGTA
POX02687-N-F	TCGGCTCAACAATCTCAATAAG
POX02687-N-R	TGACAATGAGTTGCGGTATGAAT
POX02687-V-F	GCTTCTATCCCGTTTCCA
POX02687-V-R	CTTCCACTTGTTTGCGTC
POX03626-L-F	TGACCGAGAACAAACGC
POX03626-L-R	GGTAATCCTTCTTTCTAGATGTGTTCCTCGCGATCT
POX03626-R-F	AATATCATCTTCTGTCGACGATTGCATTACCCATTGAC
POX03626-R-R	ATCAACATCGGCTCCAC
POX03626-N-F	CGGCAAATCCTCCCA
POX03626-N-R	CCAATCACCGAGCAAAAC
POX03626-V-F	AAGGGGTTCACGAAATGG
POX03626-V-R	ACCGTCGGTGGTGTTGA
POX03890-L-F	CCGAGCTGATGCTAAGAATGT
POX03890-L-R	GGTAATCCTTCTTTCTAGAGGTGTCCACGACACTGATGTT
POX03890-R-F	CAATATCATCTTCTGTCGACACCCCCTATTTCACCACTCGCCC
POX03890-R-R	CGAGGTTCGTTCTCCCCGCAAA
POX03890-N-F	CGACCCCTGTTGCTGTCTTTGTCC
POX03890-N-R	CGGTGGCGGTTTCGGTTGATGT
POX03890-V-F	ATACATCCACTGTCCGCACG
POX03890-V-R	GGCAAGTGAAAGGGAAGGAC
POX04833-L-F	TGTTGGGCCATTCCCGCTAG
POX04833-L-R	GGTAATCCTTCTTTCTAGAGATGAGAGTTGACAAGGGGGCG
POX04833-R-F	CAATATCATCTTCTGTCGACCTTTTCCTTCTCTTTTTTGCT
POX04833-R-R	GGAGGTTTGCGAATACCACTA
POX04833-N-F	TAGCACCGACTTCCACATTG
POX04833-N-R	GGTTTGCGAATACCACTACTAC
POX04833-V-F	CCGTTCAGCCGTCGTTGT
POX04833-V-R	CGATTTGGCCCGAGTTCC
POX05190-L-F	ACCGACCGGCCCGAAAGTA
POX05190-L-R	GGTAATCCTTCTTTCTAGAGTTTGGGAGCGTGAGGGGAAT
POX05190-R-F	CAATATCATCTTCTGTCGACGACTTGCGTTTGCAAAAACGAGAT
POX05190-R-R	AGACTGGTATGAGGGCGTGATG
POX05190-N-F	ACGGAGTGATCTCCAGTATCGTT
POX05190-N-R	GAGATGAGGCCAACACTGTAAGG
POX05190-V-F	CTGAGCATCGGCGAAGACAT
POX05190-V-R	GGCGACAAGGATTGAGGTGG
POX05277-L-F	CTCAACGGTGCGGTGATAAAGT
POX05277-L-R	GGTAATCCTTCTTTCTAGACTTCTAGATTTCTAGCGCGATCGG

POX05277-R-F	CAATATCATCTTCTGTCGACATGCTTCCCCCTCCCTTCT
POX05277-R-R	ATTGGGGCGGCTCAAGTTC
POX05277-N-F	AAGCCGAGTTGGTGCAAATATCGC
POX05277-N-R	GGTCAGCGTGGCTCGCTTTAGCT
POX05277-V-F	TCCAAGGCGTCGCAATCC
POX05277-V-R	GGCAATGAAAAGCGGGACA
POX05530-L-F	CGACCTCAGACAGAACCAATAGT
POX05530-L-R	GGTAATCCTTCTTTCTAGATTCTTGACCTTGCTTTTGCCT
POX05530-R-F	CAATATCATCTTCTGTCGACCTGATTGACACGAGAGCGCAT
POX05530-R-R	TCCACATCTGCGTTTGACAGC
POX05530-N-F	CCAATCCACAGCGGTAGAGACGTG
POX05530-N-R	CCAACCACCGAAAAAGACACCTCA
POX05530-V-F	GCGTAAGTCGTCACTCGGTC
POX05530-V-R	GATGCTACGGTTGGTGTGAG
POX05692-L-F	AGCCCGAGAGTCTGTCAC
POX05692-L-R	GGTAATCCTTCTTTCTAGAGGTGAAGGGTTGAGATCAC
POX05692-R-F	CAATATCATCTTCTGTCGACGCAGACGGATGCATCCTTTGGTGT
POX05692-R-R	GAGACAGTCAAGGGAGCCGTCACA
POX05692-N-F	GAGTTCCCGTGTGCTCCAGTCGC
POX05692-N-R	GAATCCTACCGCCGACTCTCCGC
POX05692-V-F	ACGGCTGTCGTTGGTCTTT
POX05692-V-R	AGAGGCTGACGGGGTAAGA
POX06123-L-F	CCAGTGAAGTGGGGATTTTAC
POX06123-L-R	GGTAATCCTTCTTTCTAGATTTGAGTGTTGGGGGATATG
POX06123-R-F	CAATATCATCTTCTGTCGACAGAGGGAAAGGCCAATTGC
POX06123-R-R	GATGCGGACGACACCAGC
POX06123-N-F	GCTGCTACGAGAATGAAAGTGAGT
POX06123-N-R	AACCTCGCTTGTCCCTGGTATATC
POX06123-V-F	TGACAGAACTGGAGCATCGC
POX06123-V-R	CCGAAGAAAATGTCCACCCA
POX06761-L-F	TCACGGGCTCACCTCACCTGTTCG
POX06761-L-R	GGTAATCCTTCTTTCTAGAGTTGGCCGGGTGTCGCAGTGG
POX06761-R-F	CAATATCATCTTCTGTCGACCGCCTGCCAGCGAAATCTT
POX06761-R-R	TCCTCCAACACCGACCTAAACC
POX06761-N-F	AATCCCAAGTCCCCGCAATGTC
POX06761-N-R	AACAGGTCCACAGAAACCACAGCAG
POX06761-V-F	GAACGGCGTCCGATGCTT
POX06761-V-R	CGACTTTCCCGACAGGCAC
POX07747-L-F	TCAGTGCTCTGCTGCTACGAC
POX07747-L-R	GGTAATCCTTCTTTCTAGAGTGAGTTGCATTCCATGAAGAAT
POX07747-R-F	CAATATCATCTTCTGTCGACGGTATATATGCATGGATAGATGTAT
POX07747-R-R	CAAAAAGTCCTCGGTCGT
POX07747-N-F	CAGTTGGAGATTGACACTTTACACG

POX07747-N-R	TAGATAGTCCACGCAATGATGTAGA
POX07747-V-F	AACGACGCTTCTCCCCACT
POX07747-V-R	ACGGTGCTCGTCCAAAGTG
POX08097-L-F	ACCGCCGAGTTGTCCTATGA
POX08097-L-R	GGTAATCCTTCTTTCTAGAACTGTCTGTAACGTGGTGGTCGT
POX08097-R-F	CAATATCATCTTCTGTCGACGTCGGATCGGTTCCTTGCTGC
POX08097-R-R	TTGCTTTCATTTTCTTTGGGTCCTT
POX08097-N-F	CCCCATCTTCTCCAACTCCATAG
POX08097-N-R	ATTCATTCACATTCAAATCGTAACC
POX08097-V-F	ATTCCACTACTCCGACTACGCAC
POX08097-V-R	GTCCTGACGAGGGAAGCCAT
POX08219-L-F	CTCGTCCAGCCCGTTTTTC
POX08219-L-R	GGTAATCCTTCTTTCTAGATTTATCGTGGCAAATTCGTTTG
POX08219-R-F	CAATATCATCTTCTGTCGACAAGACGGAAATTGATAATCCCAGC
POX08219-R-R	AGACGAGGAGGATGCGATGG
POX08219-N-F	CATGAGTGACCTACCAGTCGCTTTG
POX08219-N-R	TTCCCGCTCTCGCCCTCTTTG
POX08219-V-F	TCGCATTCGTTCGCAGTAAG
POX08219-V-R	TTGTTGTAAGGGCTGCTGTTG
POX08292-L-F	GGACACAAAGTTCCTGCTG
POX08292-L-R	CACCCACACTTTCTTCAAA
POX08292-R-F	GCCACGCAGCCTCTGCTACAA
POX08292-R-R	TGGCACACTTCCCATTCT
POX08292-N-F	GGGTTGAGCGGGGCTCCATCGA
POX08292-N-R	GGGGAGGATATGCATATCGCGA
POX08292-V-F	ATGAGCGACTGGGACACTGTTA
POX08292-V-R	TTATTTCTTCTTGGGGAACT
POX08340-L-F	CGAACCATCGCCAAGTCCTACATTA
POX08340-L-R	GGTAATCCTTCTTTCTAGATGACTTTGCGTGTGTCATCGCTC
POX08340-R-F	CAATATCATCTTCTGTCGACGCCGCAGAATAGCCCGGAATC
POX08340-R-R	CCGCCGTATTTACAATAGTACCCCA
POX08340-N-F	GGTGTCCTTCATCGGGAACCAGA
POX08340-N-R	AGATGATTCCCTCCTCCGTCTCG
POX08340-V-F	CGGCTCAAGATAAGCGAAGG
POX08340-V-R	CTCGGAGTACAGTCATGGCGT
POX08702-L-F	GGTCTCATGGTCCACGGAT
POX08702-L-R	GGTAATCCTTCTTTCTAGAGCTGAGTACAGGTATGGTGACC
POX08702-R-F	CAATATCATCTTCTGTCGACATGTCCTTTCAATTCTGTAGAGTCG
POX08702-R-R	CCCACCCTTTACCTGTCATACG
POX08702-N-F	GGACAAAGTATCGGAATCGGTTA
POX08702-N-R	GCGTCCATGTTAATTGCTCTCTG
POX08702-V-F	TTTATCTGCCTGCCGCTGA
POX08702-V-R	CGGATAGAATAGAAGCATCGCA

POX08796-L-F	GGGCTCGGACGGATGGGTGACG
POX08796-L-R	GGTAATCCTTCTTTCTAGACGCGCATGGGGGGCGGAAG
POX08796-R-F	CAATATCATCTTCTGTCGACTCTCGAGGCTGTTTGCAAGCAC
POX08796-R-R	GTCAACGATACTACGCTACTTCCCA
POX08796-N-F	TTCTTGGACGGGATCTCACGAT
POX08796-N-R	TCTACCCCATCCTATCTACCACCG
POX08796-V-F	AAGAAGTGCGATGAGACCCG
POX08796-V-R	TCCAGACACGAGCCAACCAC
POX09116-L-F	GCGGTGTGGAATGCTACTGTGGC
POX09116-L-R	GGTAATCCTTCTTTCTAGACGCGGTGGATTGCACAAGTACA
POX09116-R-F	CAATATCATCTTCTGTCGACTAGCCGCTTAACATACATCTAGCC
POX09116-R-R	GCAGATGGTCCTGTCGTCG
POX09116-N-F	CGTCTGCCACCGTGAGGATTGGTAAAT
POX09116-N-R	GAAAGCCGCAGTGACGGTCGCA
POX09116-V-F	ACACCTACCCAGTGGAGCGA
POX09116-V-R	GGACTGTGGTTCCAAATGCC
POX09124-L-F	CTGCGTGTAGGTAACTCGTAGC
POX09124-L-R	GGTAATCCTTCTTTCTAGACGTGGGTGAGTCGCTTC
POX09124-R-F	AATATCATCTTCTGTCGACCAACAGTGGGCAGGGAGA
POX09124-R-R	CACAACCCGCCATTACCAT
POX09124-N-F	CGACTCCGCAACGATACAA
POX09124-N-R	CAAAGGACCAGTTCCGCTAT
POX09124-V-F	ATGGAGGGGGGTTCAGG
POX09124-V-R	TCAGTCGTCGGTAGCGG
POX09469-L-F	GAGCCTGAAGTTTCTTGAATACG
POX09469-L-R	GGTAATCCTTCTTTCTAGATTTTATGAAGAGTTGAGGCTCATC
POX09469-R-F	AATATCATCTTCTGTCGACATTTCATCATGGTAGATCCTGATTA
POX09469-R-R	ACAGCCCGCAGTCCTAATG
POX09469-N-F	TGGTCGTCAGTTGAAGCGTG
POX09469-N-R	CAGTCCTAATGGCACGCAC
POX09469-V-F	AAGATGTGGATGAAGTAGTTGCC
POX09469-V-R	CCAGCAATGTCCAAGAGCC
POX09500-L-F	TGACCGCTTTCCTCCCCCACA
POX09500-L-R	GGTAATCCTTCTTTCTAGAGGCGCCTCCACTCCATCAAGCT
POX09500-R-F	CAATATCATCTTCTGTCGACGCTATGCTGGGTTGCCTTTTAT
POX09500-R-R	GAAGTGCAAGCCGAATTCAGAGT
POX09500-N-F	CAAGGATGGTAATATGCCTCACG
POX09500-N-R	ATATGAAGCACACCATTCTCTCTGC
POX09500-V-F	CATACACGGTGCGGTTGAGC
POX09500-V-R	CCTTCGGCGTCTTTTTCGTT
G418-F	TCTAGAAAGAAGGATTACC
G418-R	GTCGACAGAAGATGATATT
G418-V-F	GTGAATGCTCCGTAACACCCAAT

G418-V-R	CGCTACTGCTTACAAGTGGGCTGAT
**Primers used for the complementation**	
POX05007-R-F	AGAGGTGGAAACATAGATGGTAAATGTAGATCACCAAGTTGCA
POX05007-R-R	TACCCGCCAATATATCCTGTCAACGTCAATCACTTCCTGCG
POX05007_Ble-F	ACGCTTAGACAACTTCGATCGACTGGAACAGGTGACCCCTATGG
POX05007_Ble-R	CCAATATATCCTGTCAAAAGCTTCTGGATCTCAACAGCGGTAAG
CPOX01907-F	GATCTTACCGCTGTTGAGATCCAGTTTGACGCCTATCACTACTTTACCC
CPOX01907-R	GCCAATATATCCTGTCAAGGGCCCTTACCATCTATGTTTCCACCTCTG
CPOX05007-L-F	GGAACAGGTGACCCCTATGGA
CPOX05007-R-R	TGAAAGAATCCACCGTCGCAATCGT
5007bleVF	ATCTGGAAGAGGTAAACCCGAA
5007bleVR	CAGACAGGAACGAGGACATTATT
bleVF	GAAAGAAGGATTACCTCTAAACAAG
bleVR	GGTAAGATCCTTGAGAGTTTTCGCC
CPOX08292-F	ATGAGCGACTGGGACACTGTTACC
CPOX08292-R	TTATTTCTTCTTGGGGAACTTCTC
Pro-V-F	TTTGACGCCTATCACTACTTTACCC
Pro-V-R	TTACCATCTATGTTTCCACCTCTG
POX05007-V-F	ATGGTTGTCTTCAGCAAGGTTA
POX05007-V-R	CTATGCCTGAGCAGCGAAA
**Primers used for the probe amplification in Southern hybridization**	
POX08292-probe-F	GTGGAAACATAGATGGTAAC
POX08292-probe-R	TAGGTATCTCATCACAAAC
**Primers used for RT-qPCR analysis**	
RT-actin-F	CTCCATCCAGGCCGTTCTG
RT-actin-R	CATGAGGTAGTCGGTCAAGTCAC
RT-POX05587-F	GTACTTGCGATCCTGATGGG
RT-POX05587-R	CCACGGTGAAGGGAGACTTG
RT-POX01166-F	CGATACTACGGCAACATCATCAC
RT-POX01166-R	AGGCACCAGTCCACGAGTTT
RT-POX02740-F	GTTCAGTTCCTGATGGAAAGATTG
RT-POX02740-R	CATAACCGCCTGCTTGAGTG
RT-POX05571-F	AACCTGGAAGAACGGCACC
RT-POX05571-R	CCTTGTCACAGTCATCGGAGC
RT-POX06835-F	GTGCTGGATGGGAACAGGA
RT-POX06835-R	TACGAACGCCGAGAGGAGA
RT-POX06783-F	TGAGCCCAGGACCATCAACTT
RT-POX06783-R	TACCCTTGCTTTTGCCGCC
RT-POX08484-F	ACAAGCACACGCAGGTCAA
RT-POX08484-R	CGCTGAAGTGGTTGGCAGT
**Primers used for EMSA assay**	
POX05587-EMSA-F	CATCTAACATTTCAGAGTGCCCT
POX05587-EMSA-R	6-FAM-CAGGGAGGAGAGGAGGAGAG

POX05571-EMSA-F	GATCCTTGTCTGTTTGGCACG
POX05571-EMSA-R	6-FAM-TGTATGTCTTCAGGGACGGGTT
POX06835-EMSA-F	ATCTCGGGCCCAGCGAGGTTG
POX06835-EMSA-R	6-FAM-TCGCAAGTCAACTGGGGGT
POX06783-EMSA-F	GCGAGAAGAAAAGTCTTAAG
POX06783-EMSA-R	6-FAM-TGTTTTATTCAAAGGAACGATG
POX01166-EMSA-F	TTGATGCGCGCTCATCTTCGATG
POX01166-EMSA-R	6-FAM-GATGAGTGTAAGCCTCTCTGGGTG
POX08484-EMSA-F	AATTCCAAGACCAGTCAAAGC
POX08484-EMSA-R	6-FAM-CCATCGCAAGCTTTTGCCCA
POX02704-EMSA-F	CACACAATCGGAGCCAGCAGTTC
POX02704-EMSA-R	6-FAM-GGAAGCTTCAGGATGACGGCAA
