# Supplementary material for: Differential transcriptomic profiling of filamentous fungus during solid-state and submerged fermentation and identification of an essential regulatory gene PoxMBF1 that directly regulated cellulase and xylanase gene expression
Source: Biotechnol Biofuels. 2019 Apr 30;12:103. doi: 10.1186/s13068-019-1445-4 (PMC6489320; doi:10.1186/s13068-019-1445-4)
Supplement: Supplementary file 5 — Additional file 5: Table S4. Candidate regulatory genes knocked out in P. oxalicum. [file 13068_2019_1445_MOESM5_ESM.pdf]

Additional file 5: Table 4. List of 25 candidate regulatory genes newly knocked out in *Penicillium oxalicum* in this study

Name	**InterPro annotation of predicted protein**	**Domain description of predicted protein**
POX00148	IPR007087	Zinc finger, C2H2-type
POX00621	IPR009395	GCN5-like 1
POX02083	IPR001138	Zinc finger, Zn2Cys6 type
POX02391	IPR000116<br>IPR000637<br>IPR002740<br>IPR015947<br>IPR017956	High mobility group protein HMGA;<br>HMG-I/HMG-Y;<br>EVE domain;<br>PUA-like domain;<br>AT hook
POX02677	IPR001766<br>IPR011991	Forkhead transcription factor;<br>Winged helix repressor DNA-binding domain
POX02687	IPR001005<br>IPR009057	Myb;<br>Homeodomain-like;
POX03626	IPR001138<br>IPR007219	Zinc finger, Zn2Cys6 type;<br>Fungal_Trans
POX03890	IPR001138<br>IPR007219	Zinc finger, Zn2Cys6 type;<br>Fungal_Trans
POX04833	IPR001138<br>IPR007219	Zinc finger, Zn2Cys6 type;<br>Fungal_Trans
POX05190	IPR007087	Zinc finger, C2H2-type
POX05277	IPR001138	Zinc finger, Zn2Cys6 type
POX05530	IPR007219	Fungal_Trans
POX05692	IPR008967	p53-like transcription factor
POX06123	IPR001138	Zinc finger, Zn2Cys6 type
POX06761	IPR001138	Zinc finger, Zn2Cys6 type
POX07747	IPR007219	Fungal_Trans
POX08097	IPR007087	Zinc finger, C2H2-type
POX08219	IPR007219	Fungal_Trans
POX08292	IPR001387	Helix-turn-helix type 3
POX08340	IPR007219	Fungal_Trans
POX08796	IPR001138	Zinc finger, Zn2Cys6 type
POX09116	IPR011991	Winged helix repressor DNA-binding domain
POX09124	IPR001138	Zinc finger, Zn2Cys6 type
POX09469	IPR011991	Winged helix repressor DNA-binding domain
POX09500	IPR001138<br>IPR007219	Zinc finger, Zn2Cys6 type;<br>Fungal_Trans
